# Dispiroindolinone–Glutarimide Conjugates: Synthesis and Evaluation as Potential Hetero-PROTACs for p53 Reactivation

**DOI:** 10.3390/molecules31101602

**Published:** 2026-05-10

**Authors:** Vladislav S. Polyakov, Yuri K. Grishin, Viktor A. Tafeenko, Ekaterina S. Ivanova, Sofya S. Pogodaeva, Daniil V. Moldavskii, Alexander A. Shtil, Elena K. Beloglazkina

**Affiliations:** 1 Department of Chemistry, M.V. Lomonosov Moscow State University, Leninskie Gory 1-3, 119991 Moscow, Russia; vvladislavpolyakov@gmail.com (V.S.P.); ykgris@mail.ru (Y.K.G.); tafeenko-victor@yandex.ru (V.A.T.); 2 Institute of Experimental Oncology and Carcinogenesis, Blokhin National Research Center of Oncology, 24 Kashirskoye Shosse, 115522 Moscow, Russia; ekaterinakolotova@mail.ru (E.S.I.); shtilaa@yahoo.com (A.A.S.); 3 Institute of Cyber Intelligence, National Research Nuclear University MEPhI, 31 Kashirskoye Shosse, 115409 Moscow, Russia; 4 Center for Molecular and Biological Technologies, ITMO University, 9 Lomonosov Street, 197101 Saint-Petersburg, Russia; pogodaeva@scamt-itmo.ru (S.S.P.); daniilmoldavskiy2@gmail.com (D.V.M.)

**Keywords:** spiroindolinones, glutarimide, azide–alkyne (3+2) cycloaddition, thiohydantoin, imidazolones

## Abstract

A convergent scheme for the preparation of conjugates with the dispiroindolinone-pyrrolidine-thioimidazolone and glutarimide moieties connected via a triazole-containing linker is proposed. Target conjugates were synthesized by azide–alkyne (3+2) cycloaddition reactions between propargylthio-substituted dispiroindolinone-pyrrolidine-imidazolones and an azido-glutarimide derivative. The starting compounds were available isothiocyanates, glycine, substituted benzaldehydes, chloroacetamide, and ethyl acrylate. The key azide–alkyne (3+2) cycloaddition step was carried out using TBTA as a catalyst, achieving >70% product yields. The resulting bifunctional compounds contained a fragment of dispiroindolinone (a p53-MDM2 interaction inhibitor) and glutarimide, a ubiquitin ligase ligand. The obtained dispiroindolinone-glutarimide conjugates were tested for their potential as hetero-PROTAC compounds for p53 reactivation. Individual conjugates showed preferential cytotoxicity against HCT116 colon carcinoma cells (wild-type53) compared to the isogenic HCT116p53^−/−^ subline.

## 1. Introduction

The MDM2 protein, an E3 ligase of the tumor suppressor p53, is overexpressed in many cancer types. As a result, p53 loses its ability to trigger apoptosis [1,2,3]. In the last 20 years, various compounds of the imidazoline and indolinone classes [4,5] have been proposed to inhibit the p53-MDM2 interaction, thereby activating p53, including nutlins [6,7], spiroindolinones [8,9,10], and dispiroindolinones [11] containing imidazolinone or thioimidazolinone fragments [9,12]. However, most known MDM2-p53 inhibitors cause numerous side effects, partly because high concentrations are required to disrupt protein–protein complexes.

A promising strategy to overcome the side effects of antitumor drugs is the PROTAC (PROteolysis Targeting Chimera) approach [13]. This strategy allows for a significant reduction in the amount of the compound used to degrade the unwanted proteins. The PROTAC molecules are the conjugates consisting of two functional fragments connected by a linker. One functional fragment is capable of binding to the target protein, and the other one fragment binds to the E3 ubiquitin ligase. As a result, the target protein is ubiquitinated for subsequent proteasomal cleavage. Then the PROTAC molecule is released and can participate in the next round of target ubiquitination. In this scenario, the cellular effect is achieved along with reduced drug concentrations and limited side effects [14,15].

Homo-PROTACs can be used if the functional fragment is a ligand for the target protein as well as for a specific ubiquitin ligase; in this case, it is sufficient to connect two identical functional fragments with linkers to initiate protein degradation [13,14,15,16,17]. In hetero-PROTACs, one functional fragment acts as a ligand for the target protein, and the second one binds to the ubiquitin ligase [18,19,20,21]. Typically, thalidomide [22,23,24,25,26] or similar molecules containing the fragments of glutarimide and phthalimide [27,28], or glutarimide alone [29,30], can be used as ubiquitin ligase binders.

**Figure 1:**
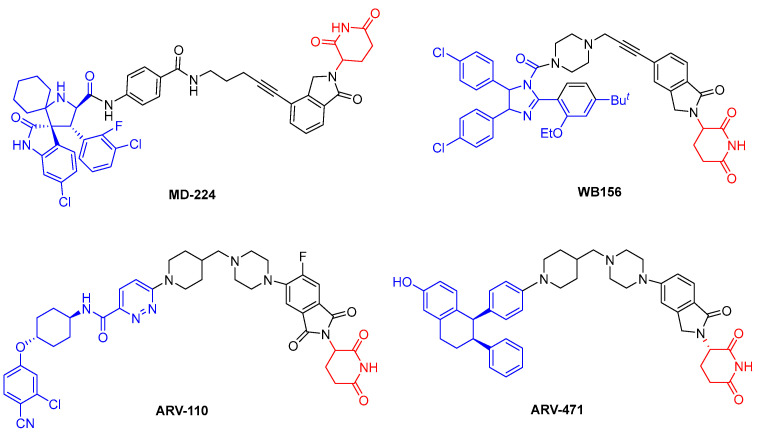
Structures of glutarimide-containing hetero-PROTAC compounds in development and in clinical trials [18,20,31,32,33]. The fragment that binds to the target protein is blue, the fragment that binds to the ubiquitin ligase is red and a spacer is black.

Currently, several PROTAC-based drug candidates are in various stages of clinical trials: three are in phase III, 11 compounds are in phase II, and more than 30 are in phase I (according to [https://www.biochempeg.com/article/434.html] accessed on 29 May 2025). Compounds such as **ARV-110** [31] and **ARV-471** [32] for prostate and breast cancers are currently undergoing phases II and III, respectively (Figure 1). However, the synthesis of these molecules is a highly complex problem. The original method for the synthesis of **ARV-471** (Vepdegestrant) involves 16 stages, which leads to high costs and limited availability of the final compound.

**Figure 2:**
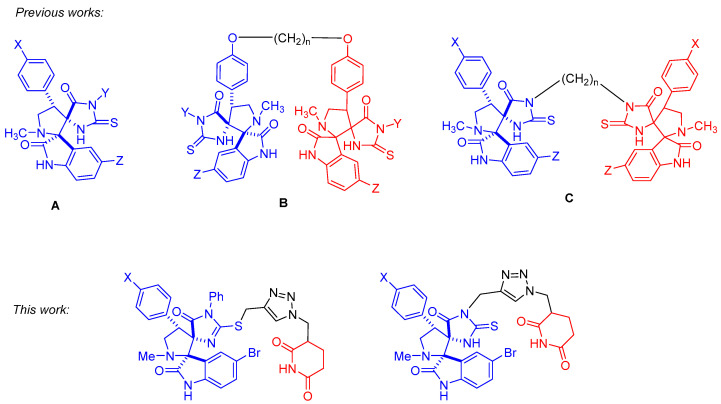
Structures of dispiroindolinone-pyrrolidine-thioimidazolones **A** [9,34,35,36,37], bis-dispiro-indolinone-pyrrolidine-thioimidazolones **B**, **C** [38,39], and the compounds synthesized in this work.

Previously, we synthesized a series of dispiroindolinone-type inhibitors of the p53-MDM2 interaction with significant cytotoxicity against HepG2 liver, LNCaP prostate, and MCF-7 breast cancer cell lines [9,34,35,36,37] (Figure 2, compounds **A**). Since MDM2 is a specific E3 ubiquitin ligase for p53, the dispiroindolinone moiety may be potentially utilized to generate homo- and hetero-PROTAC molecules. In our dispiroindolinone-based homo-PROTACs **B** and **C** ([38,39], Figure 2), two identical dispiroindolinone-pyrrolidine-thioimidazolone moieties were connected via a polymethylene linker. However, these compounds did not demonstrate a significant increase in cytotoxicity compared to dispiroindolinone **A**, indicating the need to change the overall design of the target molecules.

In the present study, we report the design and synthesis of novel small-molecular-weight conjugates containing fragments of the MDM2 ligand, dispiroindolinone-pyrrolidine-thioimidazolone, and the E3 ligase ligand, glutarimide, connected via a triazo-containing linker (Figure 2). The initial cytotoxicity testing of the synthesized compounds on human carcinoma cells carrying wild-type p53 in comparison with the isogenic p53-null sublines was also carried out.

The spirooxindole moiety is widespread in natural and biologically active compounds; the synthesis of spirooxindoles is the subject of a recently published monograph [40]. One of the most convenient methods for preparing indolinones spiro-fused to the pyrrolidine ring is the 1,3-dipolar cycloaddition of azomethine ylides to indolinone derivatives containing an exocyclic C=C bond. Such reactions typically proceed chemo-, regio-, and diastereoselectively, with good yields of the target compounds [41,42,43,44]. Azomethine ylides for these reactions are usually prepared in situ by reacting carbonyl compounds with amino acids or their derivatives [41,42,43,44,45,46,47,48]. We recently proposed [34] a convenient method for obtaining dispiroindolinone derivatives, including enantiomerically pure ones [49], by cycloaddition reactions of azomethine ylides with (*Z*)-5-arylmethylidene-substituted 2-thiohydantoin derivatives. An additional advantage of the synthesis of spiro-pyrrolidinindolinones by the addition of azomethine ylides to indolinone derivatives is their environmental friendliness—in most cases, the solvent for these reactions is ethanol, whereas only carbon dioxide and water are formed as by-products. Therefore, reactions of azomethine ylides with 5-arylidene thiohydantoins were chosen to assemble the dispiroindolinone framework.

## 2. Results and Discussion

### 2.1. Synthesis

**Scheme 1:**
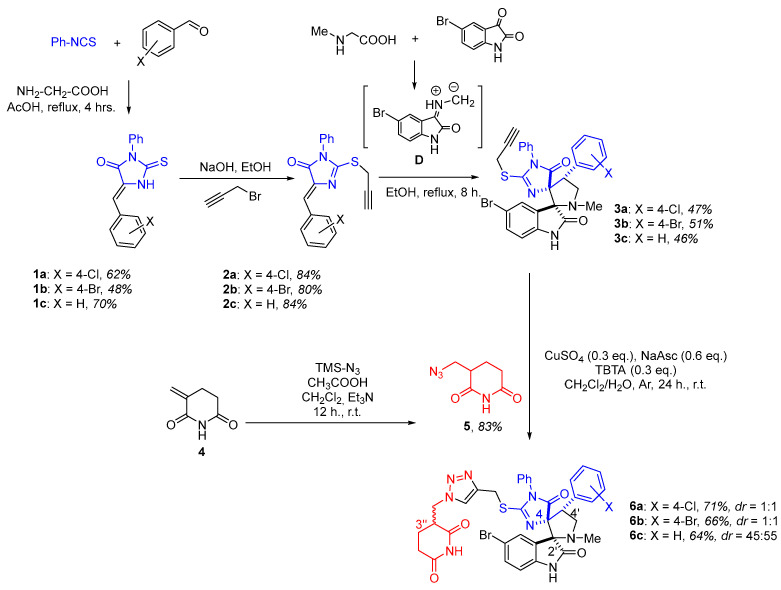
General scheme of synthesis of dispiroindolinone-glutarimide conjugates **6a**–**e**.

**Scheme 2:**
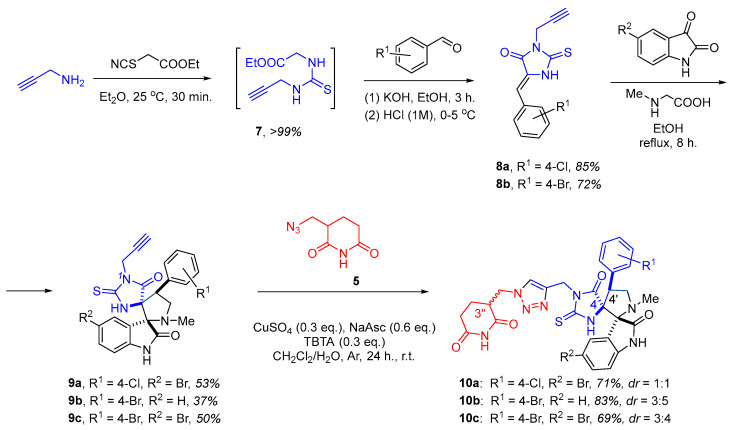
General scheme for synthesis of dispiroindolinone-glutarimide conjugates **10a**–**c**.

We propose simple and convenient methods for the preparation of two types of dispiroindolinone-glutarimide conjugates with triazole-containing linkers. Conjugates **6** (Scheme 1) had a dispiroindolinone-pyrrolidine-thioimidazolone fragment in their structure, connected to a glutarimide moiety through the sulfur atom of the thioimidazolone cycle. In molecule **7** (Scheme 2), dispiroindolinone was attached to glutarimide via the thioimidazolone N(3) nitrogen atom.

Compounds **6a**–**c** were obtained by a convergent approach starting from available 5-arylidene-2-thiohydantoins **1a**–**c** and 3-methylidene glutarimide **4** (Scheme 1). Thiohydantoins **1a**–**c** were synthesized from phenyl isothiocyanate, the corresponding benzaldehydes, and glycine according to the previously reported procedure [50]. S-Propargylation of thiohydantoins **1** to obtain 2-propargylthioimidazolones **2a**–**c** was carried out similarly to the procedure described for 5-pyridylmethylene-2-thioimidazolones, with minor modifications [51]. At the final step of the assembly of the dispiroindolinone functional fragment, compounds **2** were introduced into regio- and diastereoselective (3+2) cycloaddition reactions with azomethine ylide **D**, generated in situ from isatin and sarcosine, similarly to the synthesis of compounds **A** (Figure 2). The resulting products **3** were isolated by column chromatography. The selectivity of the reaction was confirmed by a single set of signals in ^1^H and ^13^C NMR spectra, containing characteristic three-spin system signals of protons of the central pyrrolidine ring in the range of 3.4–4.1 ppm (Supplementary Information). For compound **3b**, the complete assignment of signals in the NMR spectra was carried out using two-dimensional correlation methods HMBC, HSQC (see Supplementary Information). The relative 2′S*,4R*,4′R* configuration of compounds **3** is consistent with the one determined for the products of azomethine ylide addition to 5-arylidene-2-thioimidazolones [9,34,35,36,37].

The starting 3-methylenepiperidine-2,6-dione **4** was synthesized according to the previously reported procedure [52,53] from chloroacetamide and ethyl acrylate. 3-(Azidomethyl) piperidine-2,6-dione **5** was prepared by Michael addition of trimethylsilyl azide to compound **4** in a mixture of acetic acid and triethylamine using the procedure reported previously [29].

To obtain target conjugates **6** via a click reaction, we initially employed a standard azide–alkyne (3+2) cycloaddition procedure between alkyne **3a** and azide **5** using a CuSO_4_/NaAsc catalytic system in a CH_2_Cl_2_/H_2_O mixture. However, this reaction yielded no conversion of the starting spiro compound **3** (as monitored by TLC). Therefore, we modified the conditions by adding tris(benzyltriazolylmethyl)amine (TBTA) and conducting the reaction under an argon atmosphere to prevent Cu(I) oxidation. By varying the TBTA quantities, we found that the optimal amount was 0.3 equivalents relative to the starting spiro derivative **3**; with less TBTA, the yield decreased, and further increases in TBTA did not improve the yield of the target product. We also varied the reaction temperature (from 10 to 40 °C) and the amount of solvent; the optimal reaction procedure was identified as follows: 0.3 equiv. TBTA, 0.3 equiv. CuSO_4_×5H_2_O in 200 μL of deionized water, and 0.6 equiv. sodium ascorbate in 200 μL of deionized water were added to the reaction mixture containing 1 equiv. of terminal alkyne **3** and 1 equiv. of α-azidomethyl glutarimide **5;** stirring was carried out at room temperature for 24 h under an argon atmosphere.

Once the conditions were optimized, we synthesized a series of conjugates **6a**–**c** in reasonable yields (64–72%) and isolated them using silica gel column chromatography. The structures were confirmed by ^1^H and ^13^C NMR spectroscopy and high-resolution mass spectrometry. Since **3a**–**c** contain three chiral carbon centers (C2′, C4, C4′ atoms; Scheme 1) and azidomethyl glutarimide **5** also contains a chiral center at the C3 atom, all conjugates **6a**–**c** formed by combination of these structural fragments were isolated as the mixtures of diastereomers with 2′S*,4R*,4′R*,3″R* and 2′S*,4R*,4′R*,3″S* relative configurations (*ca* 1:1). Due to the presence of diastereomers, two sets of signals were observed in the NMR spectra of **6**. A complete assignment of signals in the ^1^H and ^13^C NMR spectra using two-dimensional HMBC, HSQC, and ^1^H−^1^H COSY techniques unambiguously confirmed the structure of the product for compound **6c** (Supplementary Information).

For the synthesis of conjugates **10a**–**c**, N(3)-propargyl-substituted 2-thiohydantoins **8a**,**b** were obtained in the first stage (Scheme 2). The intermediate thiourea **7** was introduced into the reaction with substituted benzaldehydes without additional purification, since the use of chromatography significantly reduced its yield (to 15–20%). The most characteristic signals in the ^1^H NMR spectra of the obtained 2-thiohydantoins **8** were a doublet of the -N–CH_2_– group with a *J* value of 1.5–3.0 Hz at 4.4–4.6 ppm, and a triplet of -C ≡CH with the same coupling constant at 3.1–3.4 ppm.

**Scheme 3:**
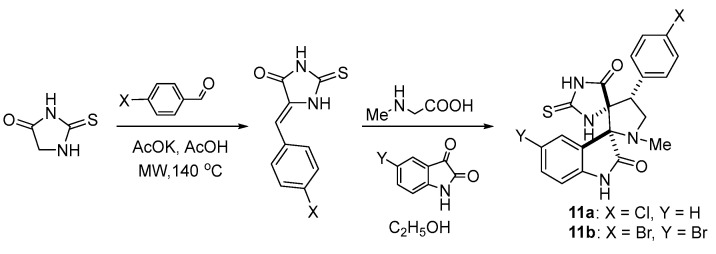
Synthesis of compounds **11a**,**b**.

The thiohydantoins **8** were then subjected to (3+2) cycloaddition with an azomethine ylide generated from isatins and sarcosine under standard conditions to afford dispiroindolinones **9a**–**c** (Scheme 3). The structures of all dispiroindolinones were established by NMR spectroscopy and high-resolution mass spectrometry. The characteristic signals of dispiroindolinones **9** in their ^1^H NMR spectra are pseudo-triplets of three protons of the pyrrolidine ring in the 3–4.5 ppm region, signals of the NH protons of the imidazolidine and indolinone rings in the downfield region, and signals of the propargyl group; the triplet of the−C ≡CH protons with a coupling constant of 2.4–2.5 Hz in the 3.1–3.4 ppm region may not be observed in some cases due to the overlap with the signal of water contained in DMSO-d6. The signal of the -N-CH_2_- group is slightly shifted upfield (4.1–4.3 ppm) compared to the similar signal in the starting 5-arylmethylidene-2-thiohydantoin and overlaps with the signal of one of the protons of the pyrrolidine ring.

**Figure 3:**
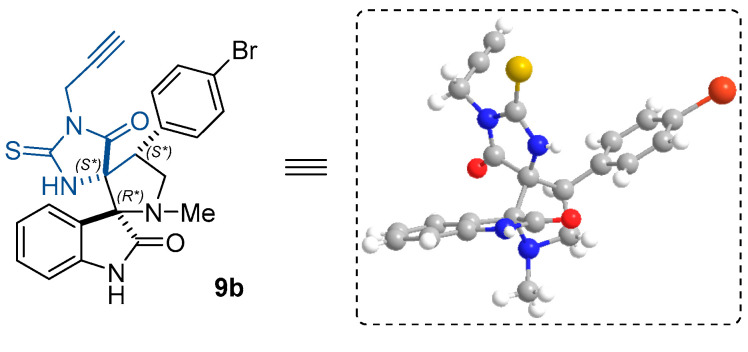
Molecular structure of **9b** (CCDC 2547584) according to X-ray diffraction data.

The structure of compound **9b** was further confirmed by X-ray crystallographic analysis (Figure 3), which established the relative configuration of the pyrrolidine ring stereocenters as (2′R*, 3′S*, 4′S*).

The nature and position of halogen substituents in compounds **6a**–**c** and **10a**–**c** did not significantly affect the yield of the target compounds. The halogen-substituted arylidene thiohydantoins **1** and 5-haloisatins were chosen to obtain adducts **6** and **10**, since spiro derivatives with similar substituents demonstrated the highest cytotoxicity among dispiro derivatives of the structural type **A** (Figure 2) [9,34,35,36,37].

For the biological evaluation, we synthesized dispiroindolinones **11a**,**b** (Scheme 3, [54]) that are similar to **6a** and **6b** but lack a glutarimide fragment. Compounds **11** were used as references to judge whether the glutarimide moiety affects cytotoxicity.

### 2.2. Biological Testing

Initially, we performed MTT assays for compounds **6a**–**c**, **10b**,**c**, and **11a**,**b** on the HCT116 cell line (wild-type p53). Table 1 shows that **6a**, **6b**, and **11b** caused an antiproliferative effect (IC_50_ 10–15 μM after 72 h of cell exposure); whereas compounds **6c**, **10b**, **10c**, and **11a** turned out to be less potent (IC_50_ > 22 μM). This result agrees with the results of [9,35,36], confirming that the halogen atom in the aryl substituent and the bromine in the isatin fragment of dispiroindolinone increase cytotoxicity.

**Figure 4:**
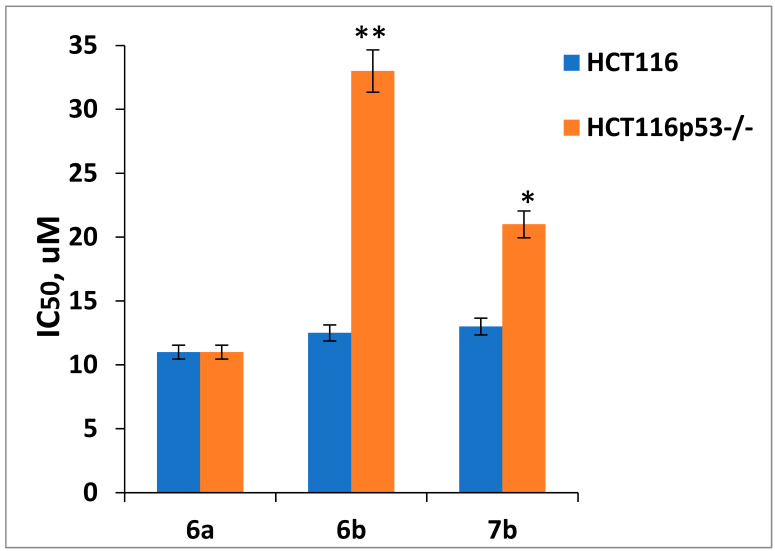
Cytotoxicity of selected new compounds for HCT116 cells (wild-type p53) and HCT116p53^−/−^ subline. Here, in Table 1 and in Figures S1 and S2: mean ± S.D. (MTT tests) after 72 h of cell exposure. Experiments were performed three times, each concentration tested in duplicate. * *p* < 0.05, ** *p* < 0.001 compared to the respective HCT116 group.

To clarify the role of p53 in the cellular response, these compounds were tested against the isogenic HCT116p53^−/−^ subline with deletion of both alleles of the *p53* gene (see Materials and Methods). As shown in Figure 4, two out of three derivatives were less cytotoxic in HCT116p53^−/−^ cells than in the wild-type counterparts.

To verify the difference in cytotoxicity of the studied compounds between wild-type p53 and isogenic p53-null cells, we tested **6a** and **6b** on the A549 lung cancer cell line and its p53 knockout subline obtained after CRISPR-Cas-9-assisted knockout. However, the difference in activity between the wild-type and knockout cells was insignificant (Supplementary Information). We attribute this discrepancy to the cell-type-specific role of p53 signaling in cell death. We focused on cytotoxicity as a major readout. One may suggest that even if **6a**,**b** were able to disrupt the p53-MDM2 interaction, other downstream mechanisms could hamper the pro-apoptotic activity of the stabilized p53. In other words, the biochemical effect of PROTAC-like agents may or may not be paralleled by the necessary endpoint, such as p53-dependent cell death. Finally, one may suggest that the linker between glutarimide and the spiro fragment is not long enough to coordinate CRBN and MDM2. Thus, more work is needed to optimize the structure of the conjugates, as well as to expand the screening panel and include a variety of cell types with different p53 status.

## 3. Materials and Methods

### 3.1. General

All solvents used were purified and dried using standard techniques. All starting reagents were purchased from commercial sources (Sigma-Aldrich, ABCR, AKSci, Burlington, VT, USA). Unless otherwise stated, all reactions were monitored by TLC analysis using silica plates with a fluorescent indicator (254 nm) and visualized with a UV lamp.

^1^H and ^13^C NMR spectra were recorded on a Bruker Avance-400 (Bruker Biospin, Karlsruhe, Germany) and Agilent 400-MR (Agilent Technologies, Santa Clara, CA, USA) spectrometers (400 MHz for ^1^H, 101 MHz for ^13^C) or on a Q.One Quantum I-600 spectrometer (Q.One Instruments Ltd./Wuhan Zhongke-Niujin Magnetic Resonance Technology Co., Ltd., China; 600 MHz for ^1^H, 151 MHz for ^13^C). All chemical shifts (δ) are reported in parts per million (ppm) with ^1^H and ^13^C NMR referenced to solvent signals [^1^H NMR: CDCl_3_ (7.27), DMSO-*d_6_* (2.50); ^13^C NMR: CDCl_3_ (77.16), DMSO-*d_6_* (39.52)]. Coupling constants (*J*) are reported in Hertz (Hz).

High-resolution mass spectra were obtained using electrospray ionization (ESI) in positive ion mode on a TripleTOF 5600+ quadrupole time-of-flight mass spectrometer (ABSciex, Concord, Canada) equipped with a DuoSpray ion source. The following MS parameters were applied: capillary voltage, 5.5 kV; nebulizing and curtain gas pressures, 15 psi and 25 psi, respectively; ambient ion source temperature; declustering, potential 20 V; *m*/*z* range 100–1200. Elemental compositions of the detected ions were determined based on accurate masses and isotope distributions using Formula Finder software (version 1.5.2, ABSciex, Concord, Canada). The maximum allowed deviation of the experimental vs. calculated molecular mass was 5 ppm. Experimental details and characteristic data for all synthesized compounds are given in the Supplementary Information.

IR spectra were recorded on an IR200 Fourier transform IR spectrometer (TermoNicolet, Madison, WI, USA) with a resolution of 4 cm^−1^.

For compound **9b**, the X-ray data were collected using a STOE diffractometer Pilatus100K detector, a focusing mirror, collimation Cu Kα (1.54086 Å) radiation, and a rotation method. STOE X-AREA software (https://www.stoe.com/products/xarea/, accessed on 8 April 2026) was used for cell refinement and data reduction. Data collection and image processing were performed with X-Area 1.67 (STOE & Cie GmbH, Darmstadt, Germany, 2013). Intensity data were scaled with LANA (part of X-Area) in order to minimize differences in intensities of symmetry-equivalent reflections (multi-scan method). The structures were solved and refined with the SHELXT51 program. The non-hydrogen atoms were refined using the anisotropic full-matrix least-square procedure. All hydrogen atoms were placed in calculated positions and allowed to ride on their parent atoms [C-H 0.93–0.97; Uiso 1.2 Ueq(parent atom)]. Molecular geometry calculations were performed with the SHELX program, and the molecular graphics were prepared using DIAMOND software (version 5.1.0). CCDC-2547584 contains the supplementary crystallographic and geometric data for the compound (R)-10a. These data can be obtained free of charge from The Cambridge Crystallographic Data Centre via www.ccdc.cam.ac.uk/data_request/cif, accessed on 26 March 2026.

### 3.2. Synthesis

3-Methylenepiperidine-2,6-dione **4** was synthesized according to [52,53] from chloroacetamide and ethyl acrylate. 3-(Azidomethyl)piperidine-2,6-dione **5** was synthesized according to [29]. The TBTA catalyst was synthesized as in [55,56]. The starting thiohydantoins were prepared using methods described in [57,58,59,60,61,62].

**General procedure for the synthesis of 2-thiohydantoins 1a**–**c**. The substituted benzaldehyde (1.1 eq.), glycine (1.1 eq.), and phenyl isothiocyanate (1 eq.) were dissolved in glacial acetic acid. The mixture was refluxed for 3–4 h, and the reaction was monitored by TLC (eluent, *n*-hexane/ethyl acetate 3:1). After completion of the reaction the mixture was poured into an excess of cold water, and the resulting precipitate was filtered, washed with cold water, and air-dried to obtain a yellow solid, followed by recrystallization from ethanol or glacial acetic acid or purification by column chromatography on silica gel (eluent, *n*-hexane/ethyl acetate 3:1, Rf ~ 0.35–0.4).

**(*Z*)-5-(4-chlorobenzylidene)-3-phenyl-2-thioxoimidazolidin-4-one (1a)**. From 0.618 g of 4-chlorobenzaldehyde (4.4 mmol, 1.1 eq.), 0.33 g of glycine (4.4 mmol, 1.1 eq.), and 0.541 g of phenyl isothiocyanate (4 mmol, 1 eq., 478 µL), 0.781 g of compound **1a** was obtained as a yellow solid (62% yield after chromatography). M.p. 165–167 °C (lit: M.p. 165–168 °C [48]). **^1^H NMR** (400 MHz, DMSO-d_6_) δ 12.66 (s, 1H), 7.89–7.81 (m, 2H), 7.56–7.41 (m, 5H), 7.42–7.35 (m, 2H), 6.68 (s, 1H). **HRMS** (ESI-TOF) *m*/*z*: [M+H]^+^ calculated for [C_16_H_11_ClN_2_OS+ H]^+^ 315.0359; found 315.0357.

**(*Z*)-5-(4-bromobenzylidene)-3-phenyl-2-thioxoimidazolidin-4-one (1b)**. From 0.814 g of 4-bromobenzaldehyde (4.4 mmol, 1.1 eq.), 0.33 g of glycine (4.4 mmol, 1.1 eq.), and 0.541 g of phenyl isothiocyanate (4 mmol, 1 eq., 478 µL), after recrystallization from glacial acetic acid, 0.687 g of compound **1b** was obtained as a yellow solid (48% yield). M.p. 158–160 °C. **^1^H NMR** (400 MHz, DMSO-d_6_) δ 12.65 (s, 1H), 7.82–7.70 (m, 2H), 7.69–7.61 (m, 2H), 7.60–7.42 (m, 3H), 7.41–7.36 (m, 2H), 6.66 (s, 1H). **HRMS** (ESI-TOF) *m*/*z*: [M+H]^+^ calculated for [C_16_H_11_BrN_2_OS+H]^+^ 358.9854; found 358.9862.

**(*Z*)-5-benzylidene-3-phenyl-2-thioxoimidazolidin-4-one (1c)**. From 0.467 g of benzaldehyde (4.4 mmol, 1.1 eq.), 0.33 g of glycine (4.4 mmol, 1.1 eq.), and 0.541 g of phenyl isothiocyanate (4 mmol, 1 eq., 478 µL), after recrystallization from ethanol, 0.785 g of compound **1c** was obtained as a yellow solid (70% yield). M.p. 208–209 °C. **^1^H NMR** (400 MHz, DMSO-d_6_) δ 12.63 (s, 1H), 7.87–7.79 (m, 2H), 7.57–7.36 (m, 8H), 6.70 (s, 1H). **HRMS** (ESI-TOF) *m*/*z*: [M+H]^+^ calculated for [C_16_H_12_N_2_OS+ H]^+^ 281.0749; found 281.0756.

**General procedure for the synthesis of S-propargyl-2-thiohydantoins 2a–c**. 2-Thiohydantoin (1 eq.) was dissolved in 95% ethanol (10 mL/1 mmol), after which potassium hydroxide (1.2 eq.) was added. After the solution changed color, propargyl bromide (1.2 eq.) was added dropwise, and the mixture was stirred at room temperature overnight. An excess of cold water was added. The precipitate was filtered and washed with 10% aqueous sodium hydroxide, water, and cold petroleum ether. The resulting residue was purified by column chromatography (eluent petroleum ether/ethyl acetate gradient from 4:1 to 1:1) to yield a bright yellow solid.

**(*Z*)-5-(4-chlorobenzylidene)-3-phenyl-2-(prop-2-yn-1-ylthio)-3,5-dihydro-4H-imidazol-4-one (2a)**. From 0.315 g of **1a** (1 mmol, 1 eq.), 0.067 g of potassium hydroxide (1.2 mmol, 1.2 eq.), and 0.119 g of propargyl bromide (1.2 mmol, 1.2 eq.), after column chromatography, 0.299 g of compound **2a** was obtained as a bright yellow solid (84% yield). **^1^H NMR** (400 MHz, DMSO-d_6_) δ 8.37–8.29 (m, 2H), 7.59–7.49 (m, 5H), 7.45–7.39 (m, 2H), 7.03 (s, 1H), 4.19 (d, *J* = 2.6 Hz, 2H), 3.32 (t, *J* = 2.6 Hz, 1H). **^13^C NMR** (101 MHz, DMSO-d_6_) δ 167.9, 163.9, 138.1, 134.7, 133.5 (2C), 133.1, 132.1, 129.7 (2C), 129.6, 128.9 (2C), 127.7 (2C), 122.2, 78.9, 74.6, 19.1. **HRMS** (ESI-TOF) *m*/*z*: [M+H]^+^ calculated for [C_19_H_13_ClN_2_OS + Na]^+^ 375.0329; found 375.0335.

**(*Z*)-5-(4-bromobenzylidene)-3-phenyl-2-(prop-2-yn-1-ylthio)-3,5-dihydro-4H-imidazol-4-one (2b)**. From 0.359 g of **1b** (1 mmol, 1 eq.), 0.067 g of potassium hydroxide (1.2 mmol, 1.2 eq.), and 0.119 g of propargyl bromide (1.2 mmol, 1.2 eq.), after column chromatography, 0.32 g of compound **2b** was obtained as a bright yellow solid (80% yield).

**^1^H NMR** (400 MHz, DMSO-d_6_) δ 8.24 (d, *J* = 8.3 Hz, 2H), 7.67 (d, *J* = 8.3 Hz, 2H), 7.61–7.16 (m, 5H), 7.00 (s, 1H), 4.18 (d, *J* = 2.6 Hz, 2H), 3.33 (t, *J* = 2.6 Hz, 1H). **^13^C NMR** (101 MHz, DMSO) δ 167.9, 163.9, 138.2, 133.7 (2C), 133.4, 131.9 (2C), 129.7 (2C), 129.6, 127.7 (2C), 123.7, 122.2, 78.9, 74.6, 19.2. **HRMS** (ESI-TOF) *m*/*z*: [M+H]^+^ calculated for [C_19_H_13_BrN_2_OS + Na]^+^ 397.0005; found 397.0012.

**(*Z*)-5-benzylidene-3-phenyl-2-(prop-2-yn-1-ylthio)-3,5-dihydro-4H-imidazol-4-one (2c)**. From 0.28 g of **1c** (1 mmol, 1 eq.), 0.067 g of potassium hydroxide (1.2 mmol, 1.2 eq.), and 0.119 g of propargyl bromide (1.2 mmol, 1.2 eq.), after column chromatography, 0.255 g of compound **2c** was obtained as a bright yellow solid (84% yield). **^1^H NMR** (400 MHz, DMSO-d_6_) 8.34–8.27 (m, 2H), 7.62–7.40 (m, 8H), 7.03 (s, 1H), 4.23–4.17 (m, 2H), 3.33–3.29 (m, 1H, overlapped with H_2_O signal from DMSO-d_6_). **^13^C NMR** (101 MHz, DMSO-*d*_6_) δ 167.9, 163.2, 137.6, 134.0, 132.1, 131.9 (2C), 130.1, 129.6 (2C), 129.4, 128.8 (2C), 127.6 (2C), 123.7, 78.9, 74.5, 19.0. **HRMS** (ESI-TOF) *m*/*z*: [M+H]^+^ calculated for [C_19_H_14_N_2_OS + H]^+^ 319.0905; found 319.0901.

**General procedure for the synthesis of dispiroindolinones 3a**–**c**. To a boiling solution of 2-(prop-2-yn-1-ylthio)hydantoins **2a**–**2e** (1 eq.) and sarcosine (3 eq.) in 95% ethanol, 5-bromoisatin (2 eq.) was added in two portions 15 min apart; the resulting mixture was boiled for 6–8 h (TLC monitoring). After completion of the reaction and cooling the solution, a 3-fold volume of cold water was added, and the obtained precipitate was filtered off. The residue was purified by column chromatography on silica gel (petroleum ether/ethyl acetate 3:1) to yield a white solid.

(2′S*,4R*,4′R*)-5″-bromo-4′-(4-chlorophenyl)-1′-methyl-1-phenyl-2-(prop-2-yn-1-ylthio)dispiro[imidazole-4,3′-pyrrolidine-2′,3″-indoline]-2″,5(1H)-dione (3a). From 176 mg of **2a** (0.5 mmol, 1 eq.), 134 mg of sarcosine (1.5 mmol, 3 eq.), and 226 mg of 5-bromoisatin (1 mmol, 2 eq.), after column chromatography, 142 mg of compound **3a** was obtained as a white solid (47% yield). **^1^H NMR** (400 MHz, DMSO-d_6_) δ 10.43 (s, 1H), 7.61–7.56 (m, 2H), 7.48 (dd, *J* = 8.3, 2.2 Hz, 1H), 7.45–7.39 (m, 3H), 7.36–7.31 (m, 2H), 7.29 (d, *J* = 2.2 Hz, 1H), 6.78 (d, *J* = 8.3 Hz, 1H), 6.71–6.66 (m, 2H), 4.11–4.03 (m, 2H), 3.66 (dd, *J* = 16.3, 2.6 Hz, 1H), 3.62 (dd, *J* = 16.3, 2.6 Hz, 1H), 3.57–3.48 (m, 1H), 3.04 (t, *J* = 2.6 Hz, 1H), 2.19 (s, 3H). **^13^C NMR** (101 MHz, DMSO) δ 180.5, 175.5, 160.0, 143.1, 135.7, 132.6, 132.3 (2C), 131.6, 131.1, 129.65, 129.62 (2C), 128.7, 127.6 (2C), 127.0 (2C), 126.6, 112.9, 111.4, 83.8, 78.1, 77.0, 74.2, 60.0, 50.9, 34.4, 18.3. **HRMS** (ESI-TOF) *m*/*z*: [M+H]^+^ calculated for [C_29_H_22_BrClN_4_O_2_S + Na]^+^ 627.0233; found 627.0234.

(2′S*,4R*,4′R*)-5″-bromo-4′-(4-bromophenyl)-1′-methyl-1-phenyl-2-(prop-2-yn-1-ylthio)dispiro[imidazole-4,3′-pyrrolidine-2′,3″-indoline]-2″,5(1H)-dione (3b). From 198 mg of **2b** (0.5 mmol, 1 eq.), 134 mg of sarcosine (1.5 mmol, 3 eq.), and 226 mg of 5-bromoisatin (1 mmol, 2 eq.), after column chromatography, 166 mg of compound **3b** was obtained as a white solid (51% yield). For the compound **3b,** 2D NMR spectra (HSQC, HMBC, HOMODEC) were recorded, and a complete signal correlation was carried out. **^1^HNMR**(600 MHz, DMSO-d_6_) δ 10.43 (s, 1H, NH), 7.55–7.51 (m, 2H, H^27^, H^31^), 7.48 (dd, 1H, ^3^*J* = 8.3 Hz, ^4^*J* = 2.1 Hz, H^2^), 7.49–7.45 (m, 2H, H^28^, H^30^), 7.45–7.38 (m, 3H, H^39^, H^40^, H^41^), 7.28 (d, ^4^*J* = 2.1 Hz, 1H, H^6^), 6.78 (d, ^3^*J* = 8.3 Hz, 1H, H^3^), 6.71–6.66 (2H, H^38^, H^42^), 4.10–4.02 (m, 2H, H^35^, H^12^), 3.67 (dd, ^2^*J* = 16.1 Hz, ^4^*J* = 2.7 Hz, 1H, H^33^), 3.62 (dd, ^2^*J* = 16.1 Hz, ^4^*J* = 2.7 Hz, 1H, H^34^), 3.56–3.48 (m, 1H, H^36^), 3.03 (t, ^4^*J* = 2.7 Hz, 1H, H^25^), 2.19 (s, 3H, CH_3_)**. ^13^C NMR** (151 MHz, DMSO) δ 180.4 (C^14^), 175.4 (C^8^), 160.0 (C^16^), 143.1 (C^4^), 136.2(C^26^), 132.7 (2C, C^27^, C^31^), 132.6 (C^2^), 131.1 (C^37^), 130.5 (2C, C^28^, C^30^), 129.63 (C^40^), 129.61 (2C, C^39^, C^41^), 128.7 (C^6^), 127.0 (2C, C^38^, C^42^), 126.6 (C^5^), 120.2 (C^29^), 112.9 (C^1^), 111.4 (C^3^), 83.8 (C^13^), 78.1 (C^24^), 77.0 (C^9^), 74.2 (C^25^), 60.0 (C^11^), 51.0 (C^12^), 34.4 (C^21^), 18.3 (C^23^). **HRMS** (ESI-TOF) *m*/*z*: [M+H]^+^ calculated for [C_29_H_22_Br_2_N_4_O_2_S + Na]^+^ 670.9722; found 670.9728.


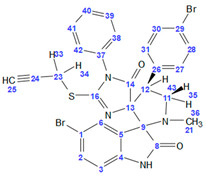


(2′S*,4R*,4′R*)-5″-bromo-1′-methyl-1,4′-diphenyl-2-(prop-2-yn-1-ylthio)dispiro[imidazole-4,3′-pyrrolidine-2′,3″-indoline]-2″,5(1H)-dione (3c). From 318 mg of **2c** (1 mmol, 1 eq.), 267 mg of sarcosine (3 mmol, 3 eq.), and 452 mg of 5-bromoisatin (2 mmol, 2 eq.), after column chromatography, 257 mg of compound **3c** was obtained as a white solid (46% yield). **^1^H NMR** (400 MHz, DMSO-d_6_) δ 10.40 (s, 1H), 7.56–7.51 (m, 2H), 7.46 (dd, *J* = 8.3, 2.0 Hz, 1H), 7.45–7.36 (m, 3H), 7.33–7.17 (m, 4H), 6.77 (d, *J* = 8.3, 1H), 6.70–6.65 (m, 2H), 4.09–4.01 (m, 2H), 3.64 (dd, *J* = 16.2, 2.7 Hz, 1H), 3.59 (dd, *J* = 16.2, 2.7 Hz, 1H), 3.54–3.46 (m, 1H), 3.02 (t, *J* = 2.7 Hz, 1H), 2.20 (s, 3H). **^13^C NMR** (101 MHz, DMSO) δ 180.6, 175.4, 159.5, 143.1, 136.6, 132.5 (2C), 131.2, 130.4 (2C), 129.62 (2C), 129.60, 128.7, 127.6, 126.9 (2C), 126.8, 126.8, 112.9, 111.3, 84.1, 77.9, 77.0, 74.4, 59.9, 51.9, 34.5, 18.3. **HRMS** (ESI-TOF) *m*/*z*: [M+H]^+^ calculated for [C_29_H_23_BrN_4_O_2_S + H]^+^ 593.0623; found 593.0625.

**General procedure for the synthesis of conjugates 6a**–**c**. To a solution of the terminal alkyne **3a**–**3e** (1 equiv.) and TBTA (0.3 equiv.) in CH_2_Cl_2_ (5 mL/0.1 mmol of alkyne) under argon, a solution of CuSO_4_*5H_2_O (0.3 equiv.) in 200 μL of dH_2_O was added, followed by a solution of sodium ascorbate (0.6 equiv.) in 200 μL of dH_2_O. The reaction mixture was stirred for 30 min, then a solution of the N_3_-glutarimide (1 equiv.) in CH_2_Cl_2_ (2.5 mL/0.1 mmol of azide) was added dropwise to the resulting dark red solution. The mixture was stirred overnight at room temperature under argon. Then, the solvent was evaporated under reduced pressure, and the residue was purified by column chromatography (eluent—CH_2_Cl_2_/CH_3_OH 99:1 → 30:1) to obtain a white solid.

(2′S*,4R*,4′R*)-5″-bromo-4′-(4-chlorophenyl)-2-(((1-((2,6-dioxopiperidin-3-yl)methyl)-1H-1,2,3-triazol-4-yl)methyl)thio)-1′-methyl-1-phenyldispiro[imidazole-4,3′-pyrrolidine-2′,3″-indoline]-2″,5(1H)-dione (6a). From 61 mg of **3a** (0.1 mmol, 1 eq.), 16 mg of TBTA (0.03 mmol, 0.3 eq.), 7.5 mg of CuSO_4_*5H_2_O (0.03 mmol, 0.3 eq.) in 200 μL of deionized water, 12 mg of NaAsc (0.06 mmol, 0.6 eq.) in 200 μL of deionized water and 17 mg of 5 (0.1 mmol, 1 eq.) 55 mg of compound **6a** was obtained as a white solid (71% yield). Compound **6c** was obtained as a mixture of two diastereomers (A:B = 0.50:0.50). **^1^H NMR** (400 MHz, DMSO-d6) δ 10.87 (s, 1H^A^ +1H^B^), 10.53/10.49 (s. 1H^A^ +1H^B^), 7.711/7.706 (s, 1H^A^ +1H^B^), 7.58–7.53 (m, 2H^A^ +2H^B^), 7.53–7.47 (m, 3H^A^ +3H^B^), 7.44–7.27 (m, 4H^A^ +4H^B^), 6.820/6.817 (d, *J* = 8.3 Hz, 1H^A^ +1H^B^), 6.71–6.65 (m, 2H^A^ +2H^B^), 4.793/4.786 (dd, *J* = 14.0, 4.9 Hz, 1H^A^ +1H^B^), 4.563/4.527 (dd, *J* = 14.0, 7.9 Hz, 1H^A^ +1H^B^), 4.13–4.07 (m, 2H^A^ +2H^B^), 4.073/4.066 (d, *J* = 14.1, Hz, 1H^A^ +1H^B^), 4.020/4.016 (d, *J* = 14.1, Hz, 1H^A^ +1H^B^), 3.58–3.50 (m, 1H^A^ +1H^B^), 3.21–3.09 (m, 1H^A^ +1H^B^), 2.60–2.40 (m, 2H^A^ +2HB, overlapped with DMSO signal), 2.21 (s, 3H^A^ +3H^B^), 1.69–1.60 (m, 2H^A^ +2H^B^). **^13^C NMR** (101 MHz, DMSO-d_6_) δ (A/B): 180.2/180.2, 175.9/175.9, 173.1/173.1, 173.1/173.1, 161.3/161.3, 143.0/143.0, 141.5/141.3, 136.4/136.3, 132.7/132/7 (2C), 132.6/132.6, 131.3/131.3, 130.6/132.6 (2C), 129.6/129.6, 129.6/129.6 (2C), 128.8/128.8, 128.0/128/0, 127.1/127.1 (2C), 126.8/126.8, 124.6/124.4, 113.1/113.1, 111.5/111.5, 83.9/83.9, 77.1/77.0, 59.9/59.8, 50.9/50.8, 49.2/49.1, 41.2/41.2, 34.5/34.5, 30.9/30.9, 24.9/24.9, 21.3/21.2. **IR** (cm^−1^): 3400–2800 br., 1704 (C=O). **HRMS** (ESI-TOF) *m*/*z*: [M+H]^+^ calculated for [C_35_H_30_BrClN_8_O_4_S + Na]^+^ 795.0875; found 795.0881.

(2′S*,4R*,4′R*)-5″-bromo-4′-(4-bromophenyl)-2-(((1-((2,6-dioxopiperidin-3-yl)methyl)-1H-1,2,3-triazol-4-yl)methyl)thio)-1′-methyl-1-phenyldispiro[imidazole-4,3′-pyrrolidine-2′,3″-indoline]-2″,5(1H)-dione (6b). From 65 mg of **3b** (0.1 mmol, 1 eq.), 16 mg of TBTA (0.03 mmol, 0.3 eq.), 7.5 mg of CuSO_4_*5H_2_O (0.03 mmol, 0.3 eq.) in 200 μL of deionized water, 12 mg of NaAsc (0.06 mmol, 0.6 eq.) in 200 μL of deionized water, and 17 mg of 5 (0.1 mmol, 1 eq.) 54 mg of compound **6b** was obtained as a white solid (66% yield). Compound **6b** was obtained as a mixture of two diastereomers (A:B = 1:1). **^1^H NMR** (400 MHz, DMSO-d6) δ 10.87 (s, 1H^A^ + 1H^B^), 10.52/10.49 (s, 1H^A^ +1H^B^), 7.90/7.69 (s, 1H^A^ +1H^B^), 7.64–7.58 (m, 2H^A^ +2H^B^), 7.495/7.492 (dd, *J* = 8.3, 2.1Hz, 1H^A^ +1H^B^), 7.45–7.26 (m, 6H^A^ +6H^B^), 6.816/6.814 (d, *J* = 8.3 Hz, 1H^A^ +1H^B^), 6.71–6.66 (m, 2H^A^ +2H^B^), 4.772/4.778 (dd, *J* = 14.0, 4.9 Hz, 1H^A^ +1H^B^), 4.539/4.520 (dd, *J* = 14.1, 7.9 Hz, 1H^A^ +1H^B^), 4.14–4.06 (m, 2H^A^ +2H^B^), 4.069/4.061 (m, 1H^A^ +1H^B^), 4.014/4.007 (m, 2H^A^ +2H^B^), 3.58–3.50 (m, 1H^A^ +1H^B^), 3.19–3.09 (m, 1H^A^ +1H^B^), 2.62–2.39 (m, 2H^A^ +2H^B^, overlapped with DMSO signal), 2.20 (s, 3H^A^ +31H^B^), 1.59–1.69 (m. 2H^A^ +2H^B^). **^13^C NMR** (101 MHz, DMSO-d_6_) δ (A/B) 180.3/180.2, 175.9/175.9, 173.1/173.1, 173.1/173.1, 161.3/161.3, 143.0/143.0, 141.5/1415, 135.9/135.9, 132.6/132.6, 131.3/131.3 (2C), 131.6/131.6, 129.5/129.5, 129.6/129.6 (2C), 128.8/128.8, 128.1/128.1, 127.6/127.6 (2C), 127.1/127.1 (2C), 126.8/126.8, 124.6/124.4, 113.1/113.1, 111.5/11.5, 83.9/83.9, 77.1/77.0, 59.9/59.9, 50.8/50.8, 49.1/49.0, 41.2/41.2, 34.5/34.5, 30.9/30.9, 24.9/24.9, 21.2/21.2. **IR** (cm^−1^): 3400–2800 br., 1702 (C=O). **HRMS** (ESI-TOF) *m*/*z*: [M+H]^+^ calculated for [C_35_H_30_Br_2_N_8_O_4_S + Na]^+^ 839.0370; found 839.0375.

(2′S*,4R*,4′R*)-5″-bromo-2-(((1-((2,6-dioxopiperidin-3-yl)methyl)-1H-1,2,3-triazol-4-yl)methyl)thio)-1′-methyl-1,4′-diphenyldispiro[imidazole-4,3′-pyrrolidine-2′,3″-indoline]-2″,5(1H)-dione (6c). From 112 mg of **3c** (0.2 mmol, 1 eq.), 32 mg of TBTA (0.06 mmol, 0.3 eq.), 15 mg of CuSO_4_*5H_2_O (0.06 mmol, 0.3 eq.) in 200 μL of deionized water, 24 mg of NaAsc (0.12mmol, 0.6 eq.) in 200 μL of deionized water, and 34 mg of 5 (0.2 mmol, 1 eq.) 94 mg of compound **6c** was obtained as a white solid (64% yield). Compound **6c** was obtained as a mixture of two diastereomers (A:B = 0.55:0.45). For the compound **6c** 2D NMR spectra (HSQC, HMBC, COSY) were recorded, and a complete signal correlation was carried out. **^1^H NMR** (600 MHz, DMSO-d_6_) δ Isomer A: 10.886 (s, 1H, H^40^), 10.476 (s, 1H, H^7^), 7.59–7.56 (m, 2H, H^21^, H^25^), 7.514 (s, 1H, H^31^), 7.483 (dd, ^3^*J* = 8.3 Hz, ^4^*J* = 2.2 Hz, H^2^), 7.44–7.38 (m, 3H, H^47^,H^48^, H^49^), 7.35–7.30 (m, 3H, H^6^, H^22^, H^24^), 7.25–7.29 (m, 1H, H^23^), 6.812 (d, ^3^*J* = 8.3 Hz, 1H, H^3^), 6.69–6.66 (m, 2H, H^46^, H^50^), 4.752 (dd, ^2^*J* = 14.0 Hz, ^3^*J* = 5.2 Hz, 1H, H^54^), 4.496 (dd, ^2^*J* = 14.0 Hz, ^3^*J* = 8.0 Hz, 1H, H^55^), 4.206 (dd, ^2^*J* = 8.4, ^3^*J* = 10.0 Hz, 1H, H^51^), 4.097 (dd, ^2^*J* = 7.8 Hz, ^3^*J* = 10.0 Hz, 1H, H^52^), 4.029 (d, ^2^*J* = 14.1 Hz, 1H, H^56^), 3.955 (d, ^2^*J* = 14.1 Hz, 1H, H^57^), 3.536 (dd, ^2^*J* = 8.4, ^3^*J* = 7.8 Hz, 1H, H^53^), 3.15–3.08 (m, 1H, H^36^), 2.56–2.51 (m, 1H, H^38′^), 2.48–2.44 (m, 1H, H^38^), 2.219 (s, 3H, CH_3_), 1.60–1.68 (m, 2H, H^37^, H^37′^); Isomer B: 10.878 (s, 1H, H^40^), 10.507 (s, 1H, H^7^), 7.59–7.56 (m, 2H, H^21^, H^25^), 7.563 (s, 1H, H^31^), 7.485 (dd, ^3^*J* = 8.3 Hz, ^4^*J* = 2.2 Hz, H^2^), 7.44–7.38 (m, 3H, H^47^, H^48^, H^49^), 7.35–7.30 (m, 3H, H^6^, H^22^, H^24^), 7.24–7.27 (m, 1H, H^23^), 6.809 (d, ^3^*J* = 8.3 Hz, 1H, H^3^), 6.69–6.66 (m, 2H, H^46^, H^50^), 4.76 (dd, ^2^*J* = 14.0 Hz, ^3^*J* = 5.2 Hz, 1H, H^54^), 4.525 (dd, ^2^*J* = 14.0 Hz, ^3^*J* = 8.0 Hz, 1H, H^55^), 4.202 (dd, ^2^*J* = 8.4, ^3^*J* = 10.0 Hz, 1H, H^51^), 4.093 (dd, ^2^*J* = 7.8 Hz, ^3^*J* = 10.0 Hz, 1H, H^52^), 4.024 (d, ^2^*J* = 14.1 Hz, 1H, H^56^), 3.965 (d, ^2^*J* = 14.1 Hz, 1H, H^57^), 3.534 (dd, ^2^*J* = 8.4, ^3^*J* = 7.8 Hz, 1H, H^53^), 3.15–3.08 (m, 1H, H^36^), 2.59–2.54 (m, 1H, H^38′^), 2.41–2.45 (m, 1H, H^38^), 2.221 (s, 3H, CH_3_), 1.60–1.68 (m, 2H, H^37^, H^37′^). **^13^C NMR** (151 MHz, DMSO-d_6_) δ Isomer A: 180.4 (C^14^), 175.9 (C^8^), 173.1 (2C, C^39^, C^41^), 161.0 (C^16^), 143.0 (C^4^), 141.8 (C^30^), 136.9 (C^20^), 132.5 (C^2^), 131.3 (C^45^), 130.3 (2C, C^21^, C^25^), 129.6 (2C, C^47^, C^49^), 129.6 (C^48^), 128.8 (C^6^), 127.8 (2C, C^22^, C^24^), 127.1 (2C, C^46^, C^50^), 127.0 (C^5^), 126.9 (C^23^), 124.4 (C^31^), 113.0 (C^1^), 111.5 (C^3^), 84.3 (C^13^), 77.0 (C^9^), 59.5 (C^11^), 51.7 (C^12^), 49.1 (C^35^), 41.2 (C^36^), 34.5 (C^44^), 30.8 (C^38^), 24.8 (C^29^), 21.3 (C^37^); Isomer B: 180.4 (C^14^), 175.9 (C^8^), 173.1 (2C, C^39^, C^41^), 161.0 (C^16^), 143.0 (C^4^), 141.8 (C^30^), 136.8 (C^20^), 132.5 (C^2^), 131.3 (C^45^), 130.3 (2C, C^21^, C^25^), 129.6 (2C, C^47^, C^49^), 129.5 (C^48^), 128.9 (C^6^), 127.8 (2C, C^22^, C^24^), 127.0 (2C, C^46^, C^50^), 127.0 (C^5^), 126.9 (C^23^), 124.4 (C^31^), 113.0 (C^1^), 111.4 (C^3^), 84.3 (C^13^), 77.0 (C^9^), 59.5 (C^11^), 51.8 (C^12^), 49.1 (C^35^), 41.22 (C^36^), 34.5 (C^44^), 30.9 (C^38^), 24.8 (C^29^), 21.2 (C^37^). **IR** (cm^−1^): 3400–2800 br., 1703 (C=O). **HRMS** (ESI-TOF) *m*/*z*: [M+H]^+^ calculated for [C_35_H_31_BrN_8_O_4_S + Na]^+^ 761.1265; found 761.1273.


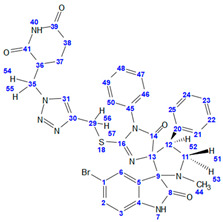


**General procedure for the synthesis of 2-thiohydantoins 8a,8b**. Propargylamine (0.05 mol, 1 equiv., 2.76 g, 3.2 mL) was dissolved in 100 mL of diethyl ether, after which 7.6 g of ethyl isothiocyanatoacetate (0.0525 mmol, 1.05 equiv., 7.64 mL) was added dropwise over 15 min, then the reaction mixture was stirred for 3 h at room temperature (TLC monitoring). After completion of the reaction, the solvent was evaporated, yielding 10 g of the thiourea **7** as a brown viscous oil, which was used further without further purification.

Thiourea **7** (1 eq.) was dissolved in 95% ethanol (15 mL/1 mmol), after which benzaldehyde (1.1 eq.) and potassium hydroxide (1 eq.) were added, resulting in a dark red solution. The reaction mixture was stirred for 3 h at room temperature (TLC monitoring), after which 1 M HCl solution was added to pH ~4–5, and the mixture was diluted with excess cold water, resulting in the formation of a yellow precipitate. The precipitate was filtered, then washed with 1M HCl solution, cold water, 95% ethanol, and a small amount of cold diethyl ether, and air-dried. The solid residue was purified by column chromatography (eluent—n-hexane/ethyl acetate 4:1).

**(*Z*)-5-(4-chlorobenzylidene)-3-(prop-2-yn-1-yl)-2-thioxoimidazolidin-4-one (8a)**. From 1 g of **7** (5 mmol, 1 eq.), 0.773 g of 4-chlorobenzaldehyde (5.5 mmol, 1.1 eq.), and 0.28 g of KOH (5 mmol, 1 eq.), 1.175 g of compound **8a** was obtained as a yellow solid (85% yield). **^1^H NMR** (400 MHz, DMSO-d_6_) δ 12.61 (s, 1H, NH), 7.80 (d, *J* = 8.6 Hz, 2H, Ar), 7.50 (d, *J* = 8.6 Hz, 2H, Ar), 6.69 (s, 1H, -C=CH-), 4.58 (d, *J* = 2.5 Hz, 2H, -CH_2_-), 3.27 (t, *J* = 2.5 Hz, 1H, -C≡CH). **HRMS** (ESI-TOF) *m*/*z*: [M+H]^+^ calculated for [C_13_H_9_ClN_2_OS + 2Na–H]^+^ 320.9841; found 320.9845.

**(*Z*)-5-(4-bromobenzylidene)-3-(prop-2-yn-1-yl)-2-thioxoimidazolidin-4-one (5b)**. From 1 g of **7** (5 mmol, 1 eq.), 1.018 g of 4-chlorobenzaldehyde (5.5 mmol, 1.1 eq.), and 0.28 g of KOH (5 mmol, 1 eq.), 1.152 g of compound **8b** was obtained as a yellow solid (72% yield). **^1^H NMR** (400 MHz, DMSO-d_6_) δ 12.60 (s, 1H, NH), 7.71 (d, *J* = 8.7 Hz, 2H, Ar), 7.62 (d, *J* = 8.7 Hz, 3H, Ar), 6.65 (s, 1H, -C=CH-), 4.57 (d, *J* = 2.5 Hz, 2H, -CH_2_-), 3.26 (t, *J* = 2.5 Hz, 1H, -C≡CH). **HRMS** (ESI-TOF) *m*/*z*: [M+H]^+^ calculated for [C_13_H_9_BrN_2_OS + 2Na − H]^+^ 364.9331; found 364.9334.

**General procedure for the synthesis of dispiroindolinones 9a–c**. To a boiling solution of 2-thiohydantoin **8a** or **8b** (1 eq.) and sarcosine (3 eq.) in 95% ethanol, isatin or 5-bromoisatin (2 eq.) was added in 2 portions (15 min between), and the resulting mixture was boiled for 6–8 h (TLC monitoring). After completion of the reaction and cooling of the solution, three-fold volume of cold water was added, and the obtained precipitate was filtered off. The obtained residue was purified by column chromatography on silica gel (petroleum ether/ethyl acetate, 2:1) to yield a white solid.

(2′R*,4S*,4′S*)-5″-bromo-4′-(4-chlorophenyl)-1′-methyl-1-(prop-2-yn-1-yl)-2-thioxodispiro[imidazolidine-4,3′-pyrrolidine-2′,3″-indoline]-2″,5-dione (9a). From 0.277 г (1 mmol, 1 eq.) of **8a**, 0.267 г (3 mmol, 3 eq.) of sarcosine, and 0.452 г (2 mmol, 2 eq.) of 5-bromoisatin, 0.281 g of compound **9a** was obtained as a white solid (53% yield). **^1^H NMR** (400 MHz, DMSO-d_6_) δ 10.71 (s, 1H, NH(1″)), 10.41 (s, 1H, NH(3)), 7.56–7.51 (m, 1H, Ar), 7.45–7.37 (m, 1H, Ar), 7.36–7.31 (m, 4H, Ar), 6.74 (d, *J* = 8.5 Hz, 1H, Ar), 4.29–4.20 (m, 3H, -NCH_2_- + pyrrolidine), 3.86 (t, *J* = 9.5 Hz, 1H, pyrrolidine), 3.44 (t, *J* = 8.8 Hz, 1H, pyrrolidine), 2.98 (t, *J* = 2.5 Hz, 1H, -C≡CH), 2.11 (s, 3H, NCH_3_). **^13^C NMR** (101 MHz, DMSO) δ 179.5, 174.1, 171.4, 141.8, 133.5, 132.7, 132.3, 131.3 (2C), 129.8, 128.3 (2C), 126.2, 113.7, 111.7, 77.1, 76.9, 76.1, 73.5, 55.9, 50.1, 34.7, 29.8. **HRMS** (ESI-TOF) *m*/*z*: [M+H]^+^ calculated for [C_23_H_18_BrClN_4_O_2_S + Na]^+^ 550.9915; found 550.9914.

(2′R*,4S*,4′S*)-4′-(4-bromophenyl)-1′-methyl-1-(prop-2-yn-1-yl)-2-thioxodispiro[imidazolidine-4,3′-pyrrolidine-2′,3″-indoline]-2″,5-dione (9b). From 0.321 г (1 mmol, 1 eq.) of **8b**, 0.267 г (3 mmol, 3 eq.) of sarcosine, and 0.294 г (2 mmol, 2 eq.) of isatin, 0.183 g of compound **9b** was obtained as a white solid (37% yield). **^1^H NMR** (400 MHz, DMSO-d_6_) δ 10.71 (s, 1H, NH(1″)), 10.41 (s, 1H, NH(3)), 7.53 (d, *J* = 2.0 Hz, 1H, Ar), 7.48 (d, *J* = 8.4 Hz, 2H, Ar), 7.42 (dd, *J* = 8.3, 2.0 Hz, 1H, Ar), 7.27 (d, *J* = 8.4 Hz, 2H, Ar), 6.74 (d, *J* = 8.3 Hz, 1H, Ar), 4.27–4.18 (m, 3H, -NCH_2_- + pyrrolidine), 3.85 (t, *J* = 9.4 Hz, 1H, pyrrolidine), 3.48–3.41 (m, 1H, pyrrolidine), 2.98 (t, *J* = 2.4 Hz, 1H, -C≡CH), 2.11 (s, 3H, NCH_3_). **^13^C NMR** (101 MHz, DMSO) δ 179.6, 174.6, 171.7, 142.6, 134.2, 131.7 (2C), 131.3 (2C), 129.9, 127.2, 123.6, 121.8, 120.9, 109.8, 77.1, 77.0, 76.0, 73.7, 56.0, 50.2, 34.6, 29.8. **HRMS** (ESI-TOF) *m*/*z*: [M+H]^+^ calculated for [C_23_H_19_BrN_4_O_2_S + Na]^+^ 517.0310; found 517.0318.

(2′R*,4S*,4′S*)-5″-bromo-4′-(4-bromophenyl)-1′-methyl-1-(prop-2-yn-1-yl)-2-thioxodispiro[imidazolidine-4,3′-pyrrolidine-2′,3″-indoline]-2″,5-dione (9c). From 0.321 г (1 mmol, 1 eq.) of **8b**, 0.267 г (3 mmol, 3 eq.) of sarcosine, and 0.452 г (2 mmol, 2 eq.) of 5-bromoisatin, 0.287 g of compound **9c** was obtained as a white solid (50% yield). **^1^H NMR** (400 MHz, DMSO-d_6_) δ 10.71 (s, 1H, NH(1″)), 10.41 (s, 1H, NH(3)), 7.53 (d, *J* = 2.0 Hz, 1H, Ar), 7.48 (d, *J* = 8.4 Hz, 2H, Ar), 7.42 (dd, *J* = 8.3, 2.0 Hz, 1H, Ar), 7.27 (d, *J* = 8.4 Hz, 2H, Ar), 6.74 (d, *J* = 8.3 Hz, 1H, Ar), 4.27–4.18 (m, 3H, -NCH_2_- + pyrrolidine), 3.85 (t, *J* = 9.4 Hz, 1H, pyrrolidine), 3.48–3.41 (m, 1H, pyrrolidine), 2.98 (t, *J* = 2.4 Hz, 1H, -C≡CH), 2.11 (s, 3H, NCH_3_). **^13^C NMR** (101 MHz, DMSO) δ 179.5, 174.1, 171.5, 141.8, 133.9, 132.7, 131.7 (2C), 131.2 (2C), 129.8, 126.1, 120.9, 113.7, 111.8, 77.1, 76.9, 76.1, 73.5, 55.9, 50.1, 34.7, 29.8. **HRMS** (ESI-TOF) *m*/*z*: [M+H]^+^ calculated for [C_23_H_18_Br_2_N_4_O_2_S + Na]^+^ 594.9409; found 594.9416.

**General procedure for the synthesis of conjugates 10a**–**c**. To a solution of the terminal alkyne **9a**–**c** (1 eq.) and TBTA ligand (0.3 eq.) in CH_2_Cl_2_ (5 mL/0.1 mmol of alkyne) under argon, a solution of CuSO_4_*5H_2_O (0.3 eq.) in 200 μL of deionized water was added, followed by a solution of sodium ascorbate (0.6 eq.) in 200 μL of deionized water. The reaction mixture was stirred for 30 min, and then a solution of the N_3_-glutarimide **8** (1 eq.) in CH_2_Cl_2_ (2.5 mL/0.1 mmol of azide) was added dropwise to the resulting dark red solution. The reaction mixture was stirred overnight at room temperature under argon. Then the solvent was evaporated under reduced pressure, and the residue was purified by column chromatography (eluent—CH_2_Cl_2_/CH_3_OH 99:1 → 30:1) to obtain a white solid as a mixture of two diastereomers.

(2′R*,4S*,4′S*)-5″-bromo-4′-(4-chlorophenyl)-1-((1-((2,6-dioxopiperidin-3-yl)methyl)-1H-1,2,3-triazol-4-yl)methyl)-1′-methyl-2-thioxodispiro[imidazolidine-4,3′-pyrrolidine-2′,3″-indoline]-2″,5-dione (10a). From 53 mg of **9a** (0.1 mmol, 1 eq.), 16 mg of TBTA (0.03 mmol, 0.3 eq.), 7.5 mg of CuSO_4_*5H_2_O (0.03 mmol, 0.3 eq.) in 200 μL of deionized water, 12 mg of NaAsc (0.06 mmol, 0.6 eq.) in 200 μL of deionized water, and 17 mg of **8** (0.1 mmol, 1 eq.) 50 mg of compound was obtained **10a** as white solid (71% yield). Compound **10b** was obtained as a mixture of two diastereomers (A:B = 1:1). **^1^H NMR** (400 MHz, DMSO-d6) δ 10.86 (s, 1H^A^ + 1H^B^), 10.70/10.68 (s, 1H^A^ + 1H^B^), 10.26/10.30 (s, 1H^A^ + 1H^B^), 7.53–7.40 (m, 2H^A^ + 2H^B^), 7.39–7.25 (m, 5H^A^ + 5H^B^), 6.76 (dd, *J* = 8.2, 2.7 Hz, 1H^A^ + 1H^B^), 4.78–4.62 (m, 3H^A^ + 3H^B^), 4.56–4.45 (m, 1H^A^ + 1H^B^), 4.23 (t, *J* = 9.7 Hz, 1H^A^ + 1H^B^), 3.89–3.79 (m, 1H^A^ + 1H^B^), 3.45 (t, *J* = 8.8 Hz, 1H^A^ + 1H^B^), 3.15–3.03 (m, 1H^A^ + 1H^B^), 2.54–2.43 (m, 2H^A^ + 2H^B^), 2.10 (s, 3H^A^ + 3H^B^), 1.67–1.49 (m, 2H^A^ + 2H^B^). **^13^C NMR** (101 MHz, DMSO-d_6_) δ 180.3, 174.3 (2C), 172.0, 171.8, 141.9, 141.5, 136.2, 133.5, 132.3, 131.4, 128.8 (2C), 127.8 (2C), 126.3, 124.2, 113.6, 111.8, 77.2, 76.1, 56.1, 50.2, 49.0, 41.3, 35.8, 34.7, 30.9, 21.2. **IR** (cm^−1^): 3400–2800 br., 1697 (C=O). **HRMS** (ESI-TOF) *m*/*z*: [M+H]^+^ calculated for [C_29_H_26_BrClN_8_O_4_S + Na]^+^ 719.0562; found 719.0573.

(2′R*,4S*,4′S*)-4′-(4-bromophenyl)-1-((1-((2,6-dioxopiperidin-3-yl)methyl)-1H-1,2,3-triazol-4-yl)methyl)-1′-methyl-2-thioxodispiro[imidazolidine-4,3′-pyrrolidine-2′,3″-indoline]-2″,5-dione (10b). From 99 mg of **9b** (0.2 mmol, 1 eq.), 32 mg of TBTA (0.06 mmol, 0.3 eq.), 15 mg of CuSO_4_*5H_2_O (0.06 mmol, 0.3 eq.) in 200 μL of deionized water, 24 mg of NaAsc (0.12 mmol, 0.6 eq.) in 200 μL of deionized water, and 34 mg of **8** (0.2 mmol, 1 eq.) 110 mg of compound **10b** was obtained as a white solid (83% yield). Compound **10c** was obtained as a mixture of two diastereomers (A:B = 3:5). **^1^H NMR** (400 MHz, DMSO-d6) δ 10.87 (s, 1H^A^ + 1H^B^), 10.57/10.56 (s, 1H^A^ + 1H^B^), 10.07/10.04 (s, 1H^A^ + 1H^B^), 7.48–7.40 (m, 2H^A^ + 2H^B^), 7.37–7.18 (m, 5H^A^ + 5H^B^), 6.95–6.83 (m, 1H^A^ + 1H^B^), 6.77 (dd, *J* = 7.8, 4.8 Hz, 1H^A^ + 1H^B^), 4.75–4.65 (m, 3H^A^ + 3H^B^), 4.53–4.42 (m, 1H^A^ + 1H^B^), 4.20 (t, *J* = 9.5 Hz, 1H^A^ + 1H^B^), 3.93–3.83 (m, 1H^A^ + 1H^B^), 3.41 (t, *J* = 8.7 Hz, 1H), 3.14–3.02 (m, 1H^A^ + 1H^B^), 2.59–2.38 (m, 2H^A^ + 2H^B^), 2.07 (s, 3H^A^ + 3H^B^), 1.65–1.47 (m, 2H^A^ + 2H^B^). **^13^C NMR** (101 MHz, DMSO-d_6_) δ 180.4, 174.9, 173.1 (2C), 172.5, 142.8, 141.6, 134.3, 131.8, 131.2 (2C), 128.8 (2C), 127.8, 127.1, 123.3, 121.6, 120.8, 109.8, 77.0, 75.9, 56.3, 50.1, 48.9, 41.3, 35.8, 34.6, 30.7, 21.2. **IR** (cm^−1^): 3400–2800 br., 1694 (C=O). **HRMS** (ESI-TOF) *m*/*z*: [M+H]^+^ calculated for [C_29_H_27_BrN_8_O_4_S + Na]^+^ 685.0952; found 6885.0957.

(2′R*,4S*,4′S*)-5″-bromo-4′-(4-bromophenyl)-1-((1-((2,6-dioxopiperidin-3-yl)methyl)-1H-1,2,3-triazol-4-yl)methyl)-1′-methyl-2-thioxodispiro[imidazolidine-4,3′-pyrrolidine-2′,3″-indoline]-2″,5-dione (10c). From 57 mg of **9c** (0.1 mmol, 1 eq.), 16 mg of TBTA (0.03 mmol, 0.3 eq.), 7.5 mg of CuSO_4_*5H_2_O (0.03 mmol, 0.3 eq.) in 200 μL of deionized water, 12 mg of NaAsc (0.06 mmol, 0.6 eq.) in 200 μL of deionized water, and 17 mg of **8** (0.1 mmol, 1 eq.) 51 mg of compound **10c was obtained** as a white solid (69% yield). Compound **10d** was obtained as a mixture of two diastereomers (A:B = 3:4). **^1^H NMR** (400 MHz, DMSO-d6) δ 10.86 (s, 1H^A^ + 1H^B^), 10.70/10.68 (s, 1H^A^ + 1H^B^), 10.31/10.27 (s, 1H^A^ + 1H^B^), 7.52–7.40 (m, 3H^A^ + 3H^B^), 7.39–7.30 (m, 2H^A^ + 2H^B^), 7.30–7.23 (m, 3H^A^ + 3H^B^), 6.76 (dd, *J* = 8.3, 2.8 Hz, 1H^A^ + 1H^B^), 4.78–4.63 (m, 3H^A^ + 3H^B^), 4.56–4.45 (m, 1H^A^ + 1H^B^), 4.21 (t, *J* = 9.2 Hz, 1H^A^ + 1H^B^), 3.89–3.77 (m, 1H^A^ + 1H^B^), 3.45 (t, *J* = 8.9 Hz, 1H^A^ + 1H^B^), 3.15–3.00 (m, 1H^A^ + 1H^B^), 2.60–2.39 (m, 2H^A^ + 2H^B^), 2.10 (s, 3H^A^ + 3H^B^), 1.67–1.48 (m, 2H^A^ + 2H^B^). **^13^C NMR** (101 MHz, DMSO-d_6_) δ 180.3, 174.3, 173.1 (2C), 172.0, 141.9, 141.5, 136.2, 134.0, 132.6, 131.8, 129.8, 128.8 (2C), 127.8 (2C), 126.3, 113.6, 111.8, 77.2, 76.0, 56.0, 50.2, 49.0, 41.3, 35.8, 34.7, 30.9, 21.2. **IR** (cm^−1^): 3400–2800 br., 1697 (C=O). **HRMS** (ESI-TOF) *m*/*z*: [M+H]^+^ calculated for [C_29_H_26_Br_2_N_8_O_4_S + Na]^+^ 763.0057; found 763.0068.

### 3.3. Cell Culture and Treatment

The human HCT116 colon carcinoma cell line (American Type Culture Collection; Manassas, VA), its isogenic HCT116p53^−/−^ subline with deletion of both alleles of the *p53* gene (generated in the Vogelstein laboratory at Johns Hopkins University, Baltimore, MD; provided by B.P.Kopnin), as well as the A549 lung carcinoma cell line and its isogenic A549p53^−/−^ subline with the *TP53* gene deleted using CRISPR/Cas9 technology (gift of A. Bruter, Institute of Gene Biology, Russian Academy of Sciences, Moscow) were cultured in Dulbecco modified Eagle’s medium supplemented with 10% fetal bovine serum (HyClone; Logan, UT, USA), 100 μg/mL penicillin and 100 U/mL streptomycin at 37 °C, 5% CO_2_ in a humidified atmosphere. Newly synthesized compounds were dissolved in DMSO as 10 mM stock solutions, followed by serial dilution in the culture medium immediately before the experiments. Cell viability was determined using MTT tests [52]. Experiments were performed three times, with each concentration tested in duplicate.

### 3.4. Statistics

One-way or two-way analyses of variance (ANOVA), followed by Sidak’s post hoc test for multiple comparisons, were used (GraphPad Prism 9; GraphPad Software, San Diego, CA, USA). A *p* value of <0.05 was taken as evidence of statistical significance.

## 4. Conclusions

Targeting MDM2, we developed a convenient method for the preparation of potential hetero-PROTAC conjugates containing dispiroindolinone-pyrrolidine-thioimidazolone and glutarimide moieties connected via a triazole-containing linker, starting from available precursors: aryl isothiocyanates, glycine, substituted benzaldehydes, chloroacetamide, and ethyl acrylate. The proposed strategy represents a convergent scheme of no more than four sequential steps for each starting compound. Two newly synthesized compounds, 5″-bromo-4′-(4-bromophenyl)-2-(((1-((2,6-dioxopiperidin-3-yl)methyl)-1H-1,2,3-triazol-4-yl)methyl)thio)-1′-methyl-1-phenyldispiro[imidazole-4,3′-pyrrolidine-2′,3″-indoline]-2″,5(1H)-dione 6b and 5″-bromo-4′-(4-bromophenyl)-1′-methyl-2-thioxodispiro[imidazolidine-4,3′-pyrrolidine-2′,3″-indoline]-2″,5-dione 7b showed preferential cytotoxicity against HCT116 cells (wild-type p53) compared to the isogenic p53-null subline, suggesting their potential for stabilization of the pro-apoptotic p53, at least for individual cell types.

## Figures and Tables

**Table 1:** Cytotoxicity of compounds **6 and 7** in the HCT116 cell line (wild-type p53).

Compound	Mean IC_50_, μM
**6a**	11.2 ± 2.3
**6b**	12.5 ± 3.4
**6c**	22.5 ± 3.3
**10b**	38.1 + 3.2
**10c**	38.0 + 2.6
**11a**	44.3 ± 3.2
**11b**	13.0 ± 2.3

## Data Availability

The raw data supporting the conclusions of this article will be made available by the authors on request.

## Supplementary Materials

The following supporting information can be downloaded at: https://www.mdpi.com/article/10.3390/molecules31101602/s1 (^1^H and _13_C NMR spectra of compounds **1**–**10**, IR spectra of compounds **6** and **10**, HPLC of compounds **6a** and **6b** and cells survival curves), for compounds **6a**, **6b**, **6c** and **7b**.

## References

[1] Levine A.J. (2022). Targeting the P53 Protein for Cancer Therapies: The Translational Impact of P53 Research. Cancer Res..

[2] Aubrey B.J., Kelly G.L., Janic A., Herold M.J., Strasser A. (2018). How does p53 induce apoptosis and how does this relate to p53-mediated tumour suppression?. Cell Death Differ..

[3] Wu D., Prives C. (2018). Relevance of the p53–MDM2 axis to aging. Cell Death Differ..

[4] Zhu H., Gao H., Ji Y., Zhou Q., Du Z., Tian L., Jiang Y., Yao K., Zhou Z. (2022). Targeting p53–MDM2 interaction by small-molecule inhibitors: Learning from MDM2 inhibitors in clinical trials. J. Hematol. Oncol..

[5] Beloglazkina A., Zyk N., Majouga A., Beloglazkina E. (2020). Recent small-molecule inhibitors of the p53–mdm2 protein–protein interaction. Molecules.

[6] Synoradzki K.J., Bartnik E., Czarnecka A.M., Fiedorowicz M., Firlej W., Brodziak A., Stasinska A., Rutkowski P., Grieb P. (2021). TP53 in Biology and Treatment of Osteosarcoma. Cancers.

[7] Konopleva M., Martinelli G., Daver N., Papayannidis C., Wei A., Higgins B., Ott M., Mascarenhas J., Andreeff M. (2020). MDM2 inhibition: An important step forward in cancer therapy. Leukemia.

[8] Patel K.R., Patel H.D. (2020). p53: An Attractive Therapeutic Target for Cancer. Curr. Med. Chem..

[9] Ivanenkov Y.A., Vasilevski S.V., Beloglazkina E.K., Kukushkin M.E., Machulkin A.E., Veselov M.S., Chufarova N.V., Chernyagina E.S., Vanzcool A.S., Zyk N.V. (2015). Design, synthesis and biological evaluation of novel potent MDM2/p53 small-molecule inhibitors. Bioorg Med. Chem. Lett..

[10] Bora D., Kaushal A., Shankaraiah N. (2021). Anticancer potential of spirocompounds in medicinal chemistry: A pentennial expedition. Eur. J. Med. Chem..

[11] Arnhold V., Schmelz K., Proba J., Winkler A., Wünschel J., Toedling J., Deubzer H.E., Künkele A., Eggert A., Schulte J.H. (2017). Reactivating TP53 signaling by the novel MDM2 inhibitor DS-3032b as a therapeutic option for high-risk neuroblastoma. Oncotarget.

[12] Gomez-Monterrey I., Bertamino A., Porta A., Carotenuto A., Musella S., Aquino C., Granata I., Sala M., Brancaccio D., Picone D. (2010). Identification of the Spiro(oxindole-3,3′-thiazolidine)-Based Derivatives as Potential p53 Activity Modulators. J. Med. Chem..

[13] Wang C., Zhang Y., Chen W., Wu Y., Xing D. (2024). New-generation advanced PROTACs as potential therapeutic agents in cancer therapy. Mol. Cancer.

[14] Yang G., Zhong H., Xia X., Qb Z., Wang C., Li S. (2022). Potential application of proteolysis targeting chimera (PROTAC) modification technology in natural products for their targeted protein degradation. Food Sci. Human. Wellness.

[15] He S., Ma J., Fang Y., Liu Y., Wu S., Dong G., Wang W., Sheng C. (2021). Homo-PROTAC mediated suicide of MDM2 to treat non-small cell lung cancer. Acta Pharma Sin. B.

[16] Powell C.E., Du G., Bushman J.W., He Z., Zhang T., Fischer E.S., Gray N.S. (2021). Selective degradation-inducing probes for studying cereblon (CRBN) biology. RSC Med. Chem..

[17] Vetma V., O’Connor S., Ciulli A. (2025). Development of PROTAC Degrader Drugs for Cancer. Annu. Rev. Cancer Biol..

[18] Rathkopf D.E., Patel M.R., Choudhury A.D., Rasco D., Lakhani N., Hawley J., Srinivas E.S., Aparicio A., Narayan V., Runcie K.D. (2025). Safety and clinical activity of BMS-986365 (CC-94676), a dual androgen receptor ligand-directed degrader and antagonist, in heavily pretreated patients with metastatic castration-resistant prostate cancer. Ann. Oncol..

[19] Anifowose A., Agbowuro A.A., Yang X., Wang B. (2020). Anticancer strategies by upregulating p53 through inhibition of its ubiquitination by MDM2. Med. Chem. Res..

[20] Ha S., Luo G., Xiang H.A. (2022). Comprehensive Overview of Small-Molecule Androgen Receptor Degraders: Recent Progress and Future Perspectives. J. Med. Chem..

[21] Wang B., Liu J., Tandon I., Wu S., Teng P., Liao J., Tang W. (2021). Development of MDM2 degraders based on ligands derived from Ugi reactions: Lessons and discoveries. Eur. J. Med. Chem..

[22] Ito T., Ando H., Suzuki T., Ogura T., Hotta K., Imamura Y., Yamaguchi Y., Handa H. (2010). Identification of a Primary Target of Thalidomide Teratogenicity. Science.

[23] Angers S., Li T., Yi X., MacCoss M.J., Moon R.T., Zheng N. (2006). Molecular architecture and assembly of the DDB1–CUL4A ubiquitin ligase machinery. Nature.

[24] Lue J.K., Stevens D.A., Williams M.E., Westin J., Ewesuedo R., McDonald A., Agarwal S., Henrick P., Perea R., Gollob J. (2022). Phase 1 Study of KT-413, a Targeted Protein Degrader of IRAK4 and IMiD Substrates, in Adult Patients with Relapsed or Refractory B-Cell Non-Hodgkin Lymphoma. Blood.

[25] Petzold G., Fischer E.S., Thomä N.H. (2016). Structural basis of lenalidomide-induced CK1α degradation by the CRL4CRBN ubiquitin ligase. Nature.

[26] Matyskiela M.E., Lu G., Ito T., Pagarigan B., Lu C.-C., Miller K., Fang W., Wang N.-Y., Nguyen D., Houston J. (2016). A novel cereblon modulator recruits GSPT1 to the CRL4CRBN ubiquitin ligase. Nature.

[27] Winter G.E., Buckley D.L., Paulk J., Roberts J.M., Souza A., Dhe-Paganon S., Bradner J.E. (2015). Phthalimide conjugation as a strategy for in vivo target protein degradation. Science.

[28] Lu J., Qian Y., Altieri M., Dong H., Wang J., Raina K., Hines J., Winkler J.D., Crew A.P., Coleman K. (2015). Hijacking the E3 Ubiquitin Ligase Cereblon to Efficiently Target BRD4. Chem. Biol..

[29] Khuzhakhmetova L.R., Ananeva A.A., Kantin G.P., Dar’in D.V., Bunev A.S., Ebeling S., Herrmann A., Hartmann M.D., Kalinin S.A., Bakulina O.Y. (2025). Synthesis of α-(azidomethyl)glutarimide and its applicationin construction of potential Cereblon ligands via the CuAAC reaction. Mendeleev Commun..

[30] Kantin G., Golubev P., Sapegin A., Bunev A., Dar’in D. (2023). N-Boc-α-diazo glutarimide as efficient reagent for assembling N-heterocycle-glutarimide diads via Rh(II)-catalyzed N–H insertion reaction. Beilstein J. Org. Chem..

[31] Han X., Sun Y. (2022). Strategies for the discovery of oral PROTAC degraders aimed at cancer therapy. Cell Rep. Phys. Sci..

[32] Gough S.M., Flanagan J.J., The J., Andreoli M., Rousseau E., Pannone M., Bookbinder M., Willard R., Davenport K., Bortolon E. (2024). Oral Estrogen Receptor PROTAC Vepdegestrant (ARV-471) Is Highly Efficacious as Monotherapy and in Combination with CDK4/6 or PI3K/mTOR Pathway Inhibitors in Preclinical ER+ Breast Cancer Models. Clin. Cancer Res..

[33] Bricelj A., Steinebach C., Kuchta R., Gütschow M., Sosič I. (2021). E3 ligase ligands in successful PROTACs: An overview of syntheses and linker attachment points. Front. Chem..

[34] Majouga A.G., Beloglazkina E.K., Beloglazkina A.A., Kukushkin M.E., Ivanenkov Y.A., Veselov M.S. (2015). New Dispiro-Indolinones, Inhibitors of mdm2/p53 Interaction, Method of Production and Use.

[35] Ivanenkov Y.A., Kukushkin M.E., Beloglazkina A.A., Shafikov R.R., Barashkin A.A., Ayginin A.A., Serebryakova M.V., Majouga A.G., Skvortsov D.A., Tafeenko V.A. (2023). Synthesis and biological evaluation of novel dispiro-indolinones with anticancer activity. Molecules.

[36] Beloglazkina A.A., Karpov N.A., Mefedova S.R., Polyakov V.S., Skvortsov D.A., Kalinina M.A., Tafeenko V.A., Majouga A.G., Zyk N.V., Beloglazkina E.K. (2019). Synthesis of dispirooxindoles containing N-unsubstituted heterocyclic moieties and study of their anticancer activity. Russ. Chem. Bull..

[37] Kukushkin M.E., Skvortsov D.A., Kalinina M.A., Tafeenko V.A., Burmistrov V.V., Butov G.M., Zyk N.V., Majouga A.G., Beloglazkina E.K. (2020). Synthesis and cytotoxicity of oxindoles dispiro derivatives with thiohydantoin and adamantane fragments. Phosphorus Sulfur. Silicon.

[38] Polyakov V.S., Pervakova E.V., Zyk N.V., Beloglazkina E.K. (2025). Synthesis of bis(dispiro[indolinone-pyrrolidine-imidazolones]). Russ. Chem. Bull..

[39] Polyakov V.S., Pervakova E.V., Grishin Y.K., Beloglazkina E.K. (2025). Bis-dispiro-indolinone-pyrrolidine-imidazolones with polymethylene linkers between imidazolone fragments. Tetrahedron Lett..

[40] Kuznetsova O.Y., Antipin R.L., Udina A.V., Krasnovskaya O.O., Beloglazkina E.K., Terenin V.I., Koteliansky V.E., Zyk N.V., Majouga A.G. (2016). An improved protocol for synthesis of 3-substituted 5-arylidene-2-thiohydantoins: Two-step procedure alternative to classical methods. J. Heterocycl. Chem..

[41] Patel G., Shah V.R., Nguyen T.A., Deshmukh K., Patel G., Shah V.R., Nguyen T.A., Deshmukh K. (2024). Spirooxindole: Chemistry, Synthesis, Characterization and Biological Significance.

[42] Izmest’ev A.N., Streltsov A.A., Kravchenko A.N., Gazieva G.A. (2023). 5-Arylmethylidene-2-iminothiazolidin-4-ones in the synthesis of novel dispiro-fused oxindolepyrrolidineiminothiazolidinones. Chem. Heterocycl. Compd..

[43] de Silva N.H., Dahdah A., Blanch E.W., Hügel H.M., Maniam S. (2022). Regioselective pyrrolizidine bis-spirooxindoles as efficient anti-amyloidogenic agents. Eur. J. Med. Chem..

[44] Sharma R., Yadav L., Nasim A.A., Yadav R.K., Chen R.H., Kumari N., Ruiqi F., Sharon A., Sahu N.K., Ippagunta S.K. (2023). Chemo-/Regio-Selective Synthesis of Novel Functionalized Spiro[pyrrolidine-2;3′-oxindoles] under Microwave Irradiation and Their Anticancer Activity. Molecules.

[45] Du Y., Liu Y., Guo H., Liu R., Zhou R. (2023). Chemo- and Diastereoselective Synthesis of Spirooxindole-pyrazolines and Pyrazolones via P(NMe_2_)_3_ -Mediated Substrate-Controlled Annulations of Azoalkenes with α-Dicarbonyl Compounds. Org. Lett..

[46] Dubey S., Pal A., Roy S., Sasmal S., Tamrakar A., Jana R., Das T. (2023). Recent advances in the (3+2) cycloaddition of azomethine ylide. New J. Chem..

[47] Zhang X., Ma X., Qiu W., Awad J., Zhang W. (2022). Double [3+2] cycloadditions for diastereoselective synthesis of spirooxindole pyrrolizidines. Green. Process. Synth..

[48] Alshahrani S., Al-Majid A., Alamary A., Ali M., Altowyan M., Ríos-Gutiérrez M., Yousuf S., Barakat A. (2023). Synthesis and Characterization of New Spirooxindoles Including Triazole and Benzimidazole Pharmacophores via [3+2] Cycloaddition Reaction: An MEDT Study of the Mechanism and Selectivity. Molecules.

[49] Izmest’ev A.N., Kravchenko A.N., Gazieva G.A. (2023). A 1;3-dipolar cycloaddition of azomethine ylides to imidazo[4;5-e]thiazolo[2;3-c][1;2;4]triazine oxindolylidene derivatives in the synthesis of novel spirooxindole derivatives. Chem. Heterocycl. Compd..

[50] Degtiarev A.D., Barashkin A.A., Grishin Y.K., Roznyatovsky V.A., Tafeenko V.A., Tarasenko E.A., Beloglazkina E.K. (2025). Synthesis of Chiral Dispiro-indolinone-pyrrolidine-rhodanines using (R)/(S)-1-(2;4-dimethoxyphenyl)ethylamine. ChemistrySelect.

[51] Majouga A.G., Beloglazkina E.K., Vatsadze S.Z., Frolova N.A., Zyk N.V. (2004). Synthesis of isomeric 3-phenyl-5-(pyridylmethylene)-2-thiohydantoins and their S-methylated derivatives. Molecular and crystal structures of (5Z)-3-phenyl-5-(pyridin-2-ylmethylene)-2-thiohydantoin and (5Z)-2-methylthio-3-phenyl-5-(pyridin-2-ylmethylene)-3,5-dihydro-4H-imidazol-4-one. Russ. Chem. Bull..

[52] Wanner M.J., Koomen G.J. (1988). 2-Substituted Glutarimides via Preformed Wittig Reagents. Synthesis.

[53] Krasavin M., Adamchik M., Bubyrev A., Heim C., Maiwald S., Zhukovsky D., Zhmurov P., Bunev A., Hartmann M.D. (2023). Synthesis of novel glutarimide ligands for the E3 ligase substrate receptor Cereblon (CRBN): Investigation of their binding mode and antiproliferative effects against myeloma cell lines. Eur. J. Med. Chem..

[54] Polyakov V.S., Barashkin A.A., Grishin Y.K., Tarasenko E.A., Beloglazkina E.K. (2025). Synthesis of chiral dispiro-indolinone-pyrrolidine-imidazolones using (r) or(s)-1-(2,4-dimethoxyphenyl)ethyl amine. ChemistrySelect.

[55] Dai H., Liu G., Zhang X., Yan H., Lu C. (2016). Pyrrolylmethyl Functionalized o-Carborane Derivatives. Organometallics.

[56] Post E.A.J., Fletcher S.P. (2019). Controlling the Kinetics of Self-Reproducing Micelles by Catalyst Compartmentalization in a Biphasic System. J. Org. Chem..

[57] Jaiswal S., Siddiqui I.R., Rajput S. (2013). A novel α-haloacid based clay-catalysed expeditious synthesis of pyrazoloimidazole-2-thione-N-nucleosides. Int. J. Sci. Eng. Res..

[58] Ding M.W., Sun Y., Liu Z.J. (2003). An Efficient Synthesis of 2-Alkylthio- 5-phenylmethylidene-4 H -imidazolin-4-ones. Synth. Commun..

[59] Sirivolu V.R.S., Vernekar K.V., Marchand C., Naumova A., Chergui A., Renaud A., Stephen A.G., Chen F., Sham Y.Y., Pommier Y. (2012). 5-Arylidenethioxothiazolidinones as inhibitors of tyrosyl–DNA phosphodiesterase I. J. Med. Chem..

[60] Korohoda M.J. (1980). Introduction of selenium into heterocyclic compounds. i: Synthesis of 3-aryl-2-selenohydantoins with double bond at c-5. Pol. J. Chem..

[61] Guk D.A., Krasnovskaya O.O., Zyk N.V., Beloglazkina E.K. (2020). Convenient synthesis of 2-thioimidazolone/menadione conjugates via a two-step sequence starting with direct amination of menadione. SynOpen.

[62] Tsymbal S.A., Moiseeva A.A., Agadzhanian N.A., Efimova S.S., Markova A.A., Guk D.A., Krasnovskaya O.O., Alpatova V.M., Zaitsev A.V., Shibaeva A.V. (2021). Copper-containing nanoparticles and organic complexes: Metal reduction triggers rapid cell death via oxidative burst. Int. J. Mol. Sci..

