# Supplementary material for: Dispiroindolinone–Glutarimide Conjugates: Synthesis and Evaluation as Potential Hetero-PROTACs for p53 Reactivation
Source: Molecules. 2026 May 10;31(10):1602. doi: 10.3390/molecules31101602 (PMC13209440; doi:10.3390/molecules31101602)
Supplement: Supplementary file 1 [file molecules-31-01602-s001.zip › molecules-4227578-supplementary.pdf]

Supporting Information

Dispiroindolinone–Glutarimide Conjugates: Synthesis and Evaluation as Potential Hetero-PROTACs for p53 Reactivation

Vladislav S. Polyakov [1], Yuri K. Grishin [1], Viktor A. Tafeenko [1], Ekaterina S. Ivanova [2,3], Sofya S. Pogodaeva [4], Daniil V. Moldavskii [4], Alexander A. Shtil [2,3] and Elena K. Beloglazkina [1,*]

[1] Department of Chemistry, M.V. Lomonosov Moscow State University, Leninskie Gory 1-3, 119991 Moscow, Russia
[2] Institute of Experimental Oncology and Carcinogenesis, Blokhin National Research Center of Oncology, 24 Kashirskoye Shosse, 115522 Moscow, Russia
[3] Institute of Cyber Intelligence, National Research Nuclear University MEPhI, 31 Kashirskoye shosse, 115409 Moscow, Russia
[4] Center for Molecular and Biological Technologies, ITMO University, 9 Lomonosov Street, 197101 Saint-Petersburg, Russia

Table of contents

1. NMR Spectra 2
2. IR Spectra 24
3. HPLC 27
4. Biological testing 28

1. NMR Spectra

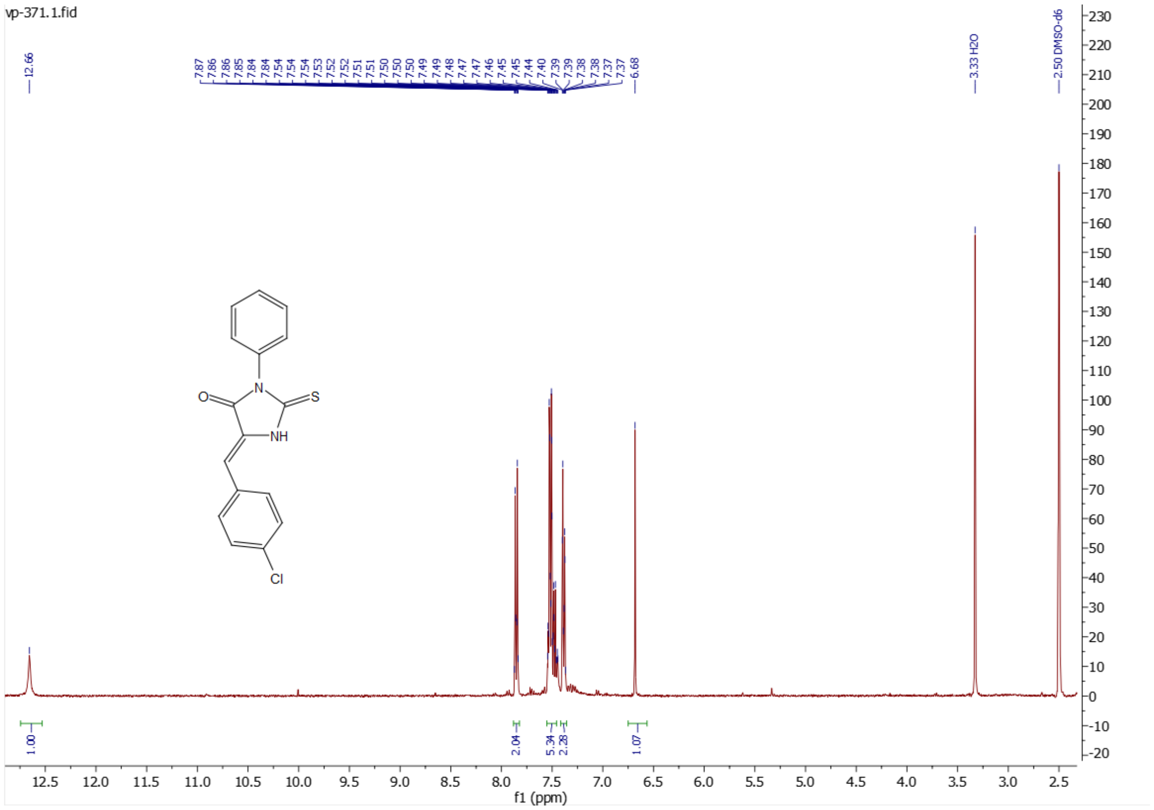

Figure S1. ^{1}H NMR spectrum of compound **1a**

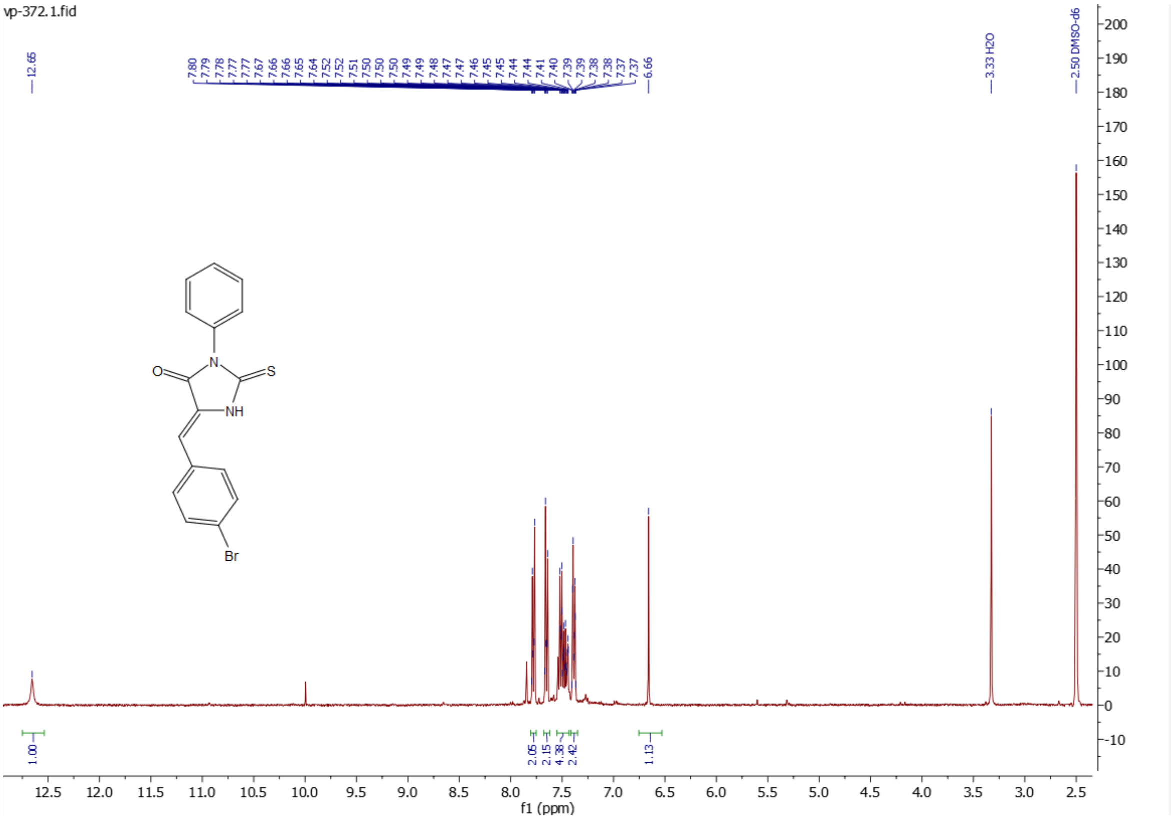

Figure S2. ^{1}H NMR spectrum of compound **1b**

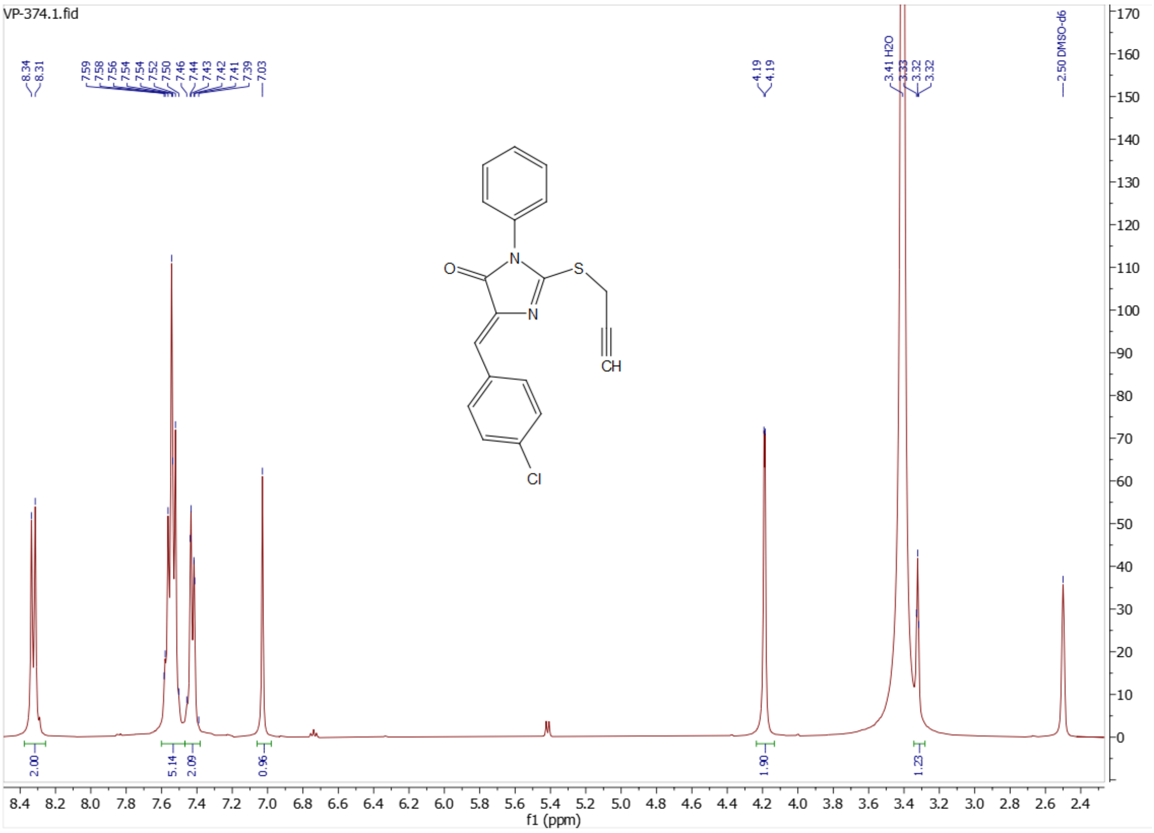

Figure S3. ^{1}H NMR spectrum of compound **2a**

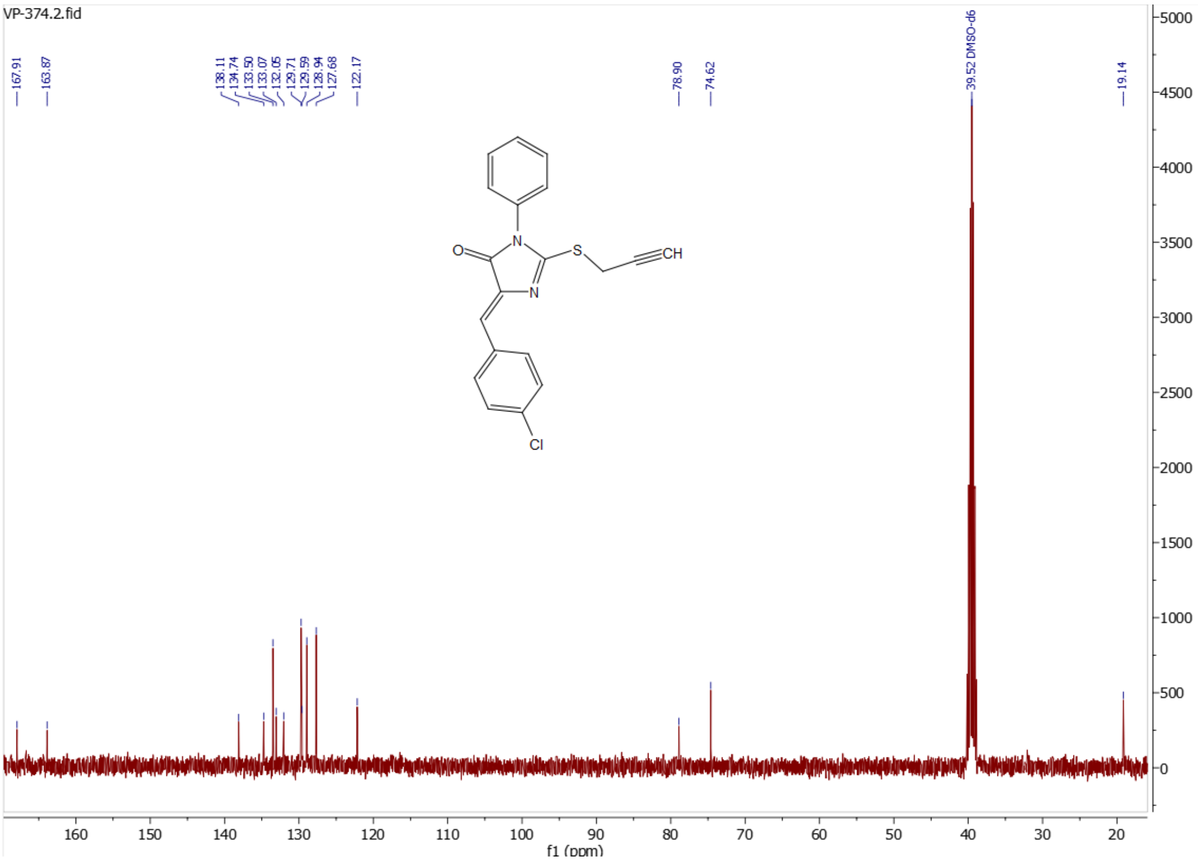

Figure S4. ^{13}C NMR spectrum of compound **2a**

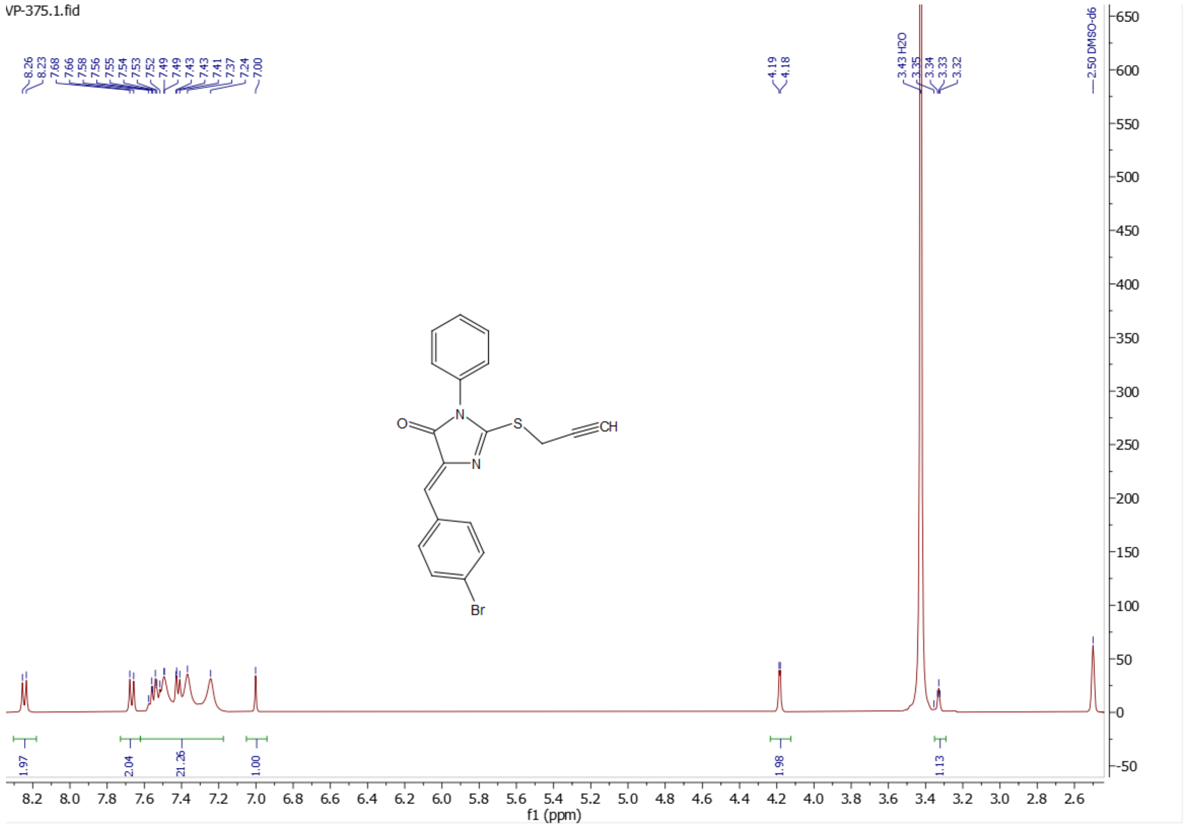

Figure S5. ^{1}H NMR spectrum of compound **2b**

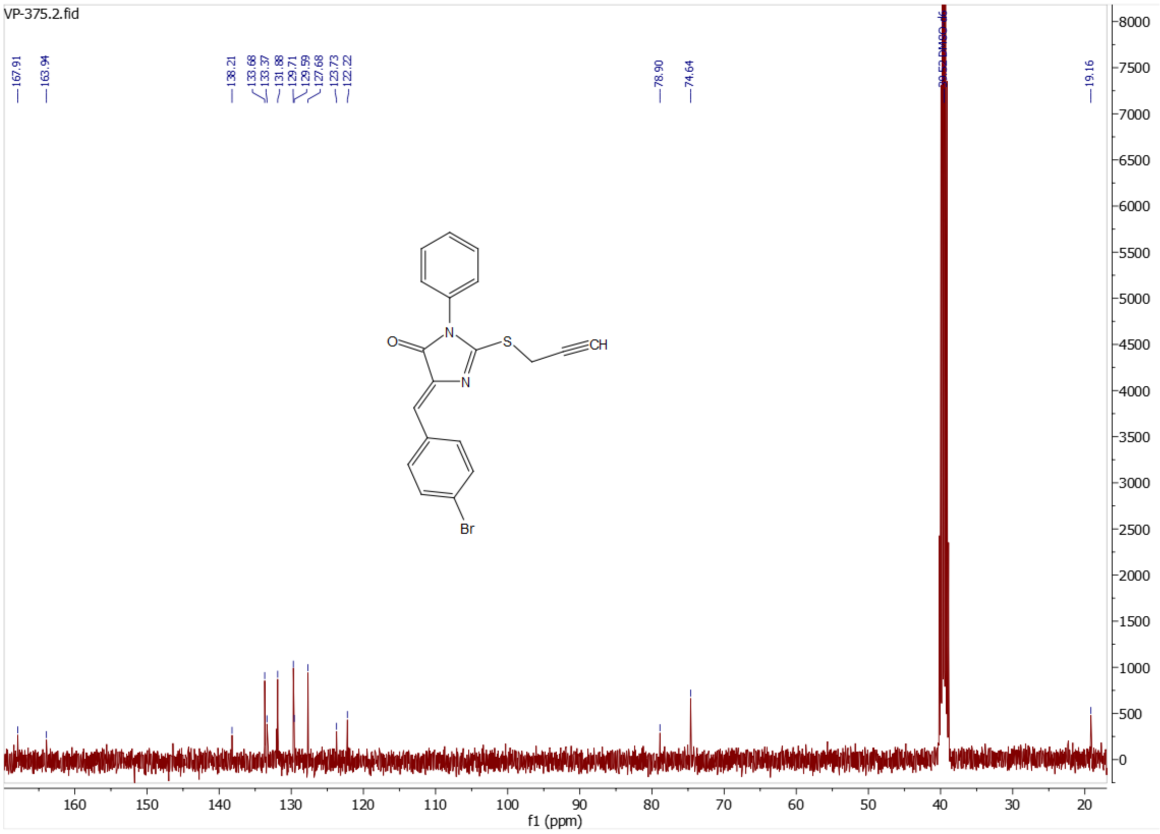

Figure S6. ^{13}C NMR spectrum of compound **2b**

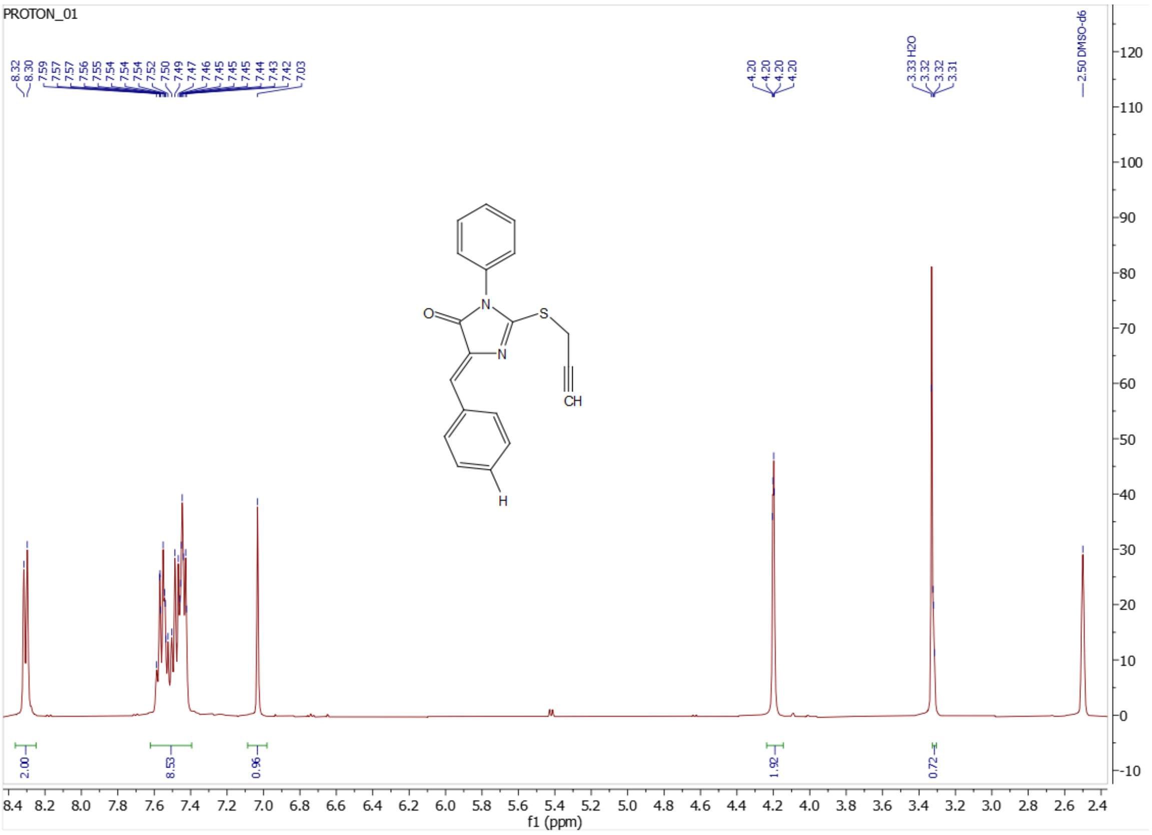

Figure S7. ^{1}H NMR spectrum of compound **2c**

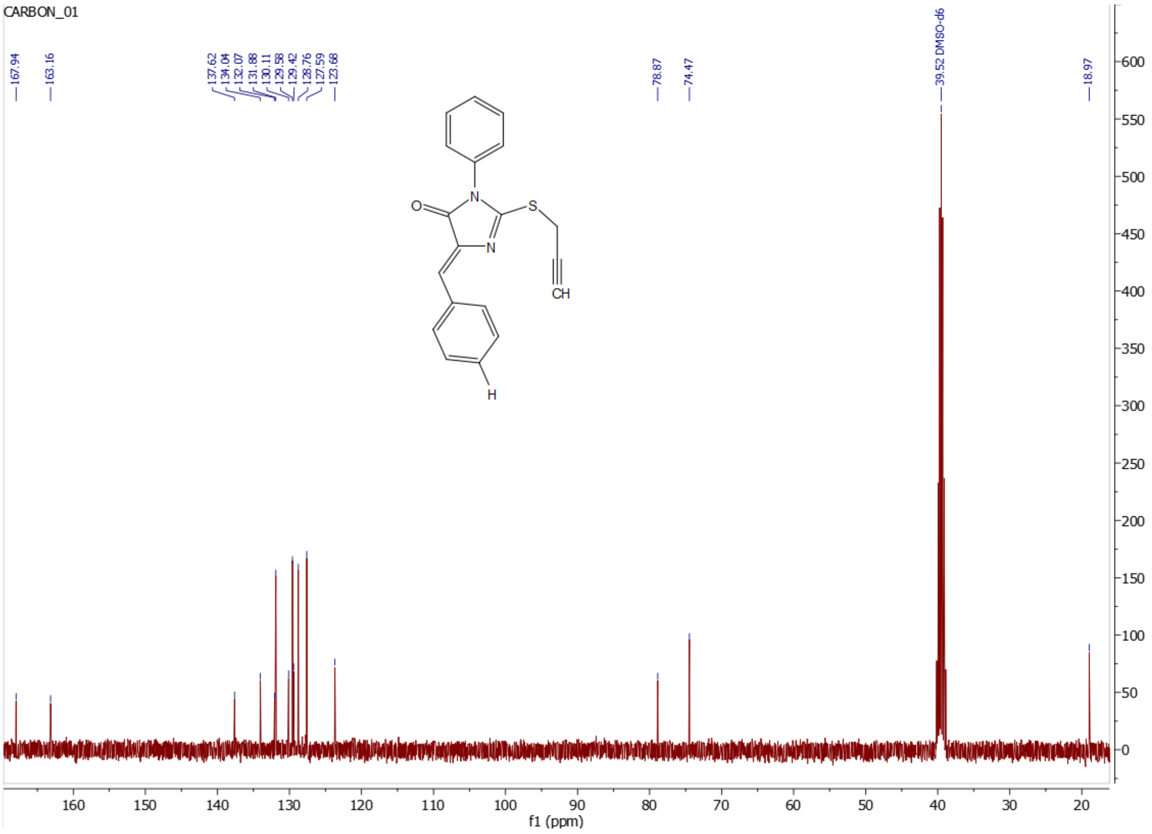

Figure S8. ^{13}C NMR spectrum of compound **2c**

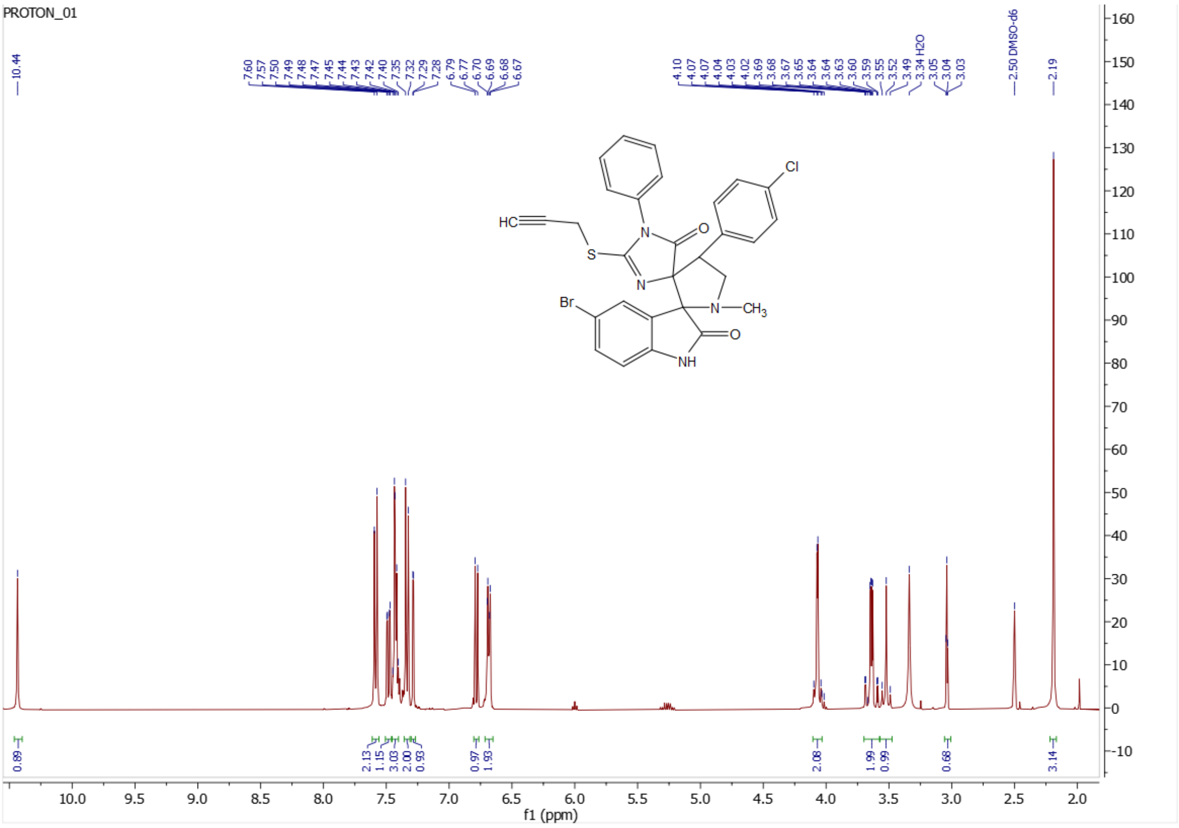

Figure S9. ^{1}H NMR spectrum of compound **3a**

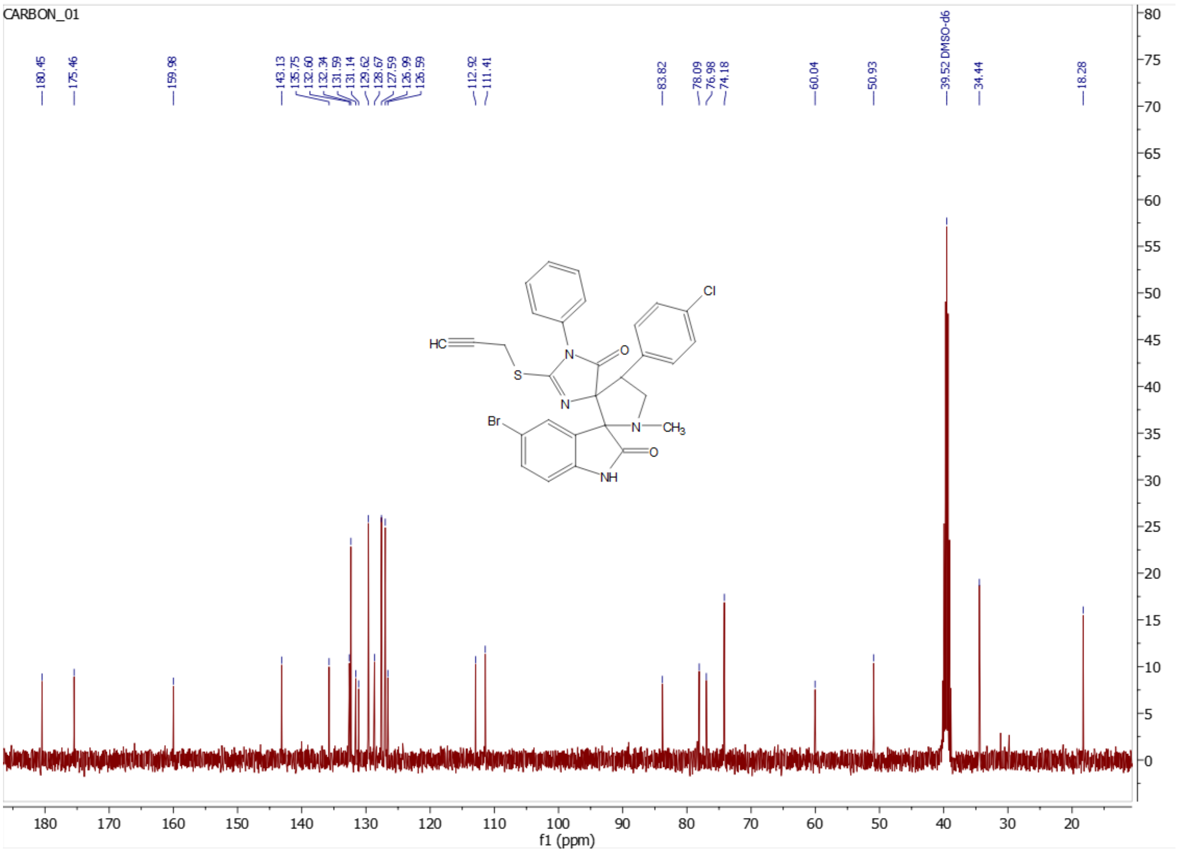

Figure S10. ^{13}C NMR spectrum of compound **3a**

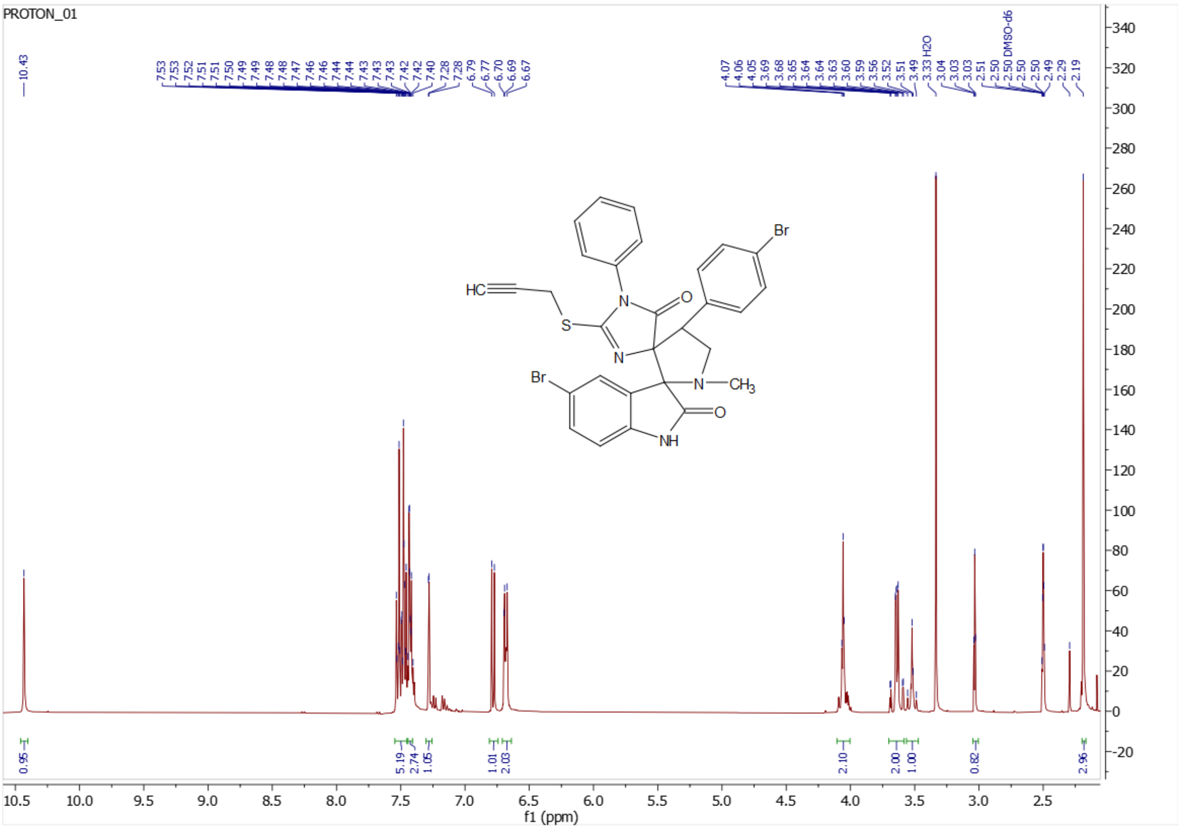

Figure S11. ^{1}H NMR spectrum of compound **3b**

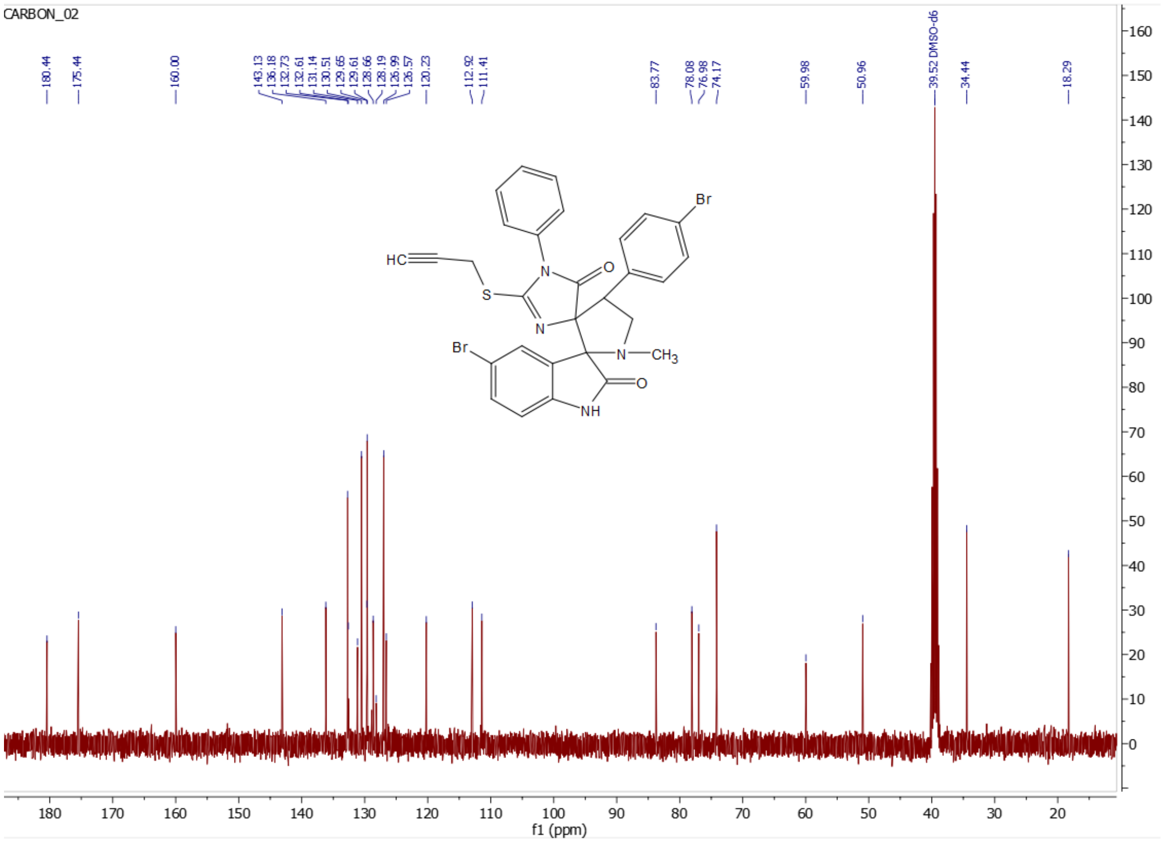

Figure S12. ^{1}H NMR spectrum of compound **3b**

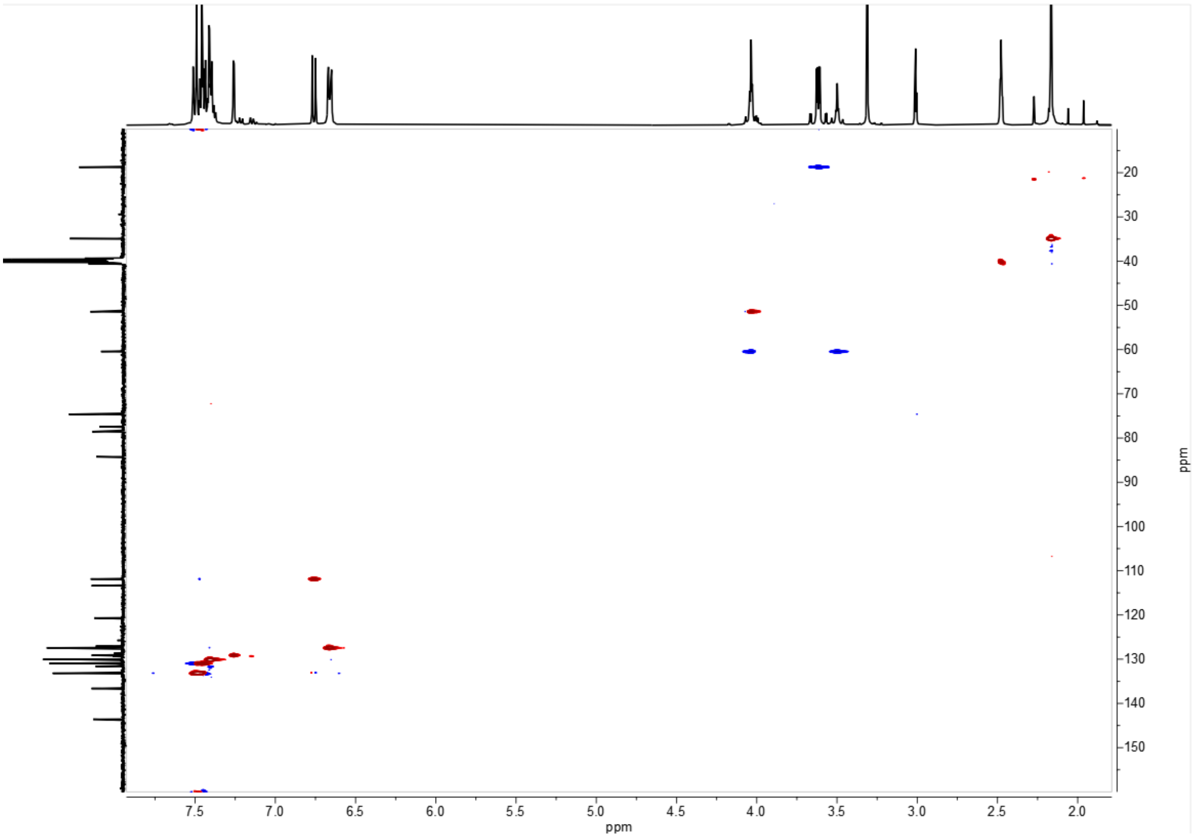

Figure S13. HSQC ^{1}H-^{13}C spectrum of compound **3b**

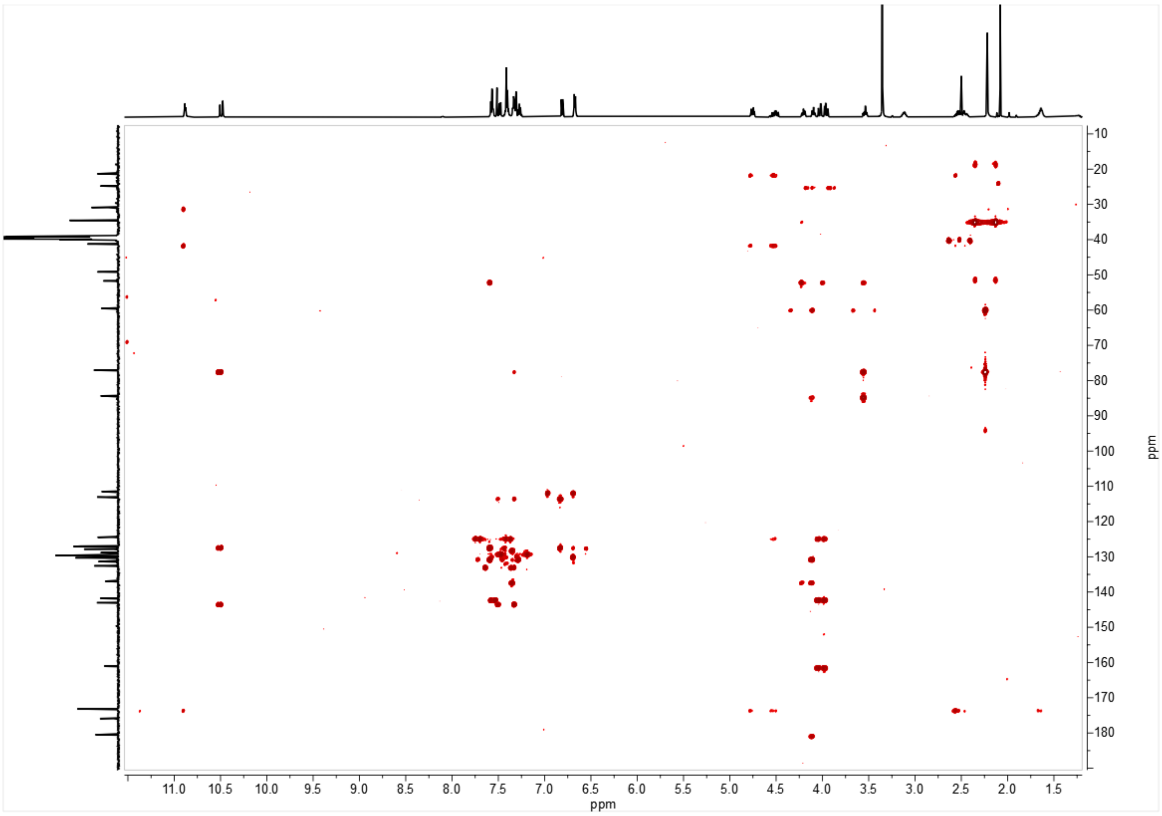

Figure S14. HMBC ^{1}H-^{13}C spectrum of compound **3b**

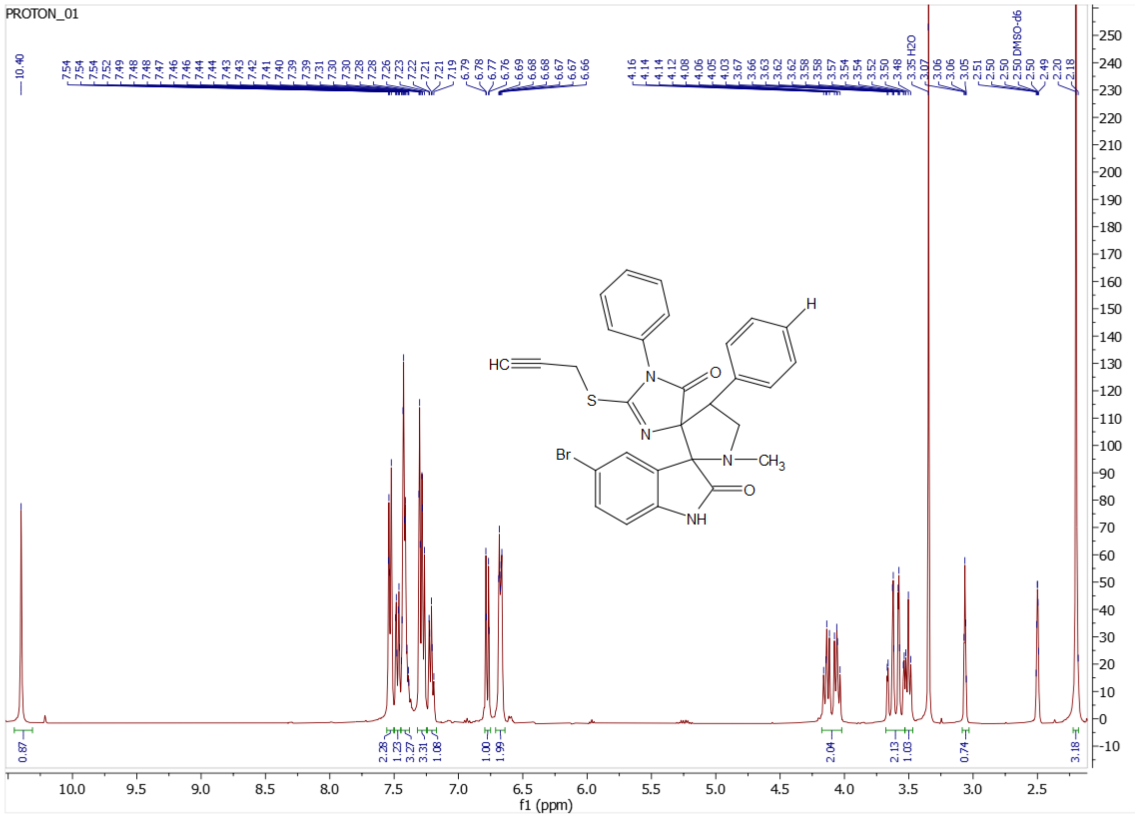

Figure S15. ^{1}H NMR spectrum of the compound **3c**

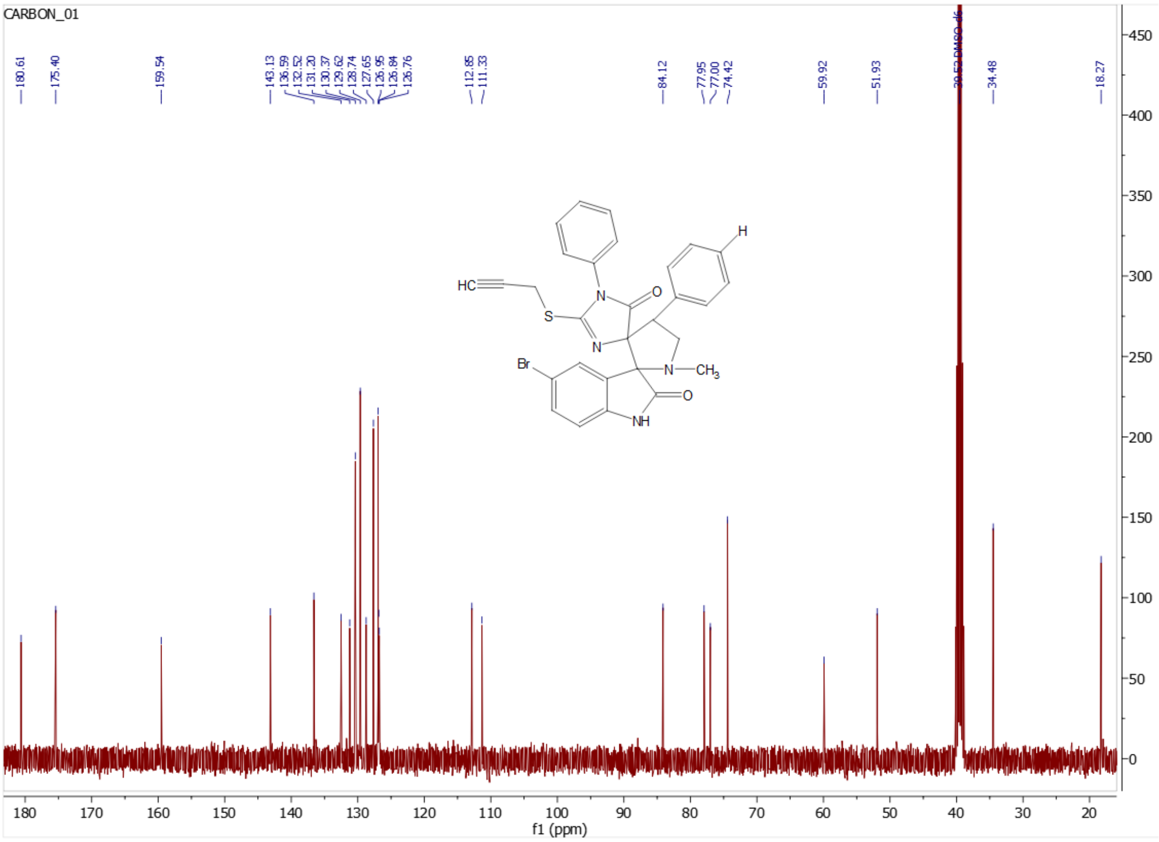

Figure S16. ^{13}C NMR spectrum of compound **3c**

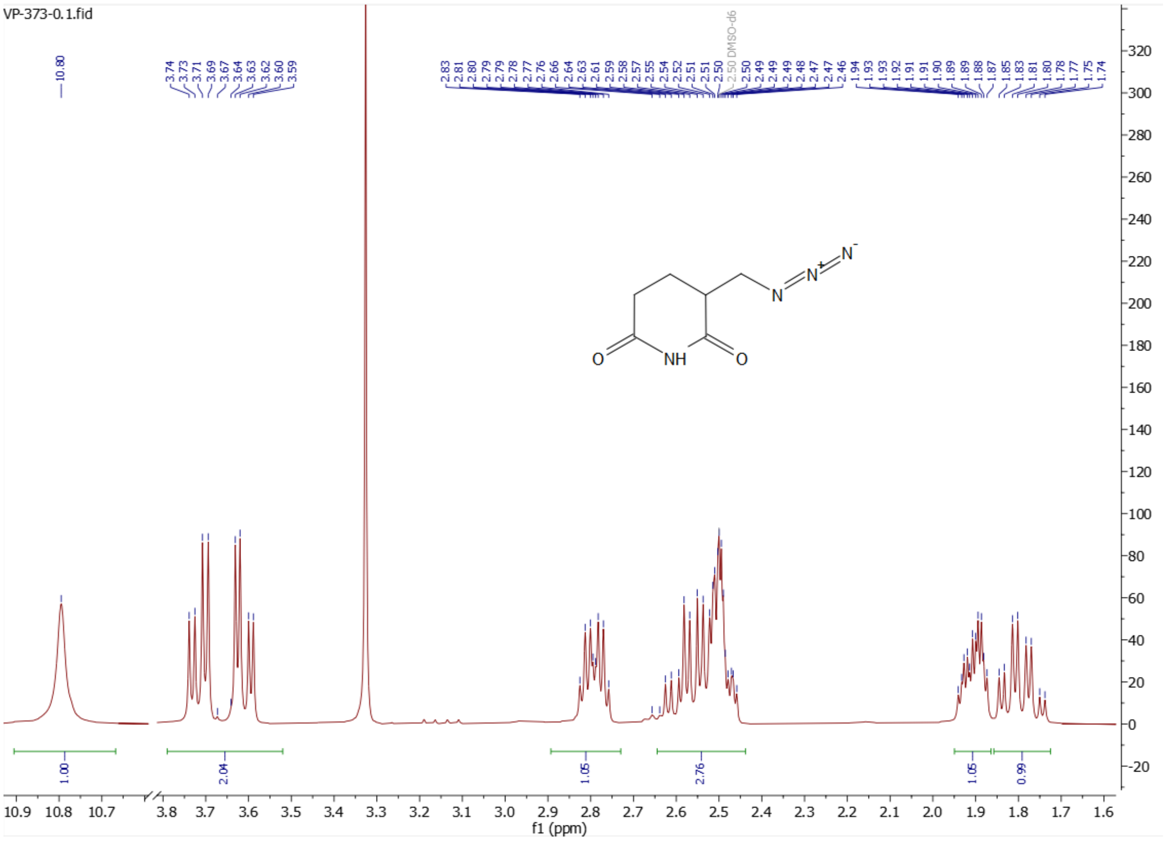

Figure S17. ^{1}H NMR spectrum of the compound **5**

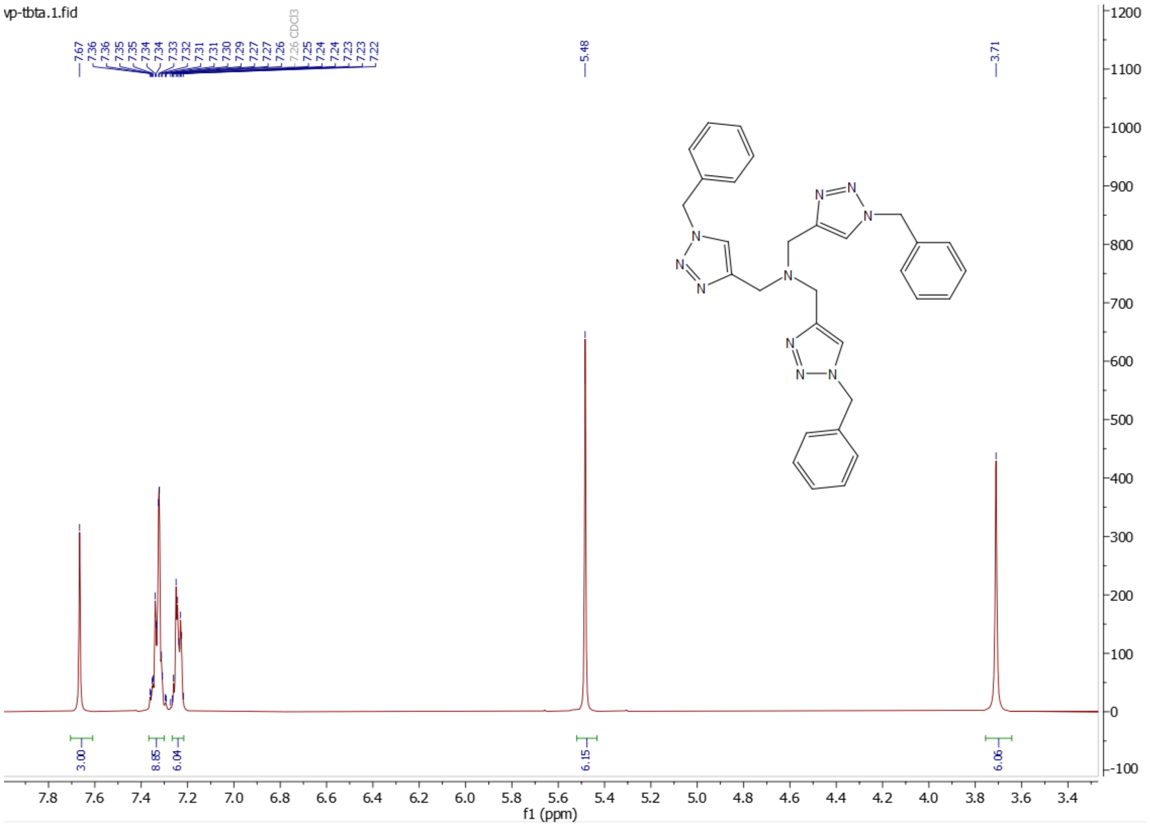

Figure S18. ^{1}H NMR spectrum of **TBTA** catalyst

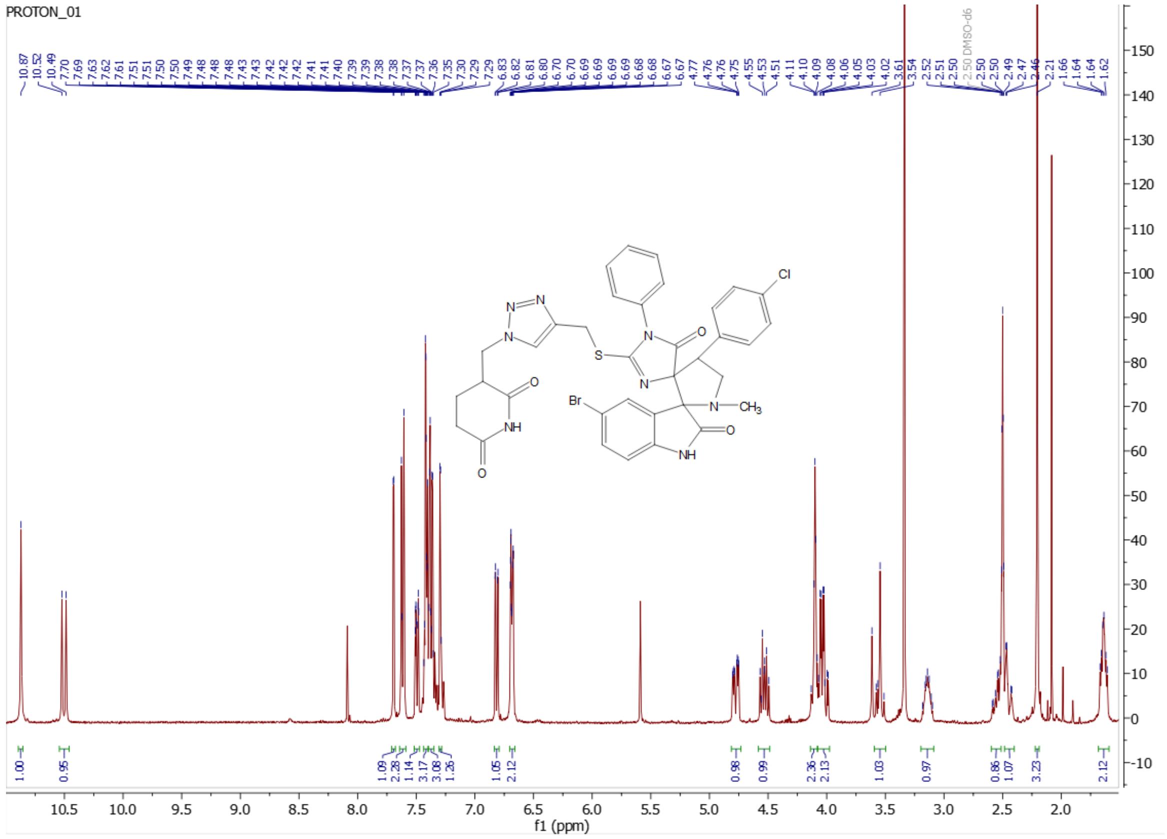

Figure S19. ^{1}H NMR spectrum of compound **6a**

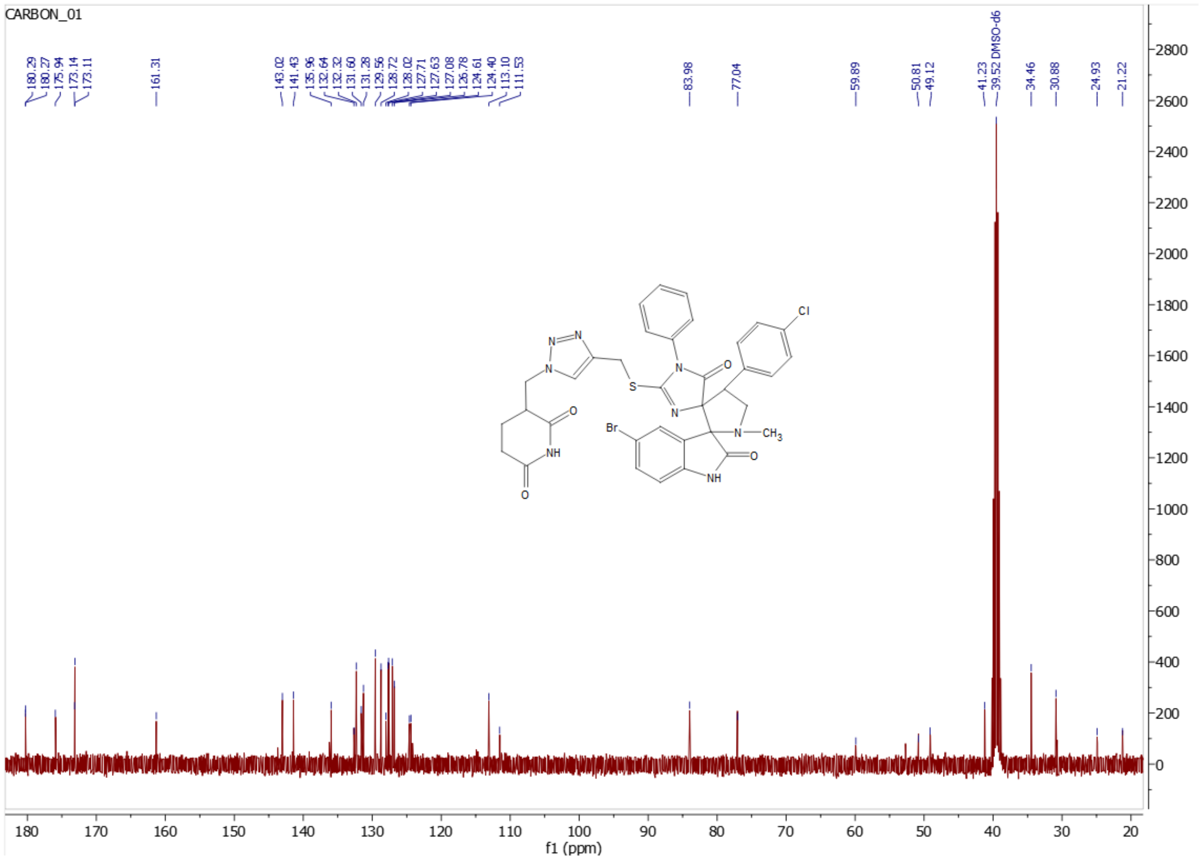

Figure S20. ^{13}C NMR of compound **6a**

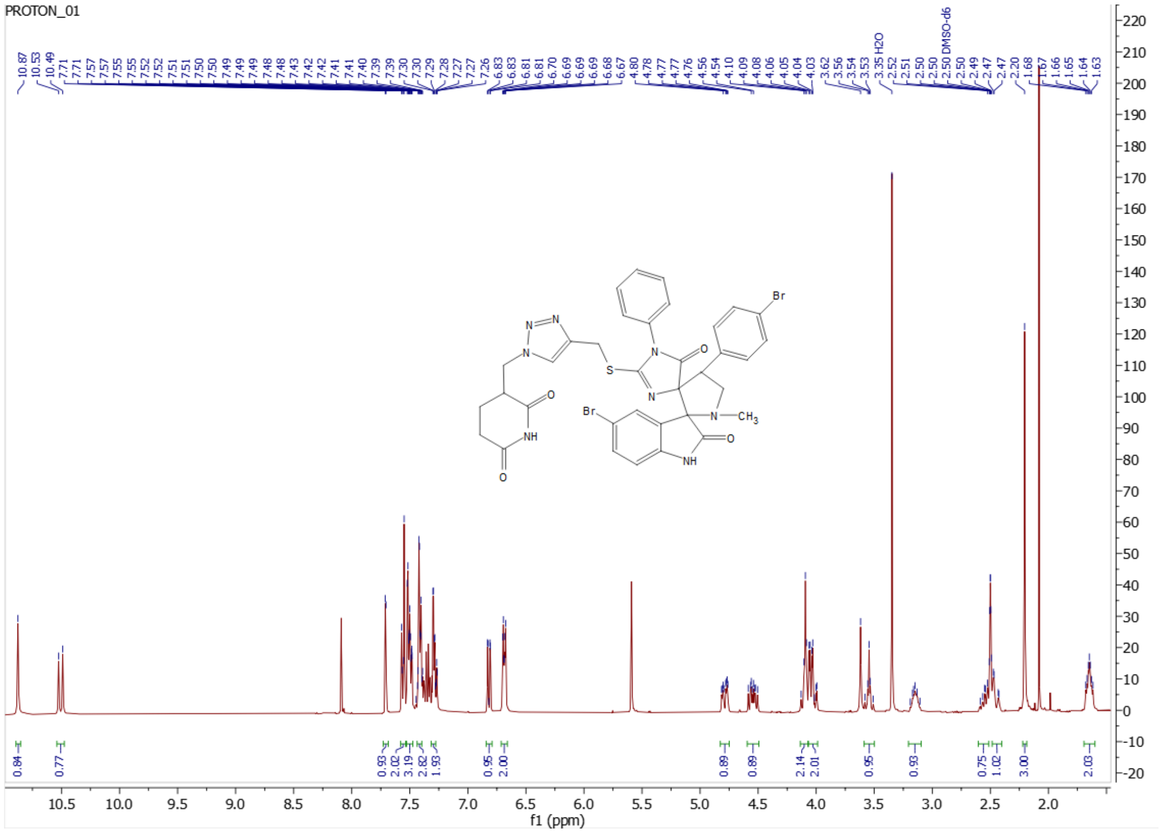

Figure S21. ^{1}H NMR spectrum of compound **6b**

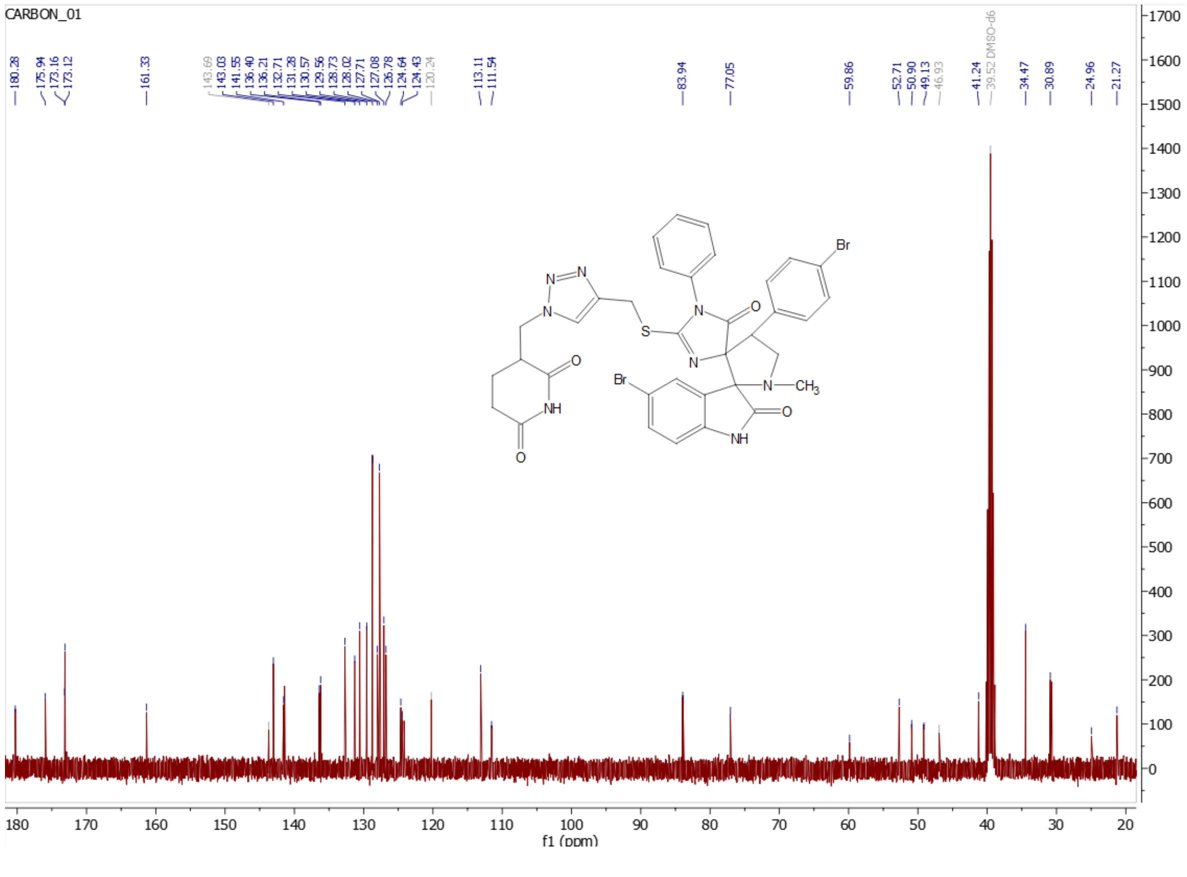

Figure S22. ^{1}H NMR spectrum of compound **6b**

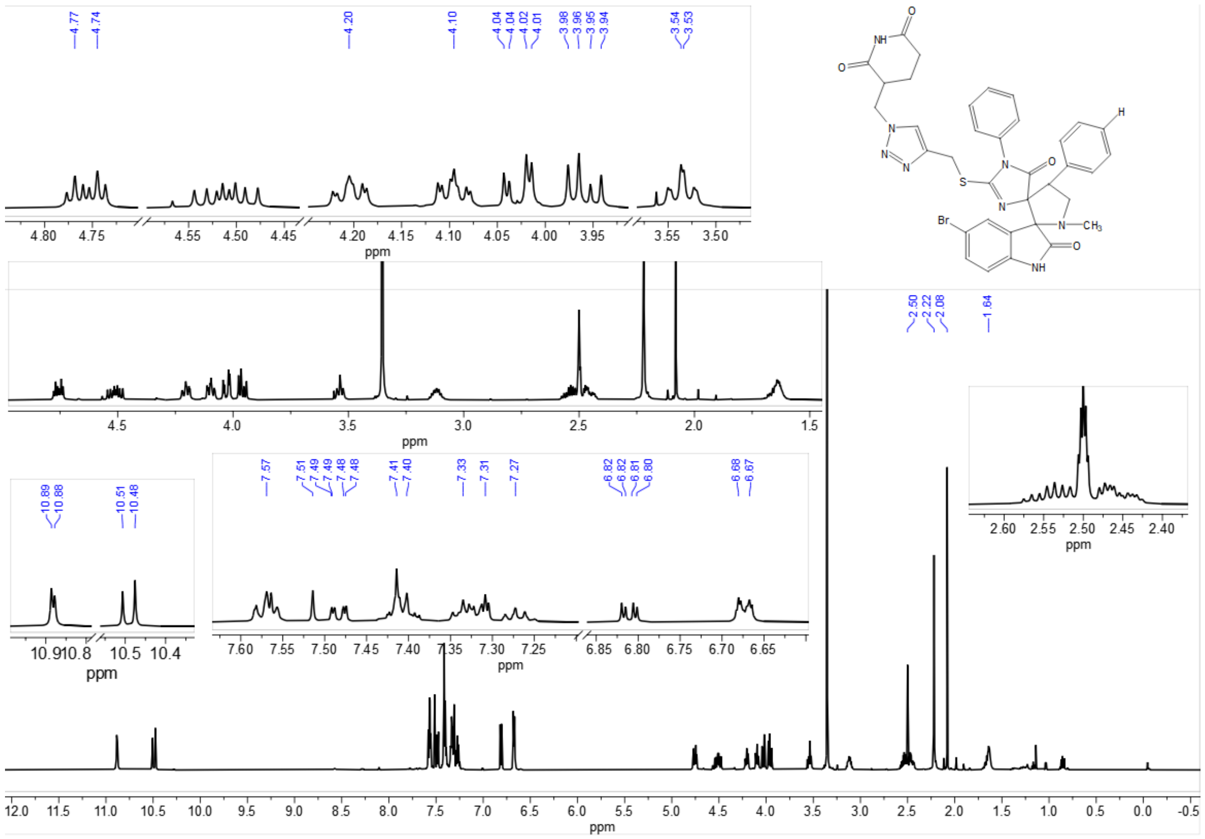

Figure S23. ^{1}H NMR (600 MHz) spectra of compound **6c**

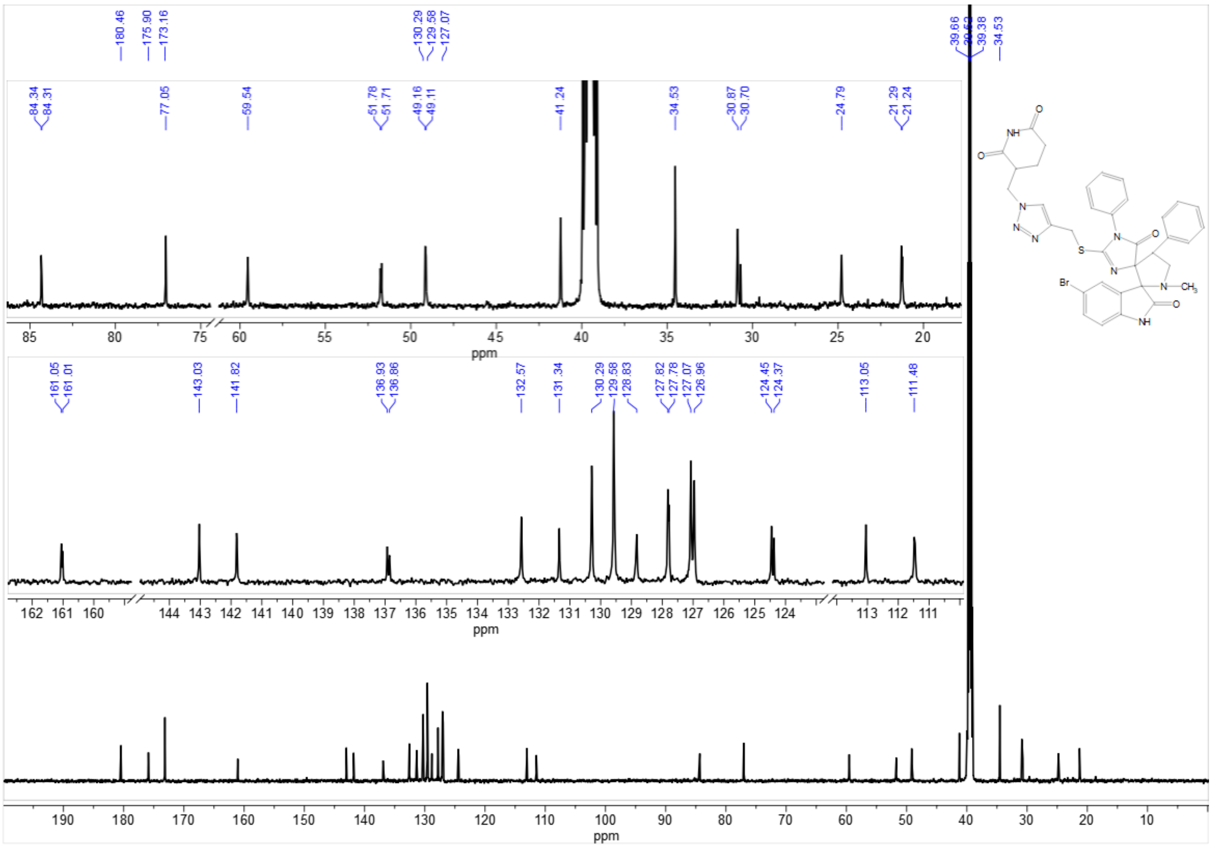

Figure S24. ^{13}C NMR (151 MHz) spectrum of compound **6c**

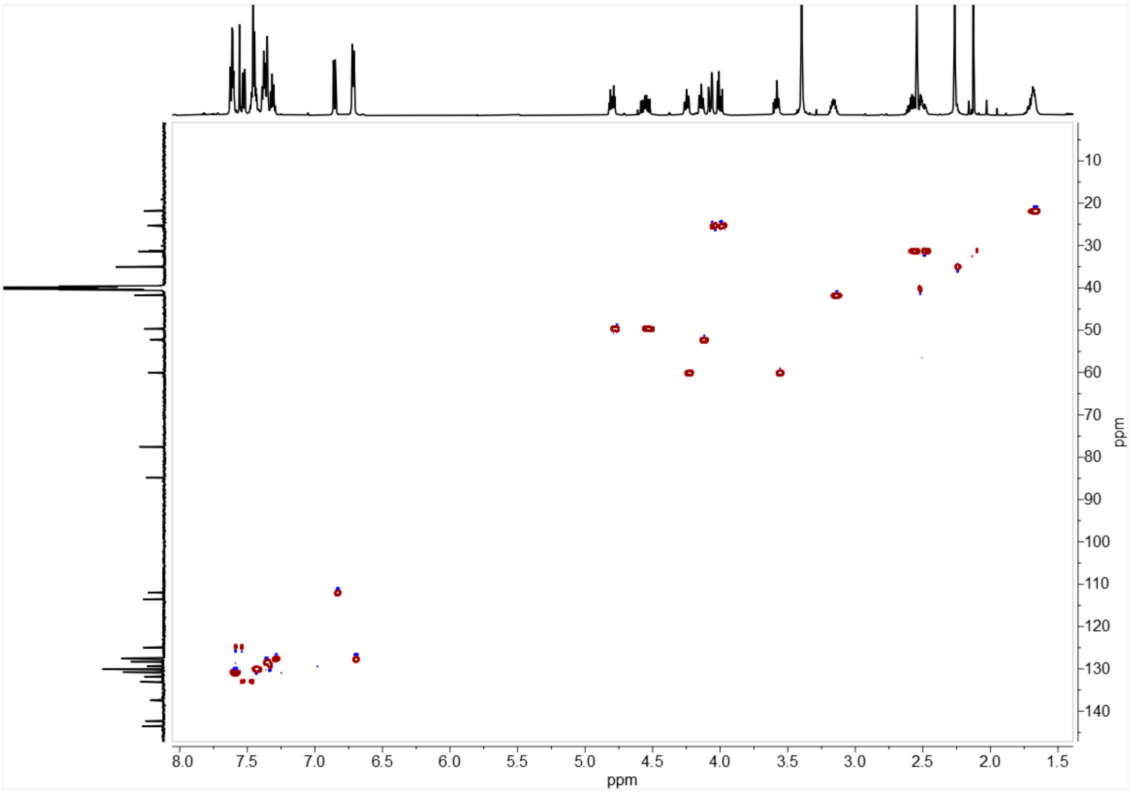

Figure S25. ^{1}H-^{13}C HSQC spectrum of compound **6c**

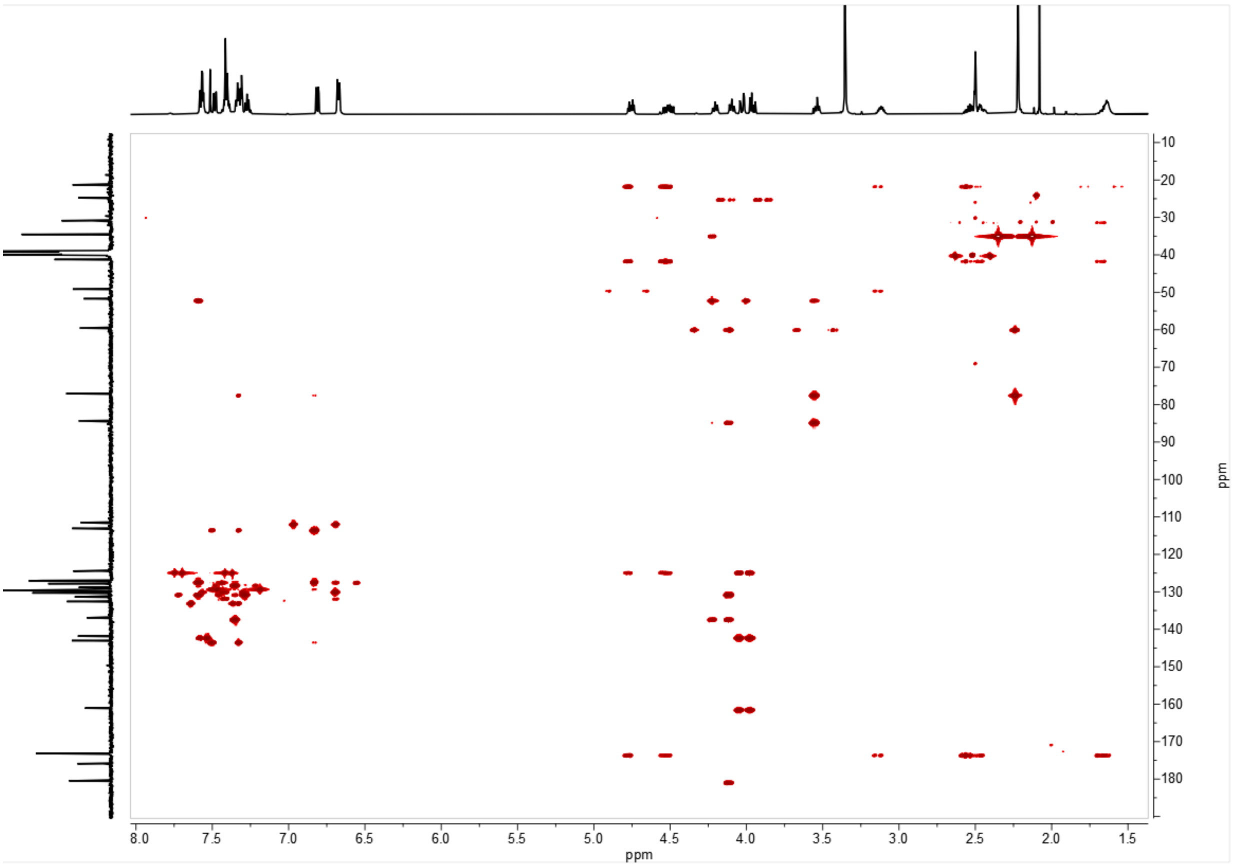

Figure S26. ^{1}H-^{13}C HMBC spectrum of compound **6c**

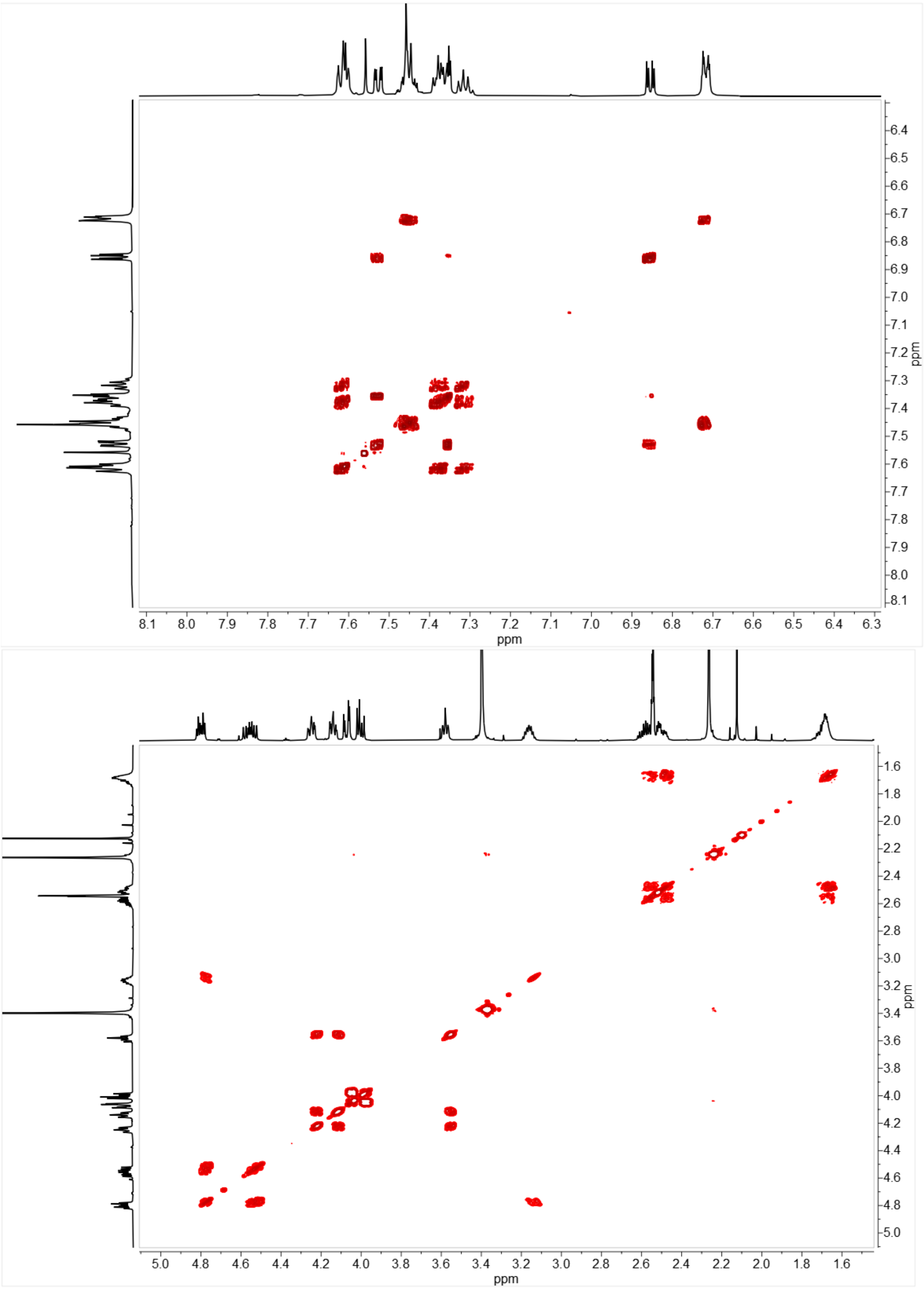

Figure S27. ^{1}H-^{1}H COSY spectra of compound **6c**

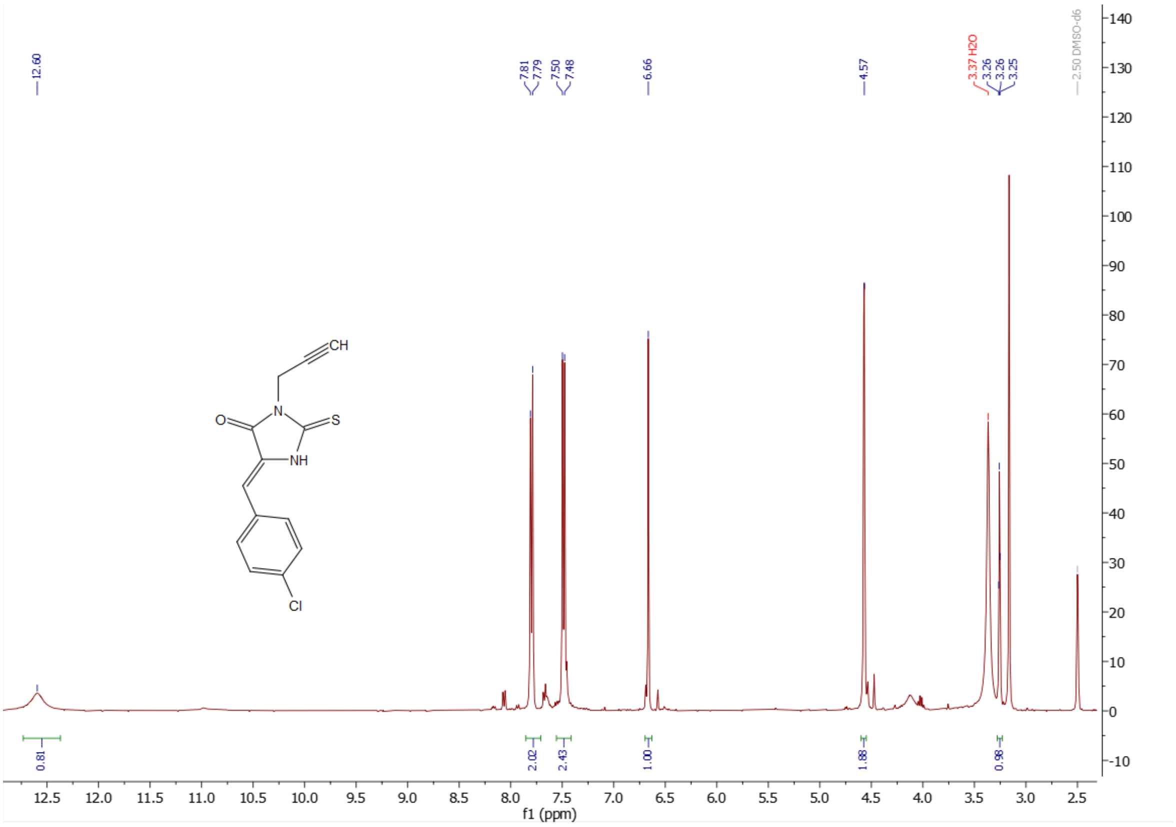

Figure S28. ^{1}H NMR spectrum of the compound **8a**

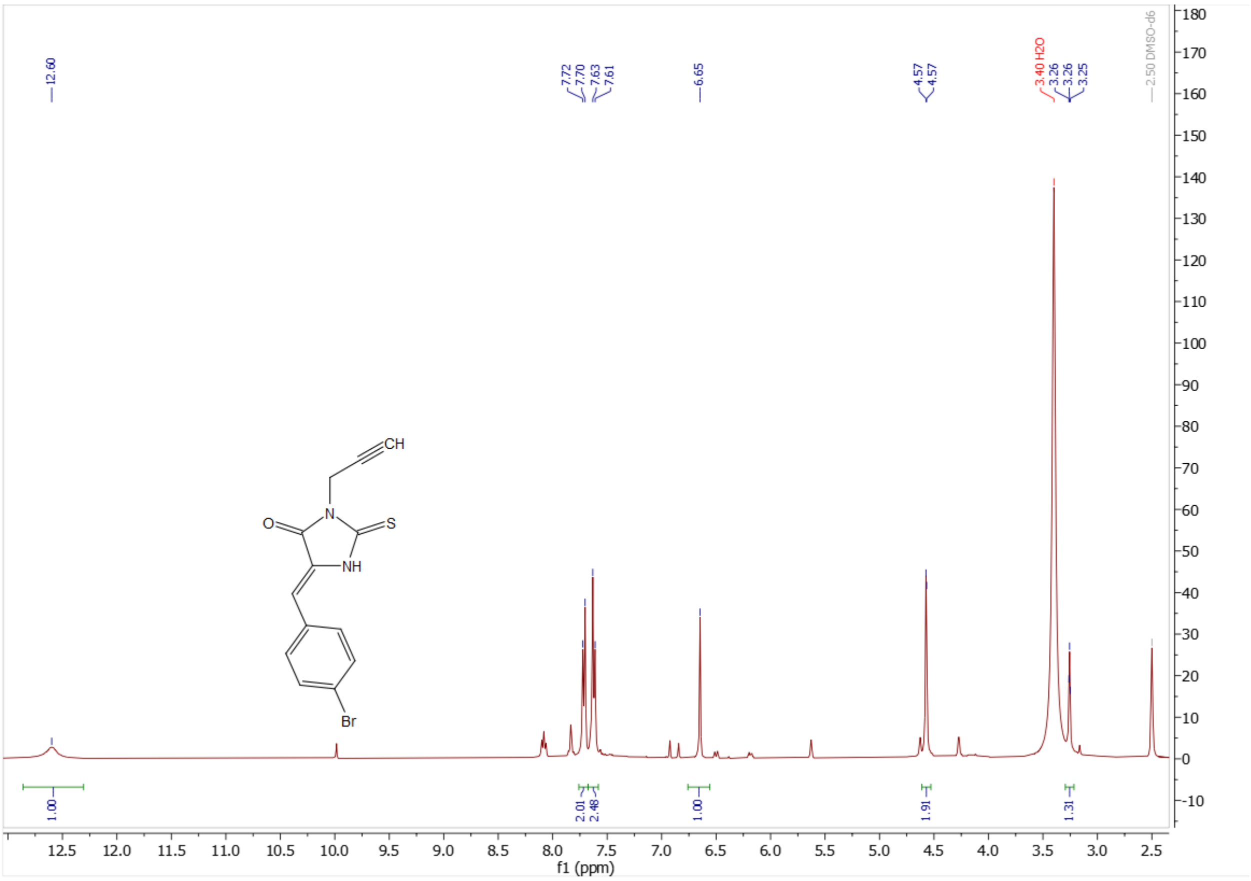

Figure S29. ^{1}H NMR spectrum of the compound **8b**

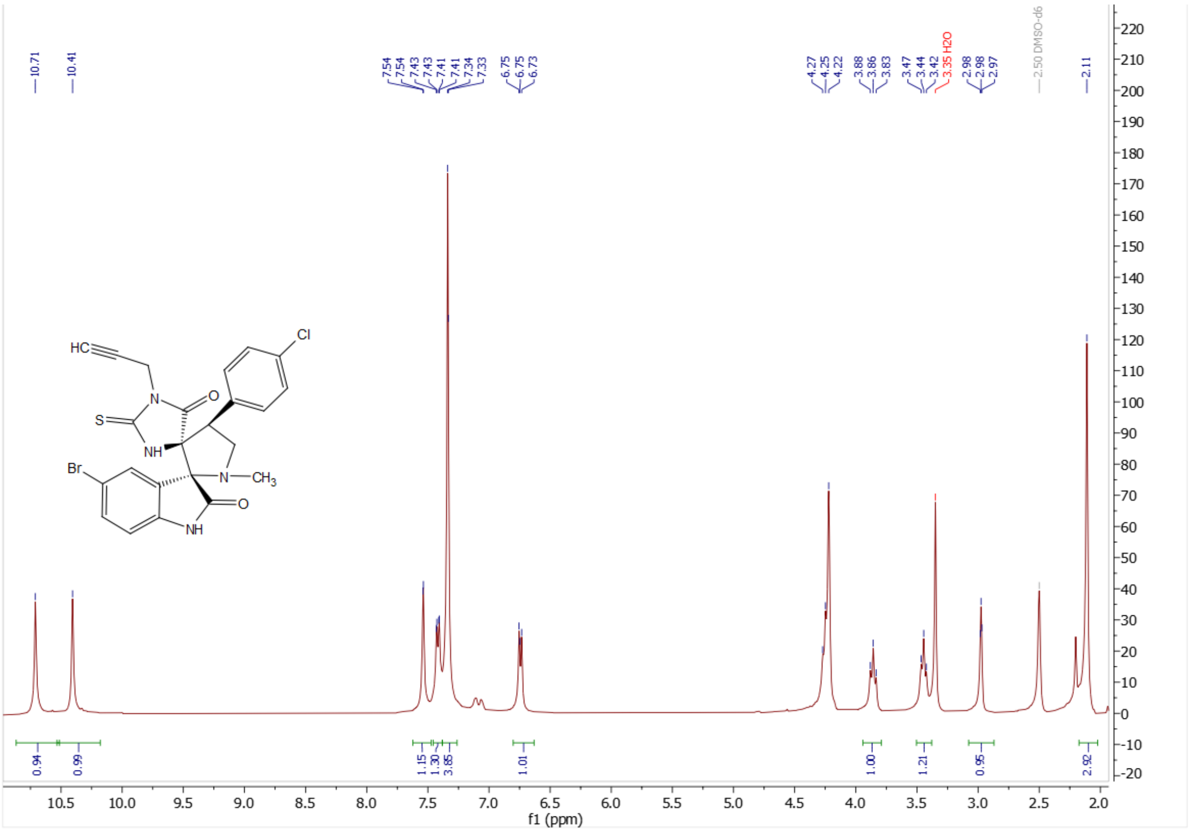

Figure S30. ^{1}H NMR spectrum of the compound **9a**

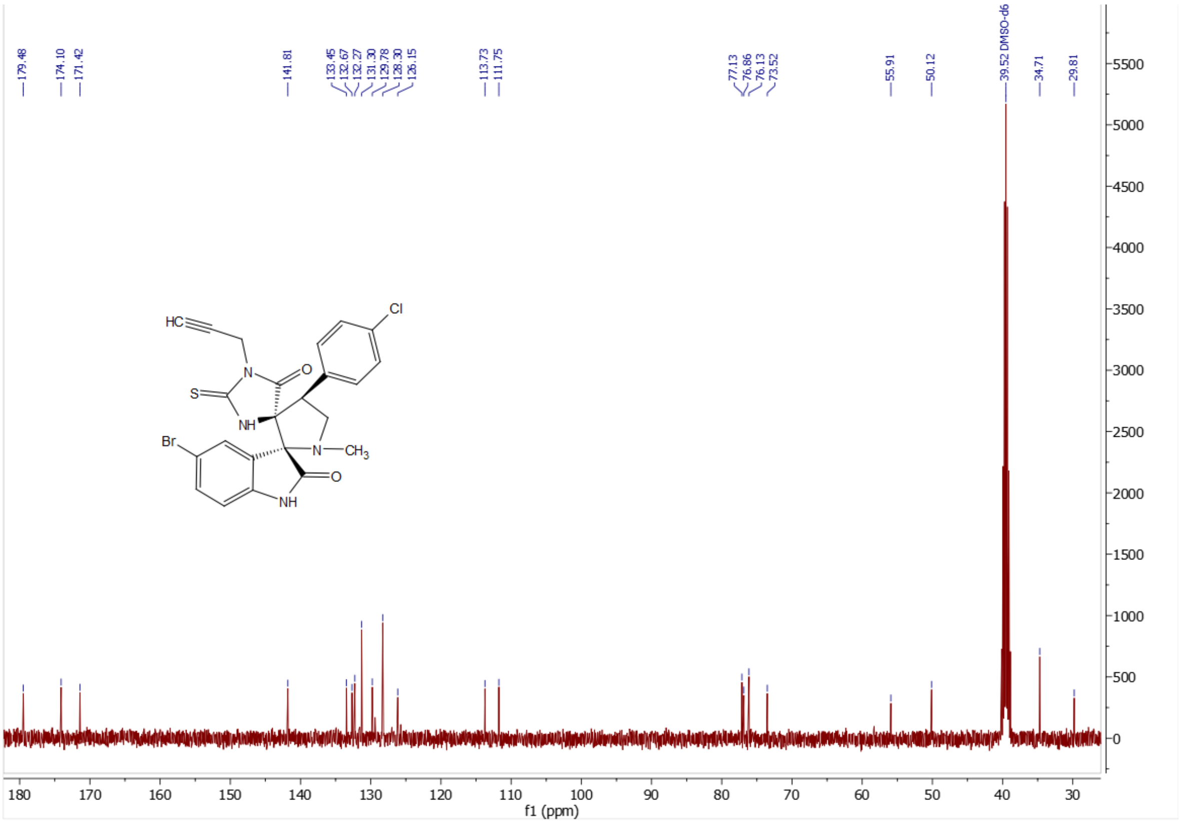

Figure S31. ^{13}C NMR spectrum spectra of the compound **9a**

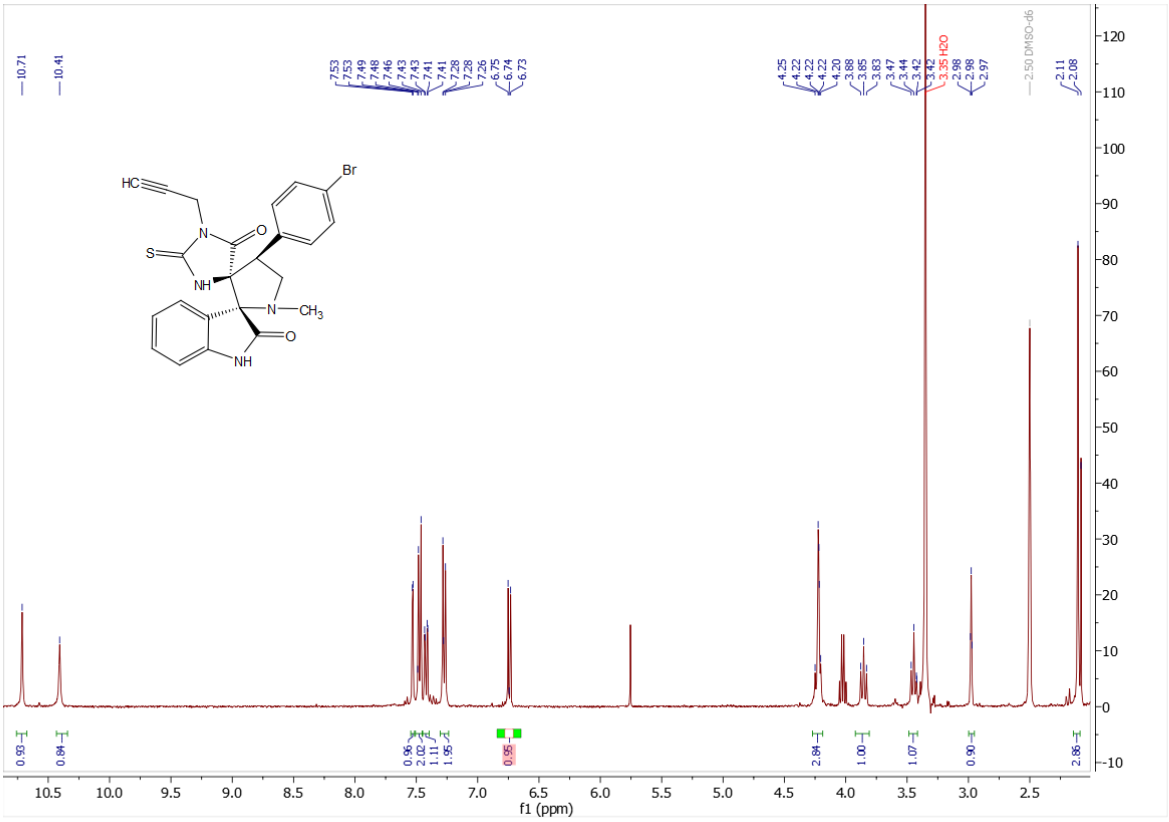

Figure S32. ^{1}H NMR spectrum spectra of the compound **9b**

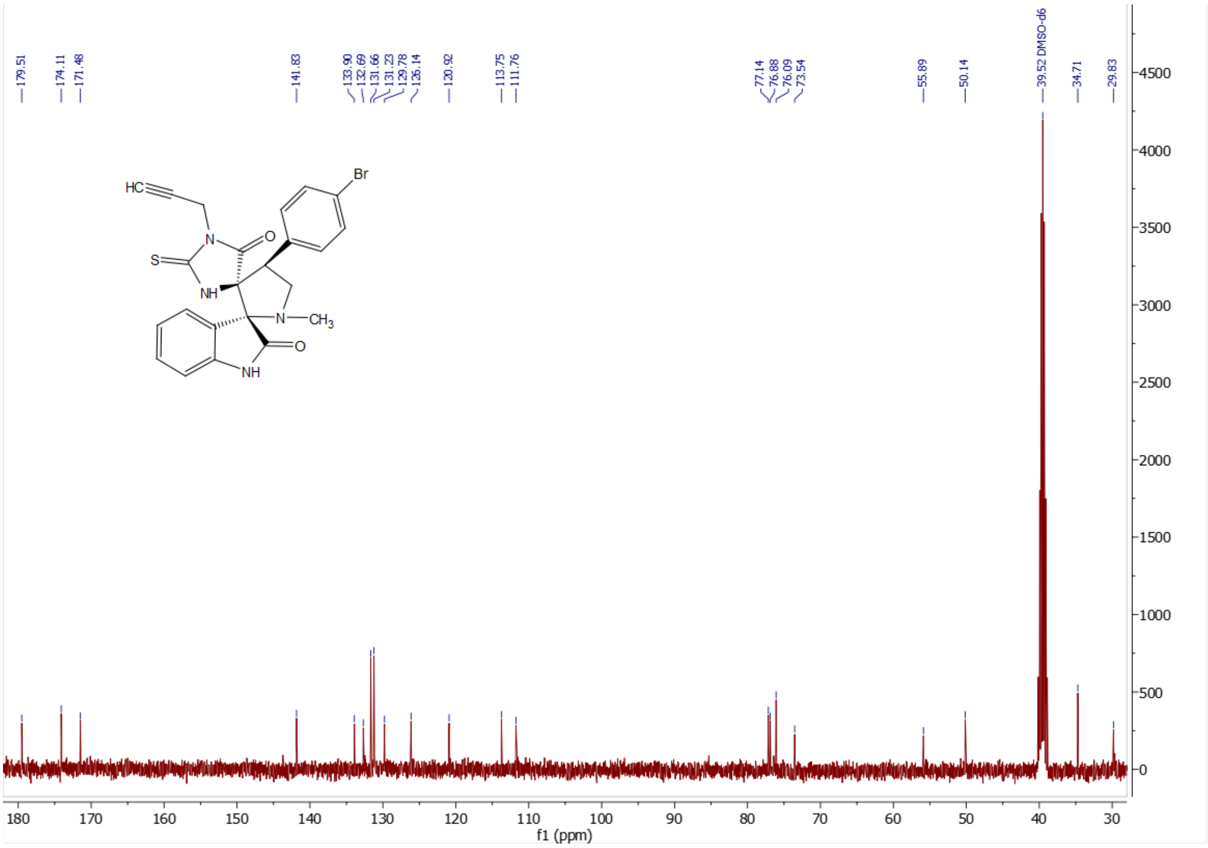

Figure S33. ^{13}C NMR spectrum of the compound **9b**

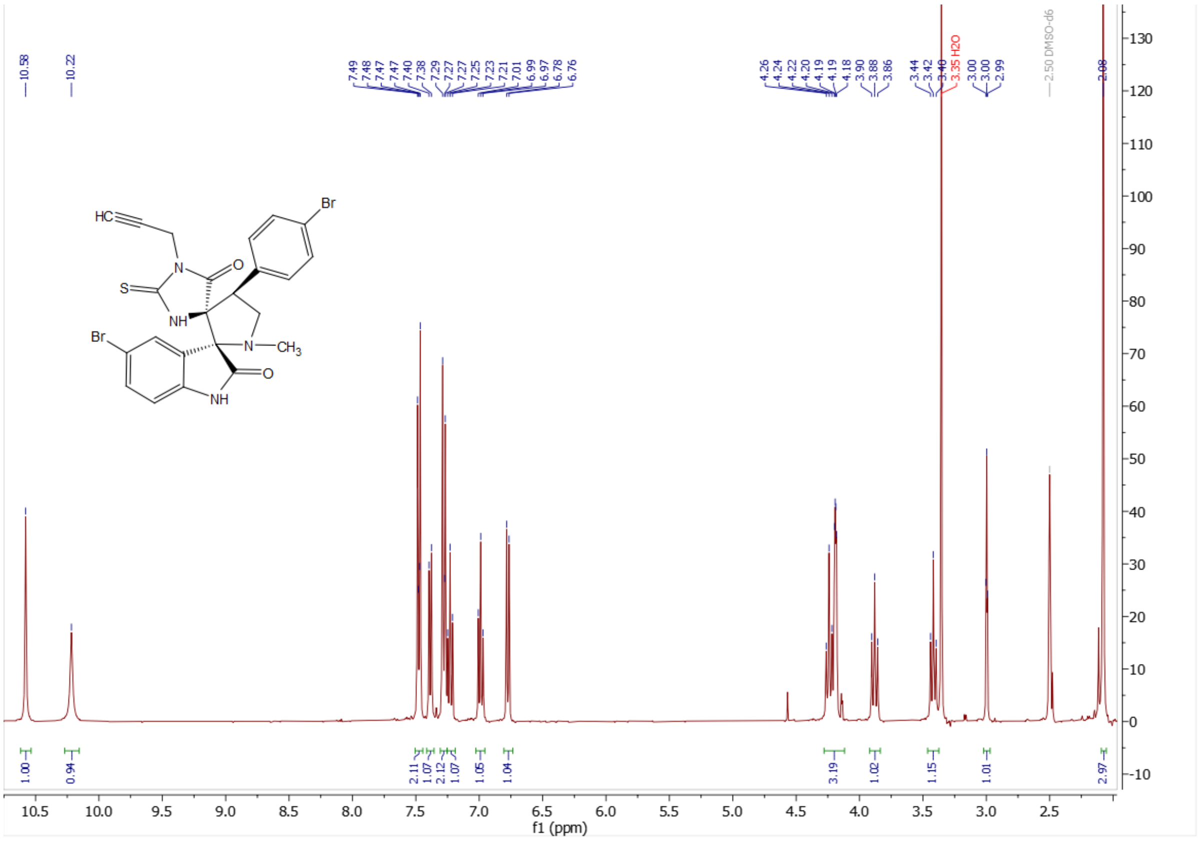

Figure S34. ^{1}H NMR spectrum spectra of the compound **9c**

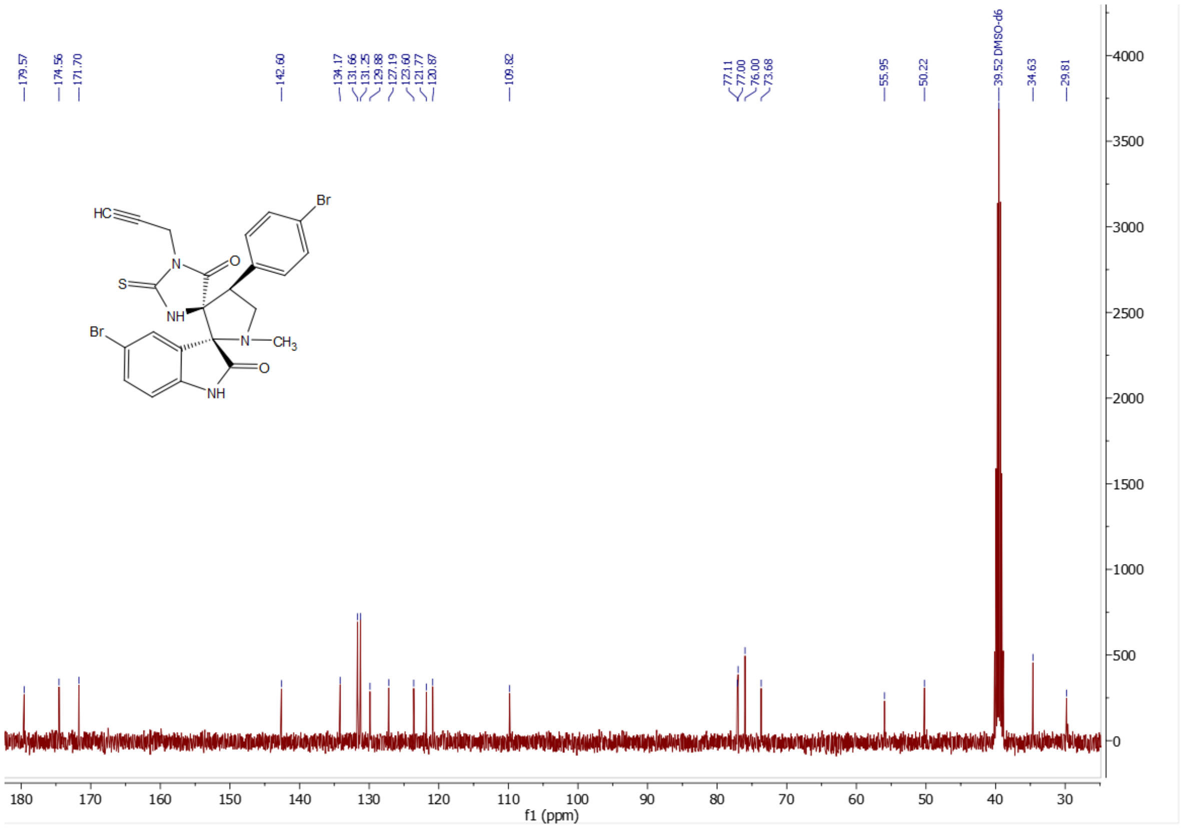

Figure S35. ^{13}C NMR spectrum of the compound **9c**

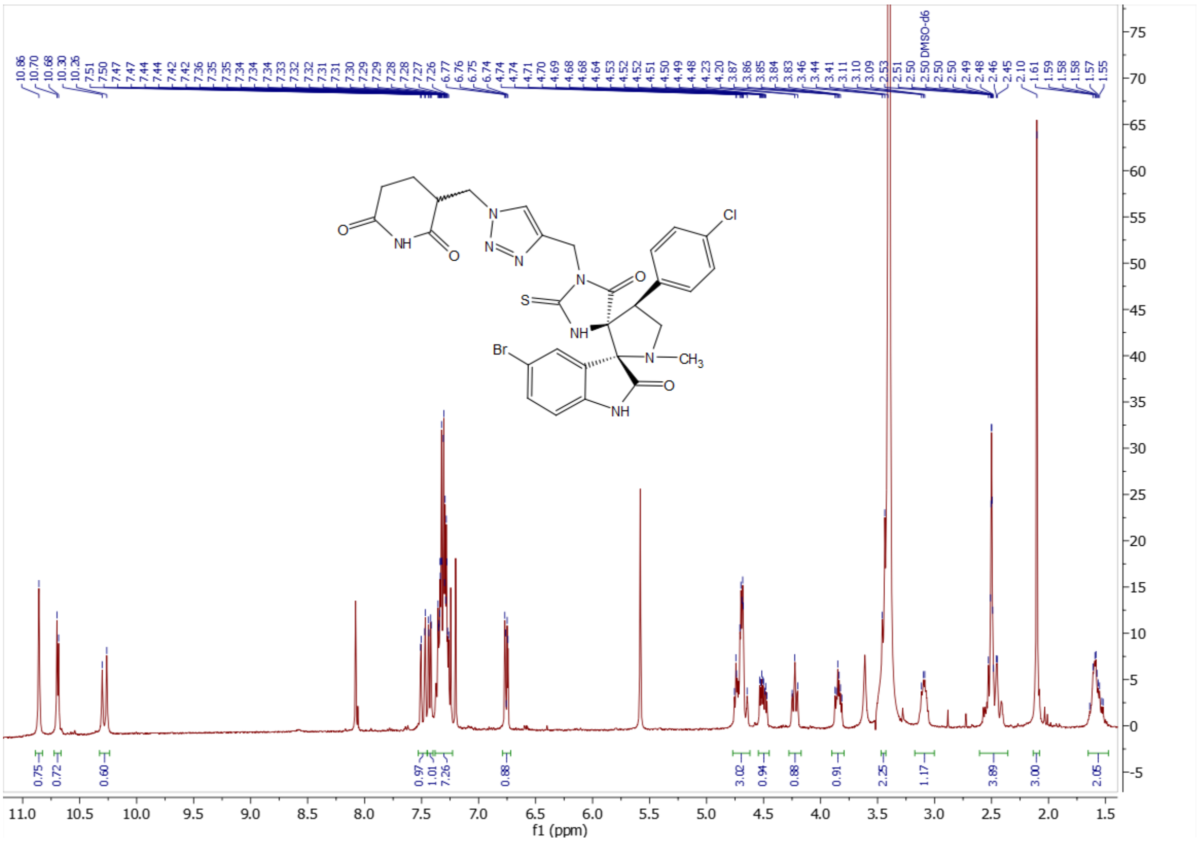

Figure S36. ^{1}H NMR spectrum of the compound **10a**

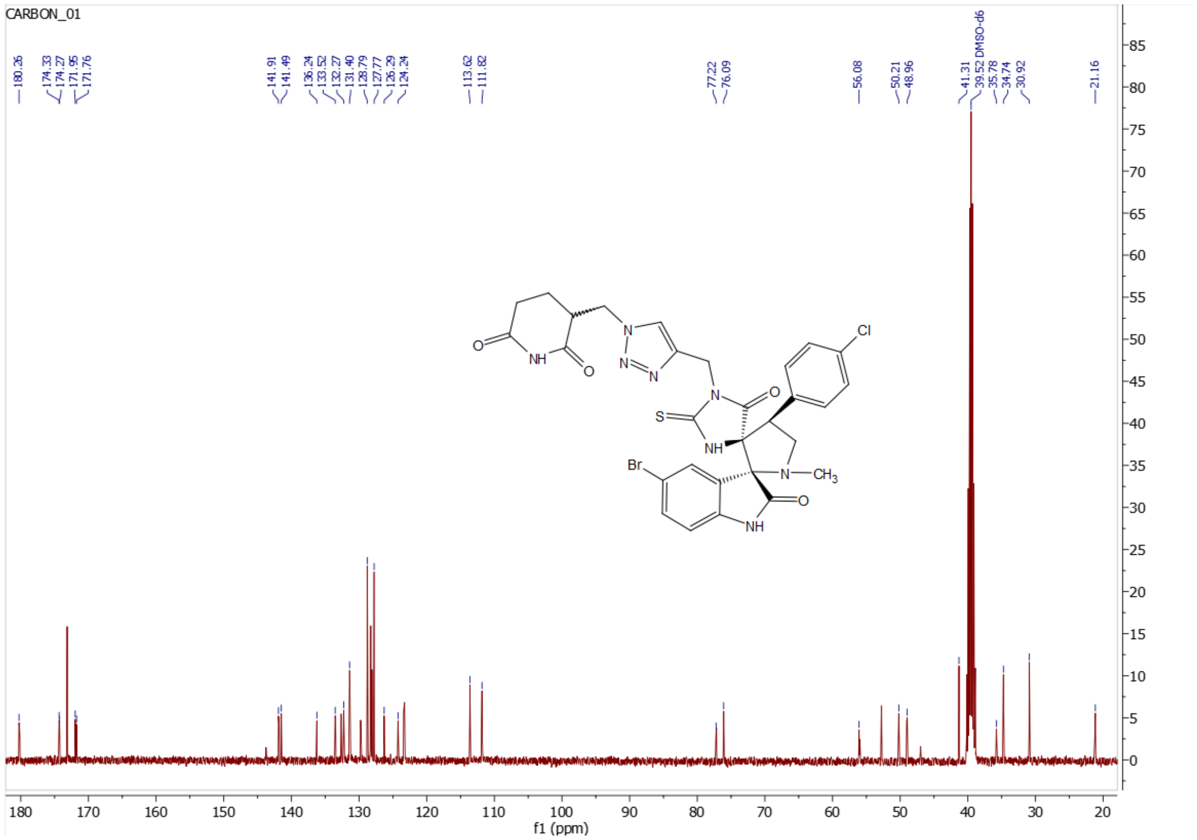

Figure S37. ^{13}C NMR spectrum of the compound **10a**

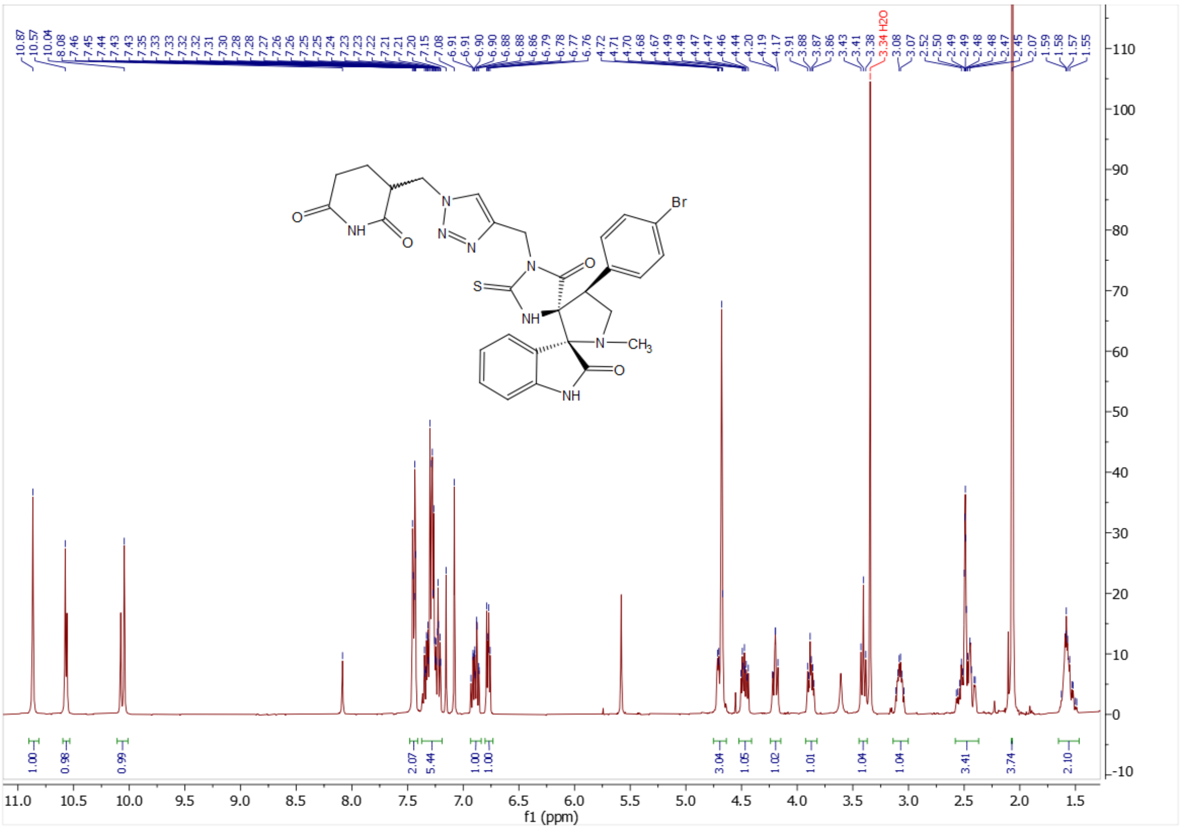

Figure S38. ^{1}H NMR spectrum of the compound **10b**

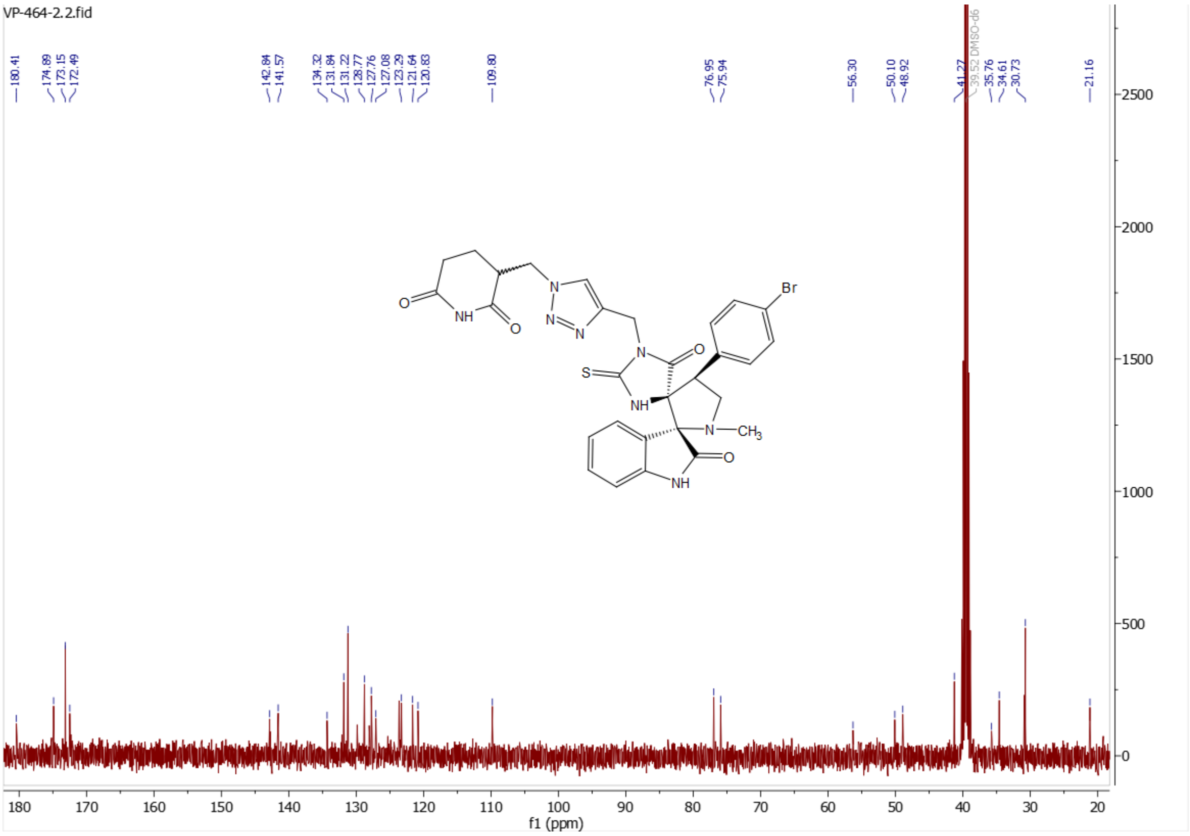

Figure S39. ^{13}C NMR spectrum of the compound **10b**

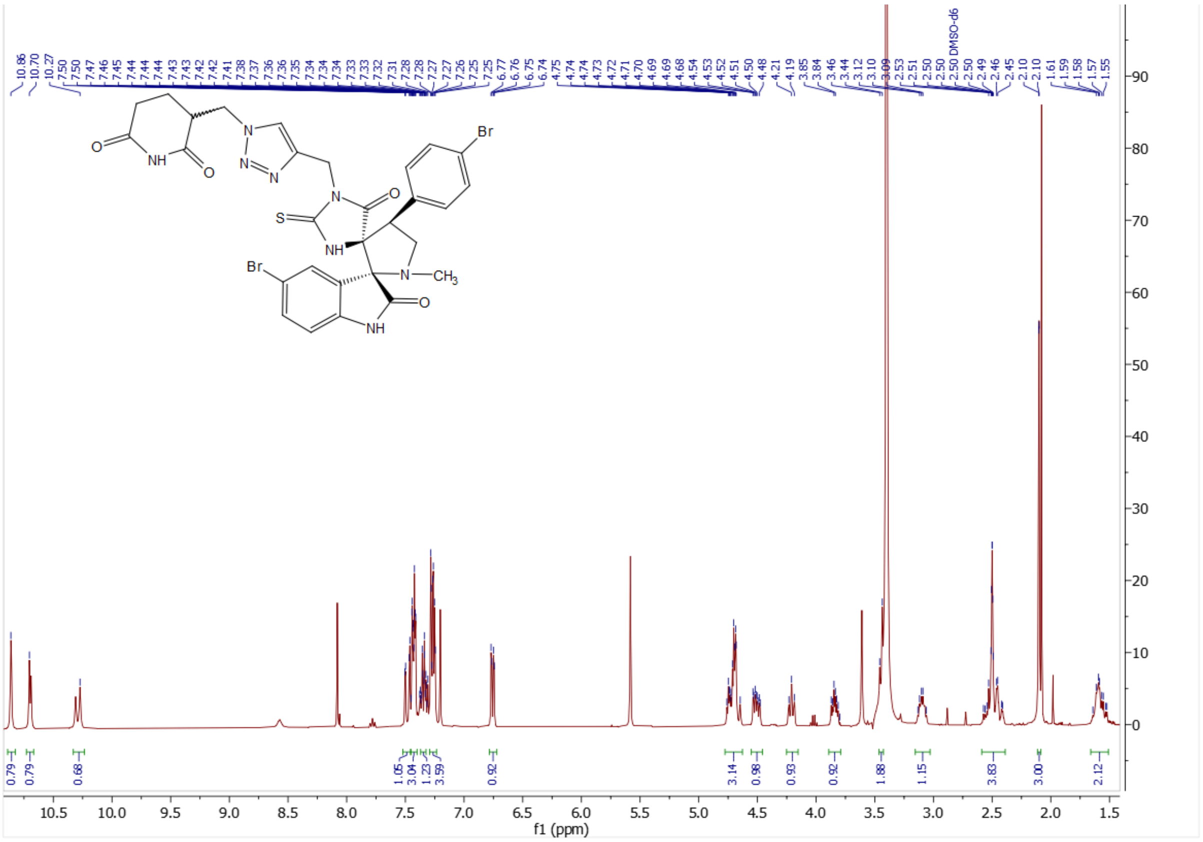

Figure S40. ^{1}H NMR spectrum of the compound **10c**

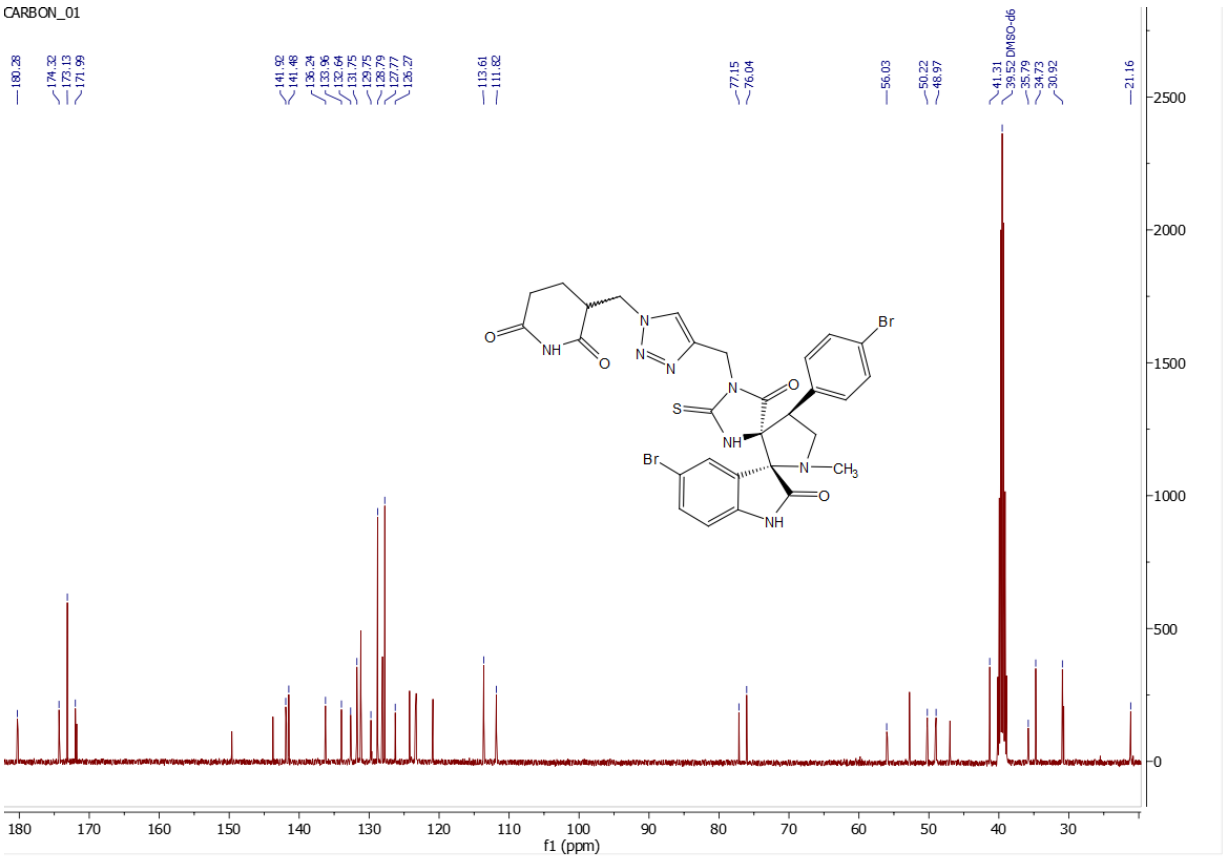

Figure S41. ^{13}C NMR spectrum of the compound **10c**

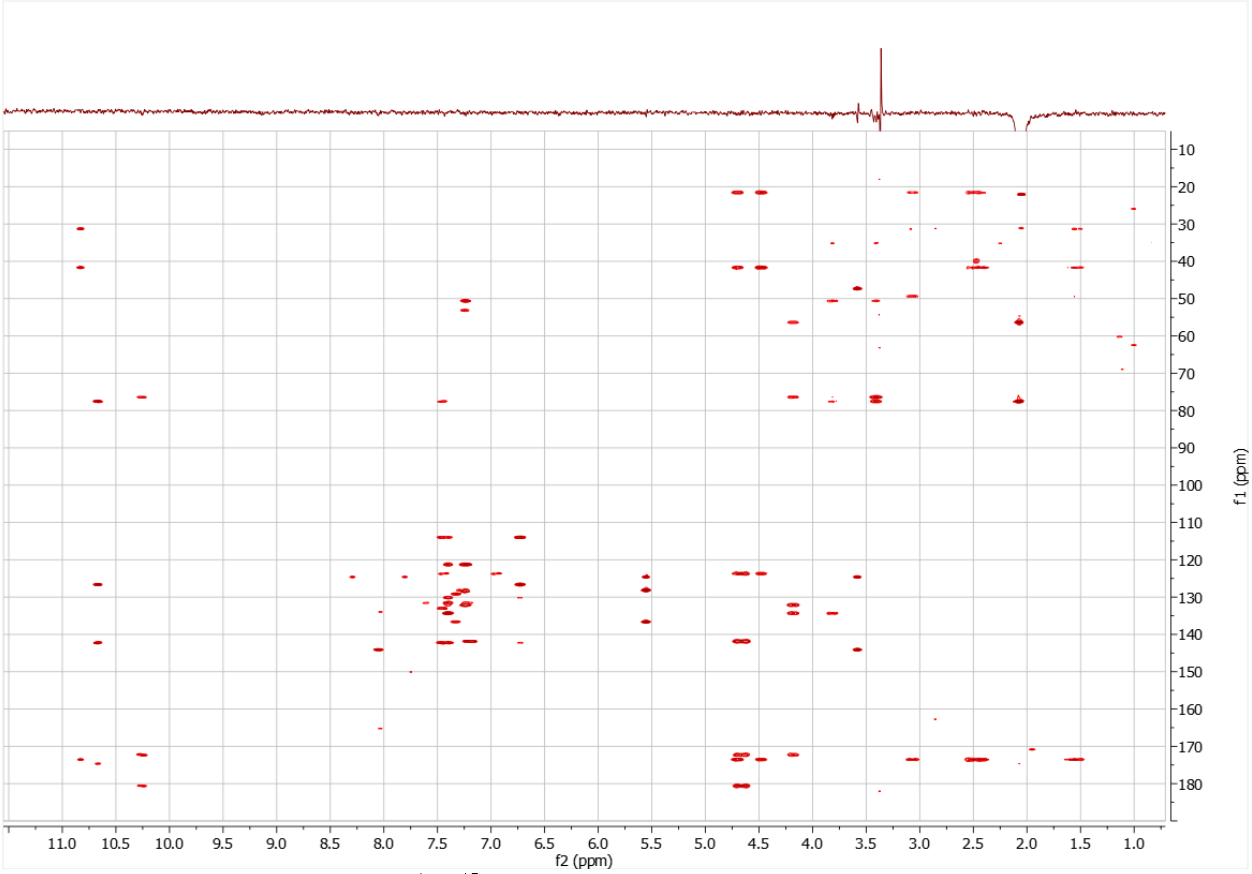

Figure S42. ^{1}H-^{13}C HMBC spectrum of the compound **10c**

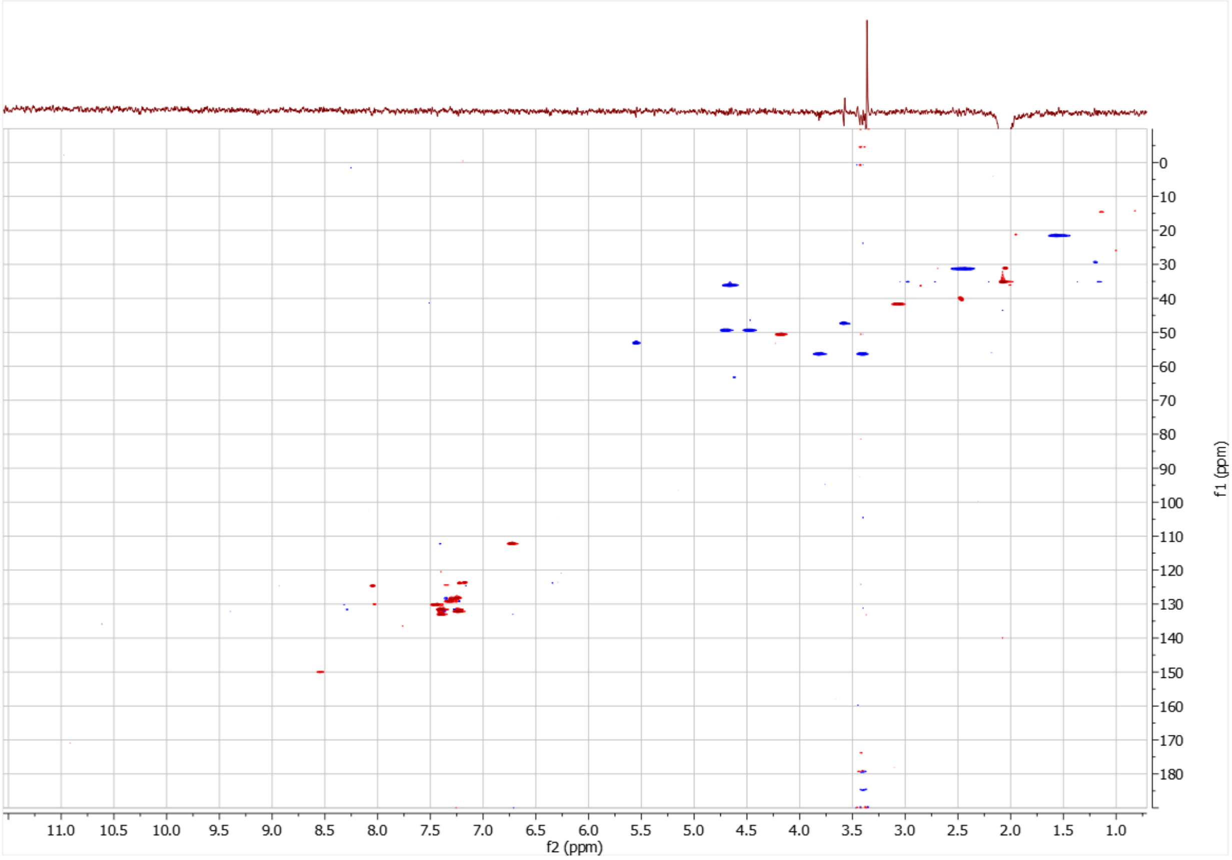

Figure S43. ^{1}H-^{13}C HSQC spectrum of the compound **10c**

2. IR Spectra

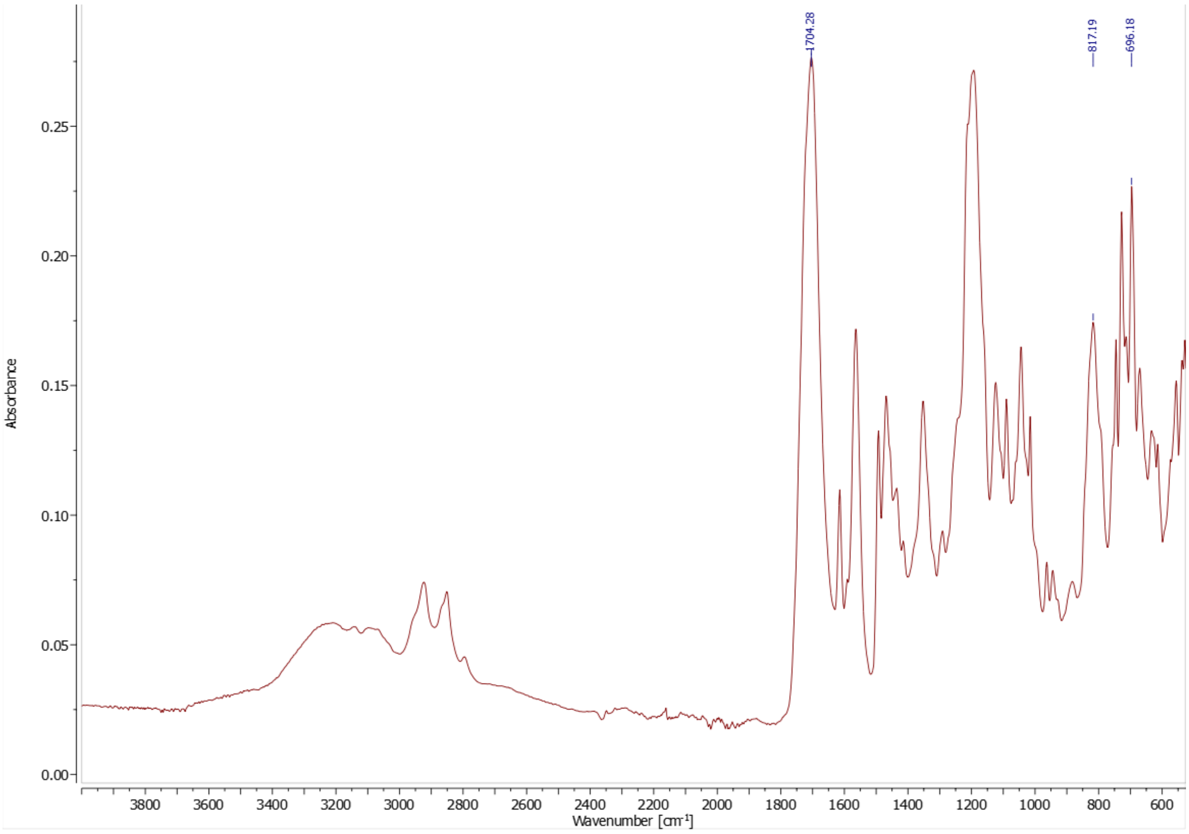

Figure S44. IR spectrum of compound **6a**

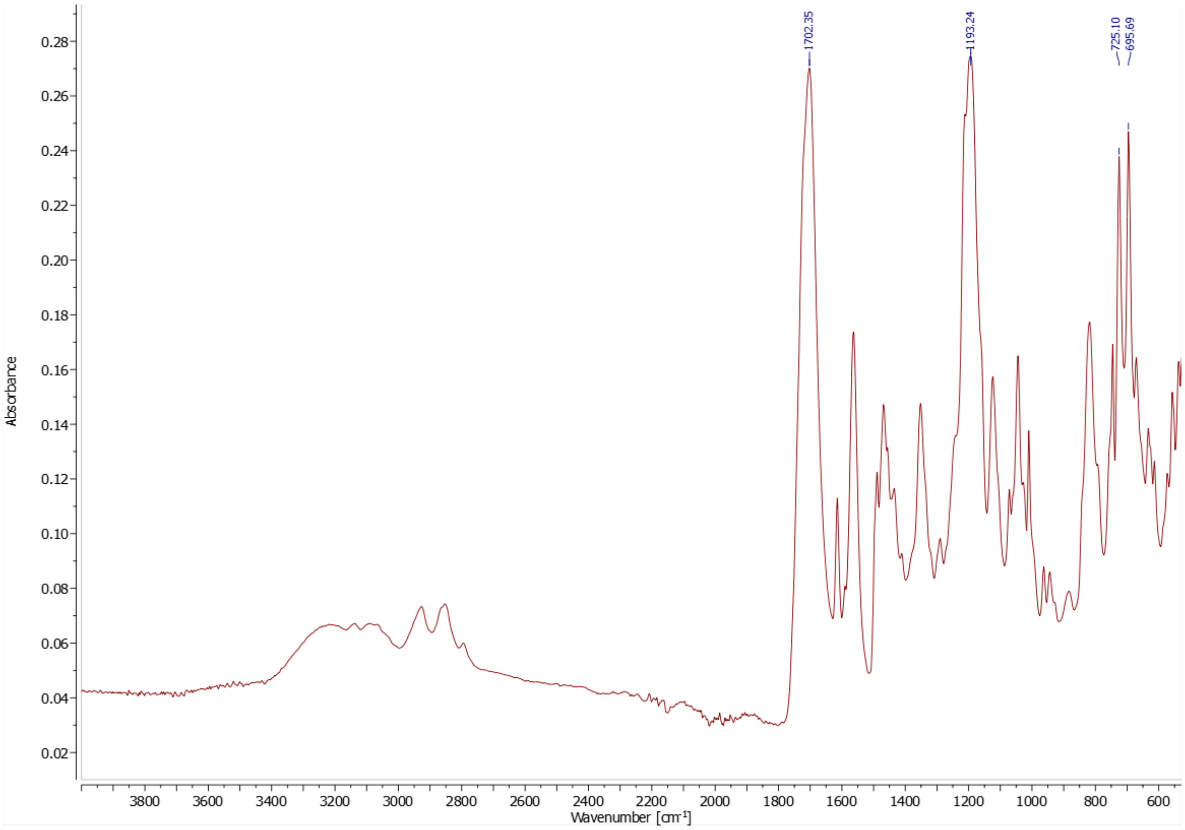

Figure S45. IR spectrum of compound **6b**

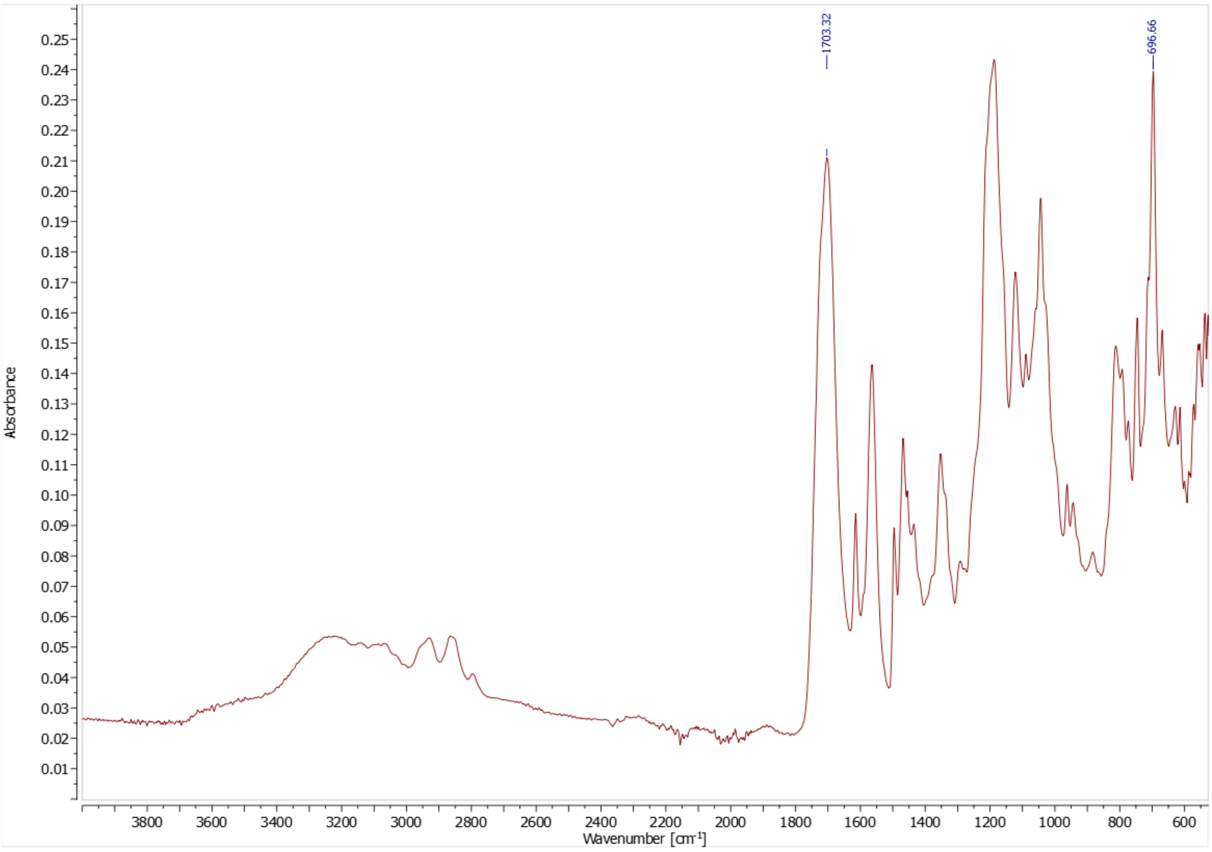

Figure S46. IR spectrum of compound **6c**

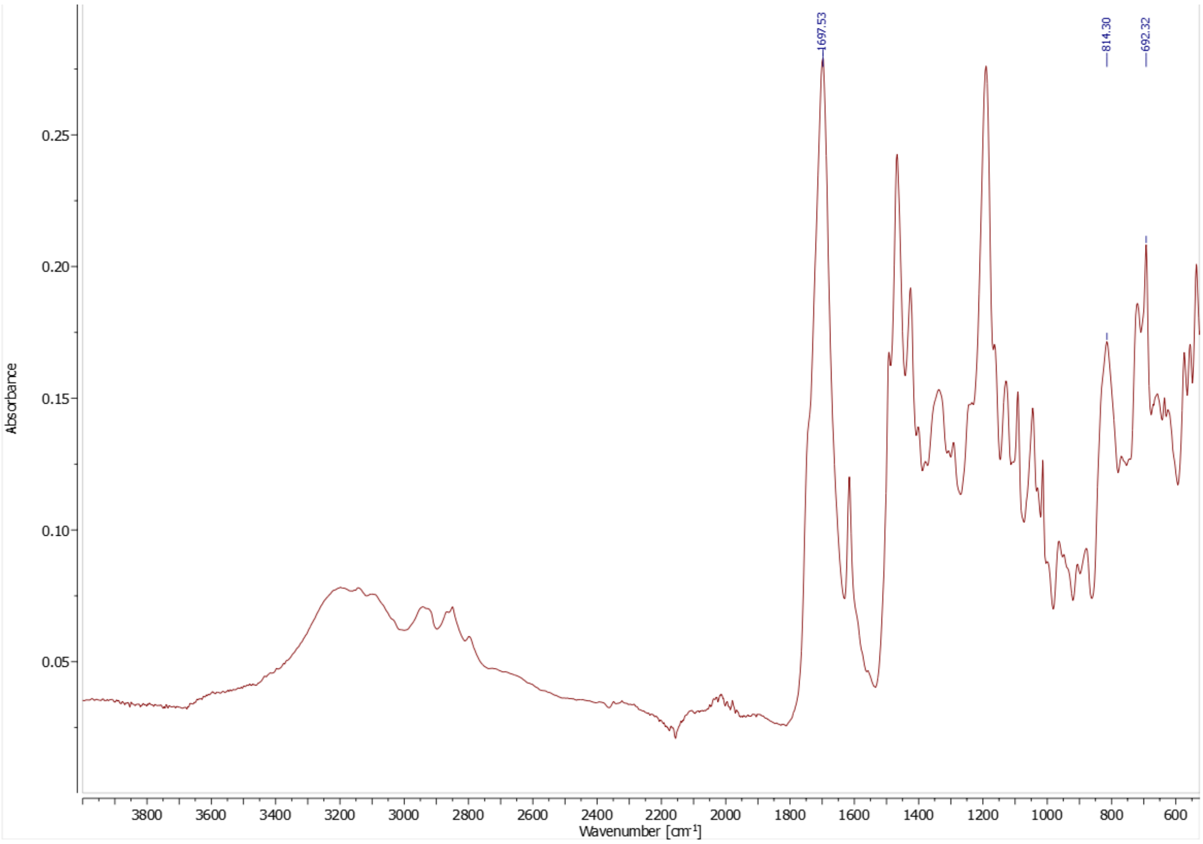

Figure S47. IR spectrum of compound **10a**

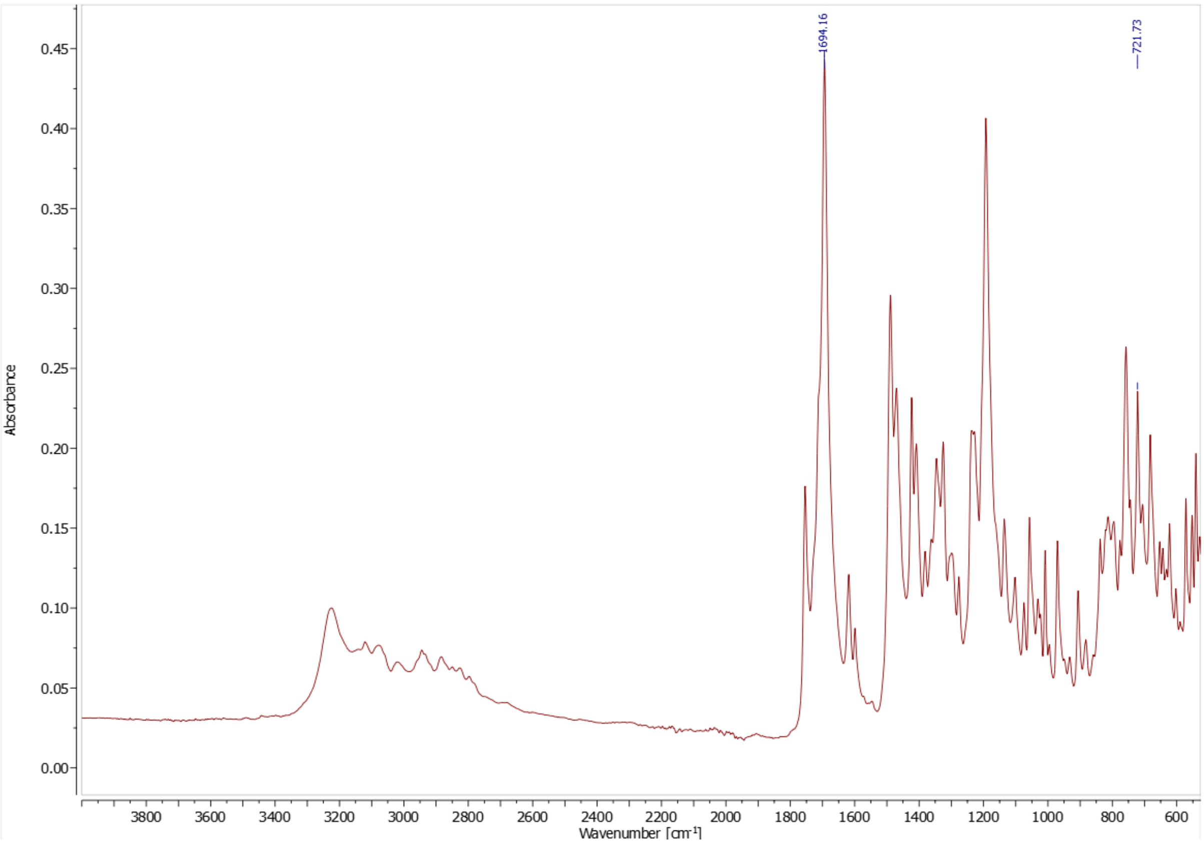

Figure S48. IR spectrum of compound **10b**

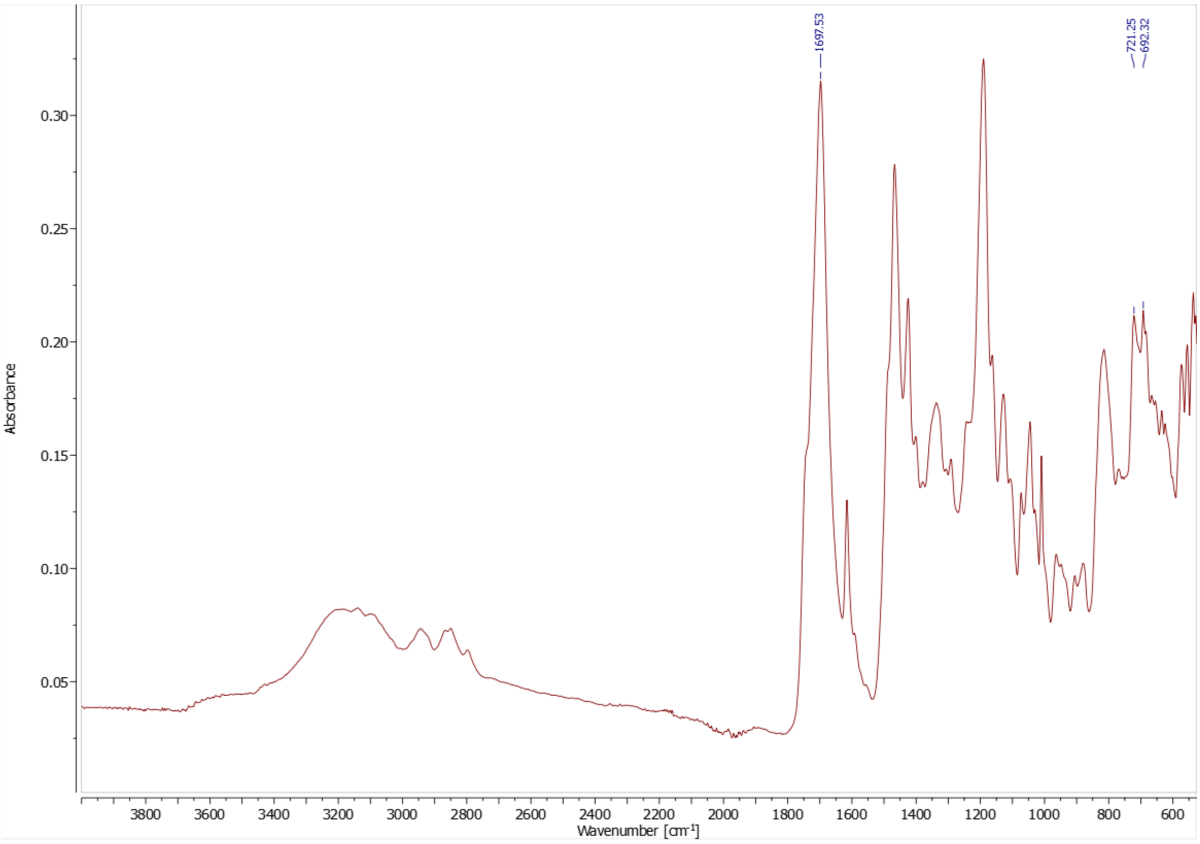

Figure S49. IR spectrum of compound **10c**

3. HPLC

HPLC analysis were performed with a Hitachi LaChrome Elite-2000 chromatograph with UV detector using a Daicel AD-H column (0.46 × 25 cm) at room temperature, flow rate 1.0 mL/min, peak detection at 254 nm.

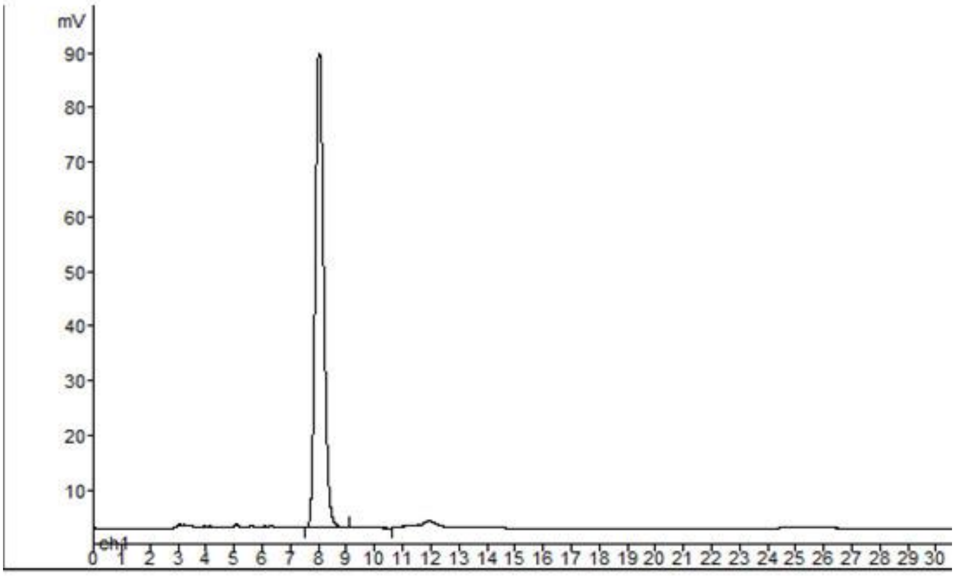

Figure S50. HPLC of compound **6a**

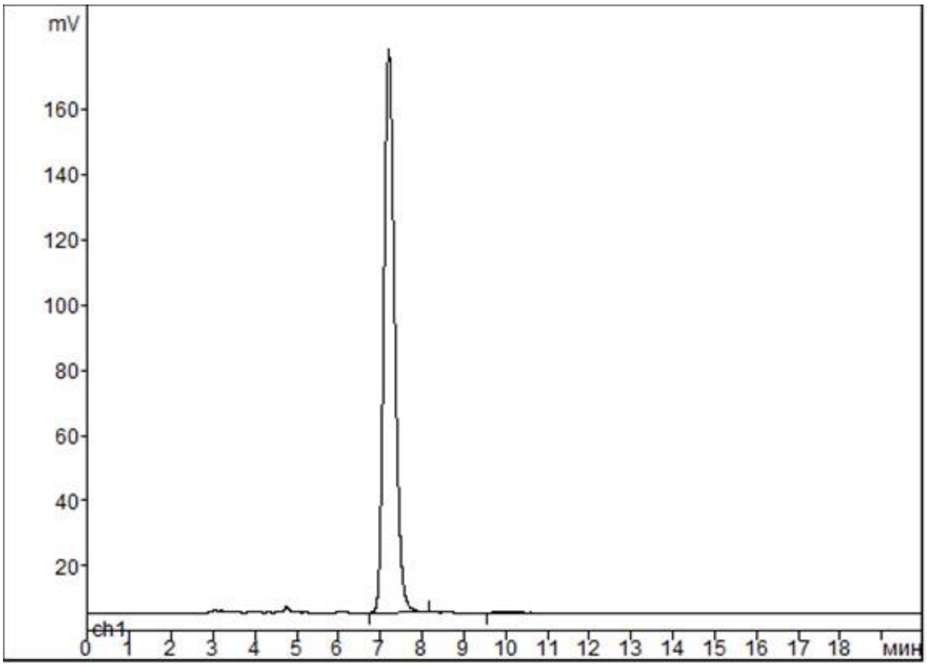

Figure S51. HPLC of compound **6b**

4. Biological testing

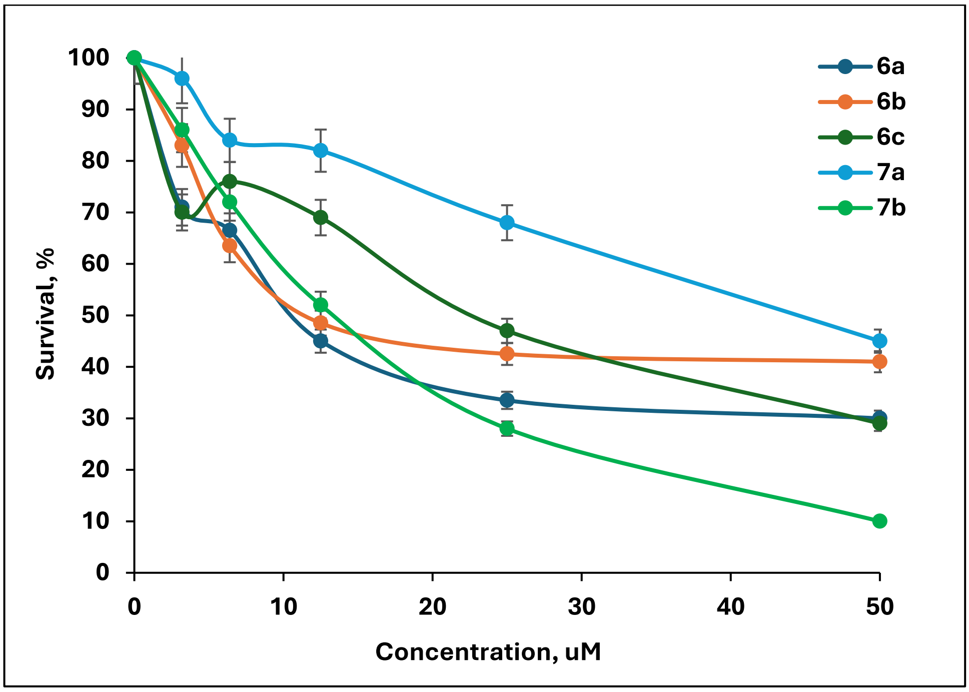

Figure S52. Survival curves for HCT116 cells (wild type p53).

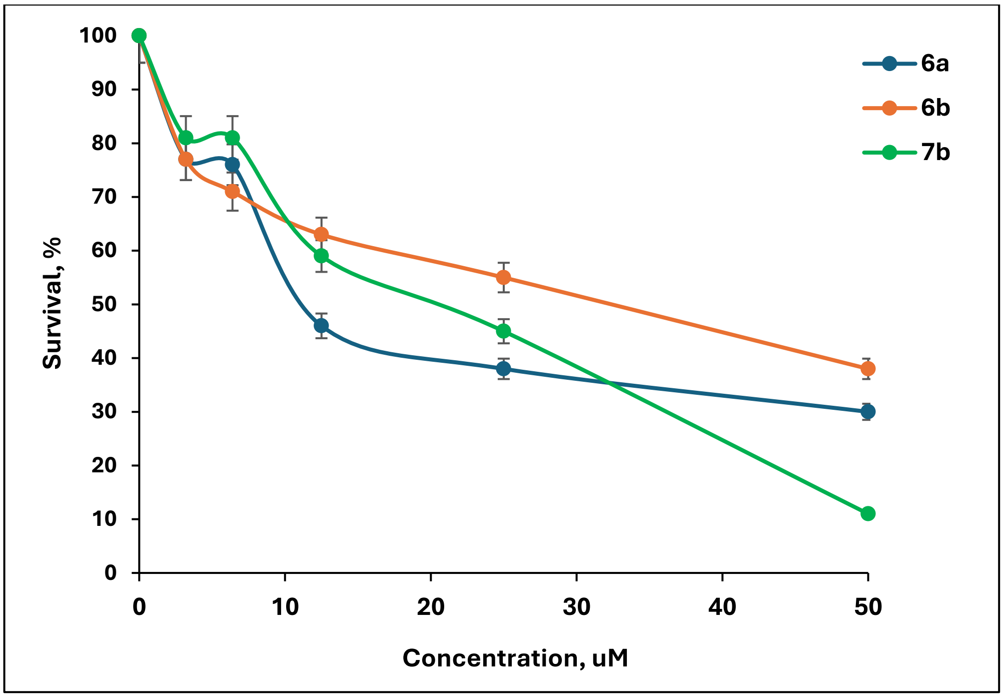

Figure S53. Survival curves for HCT116p53$^{-/-}$ subline.

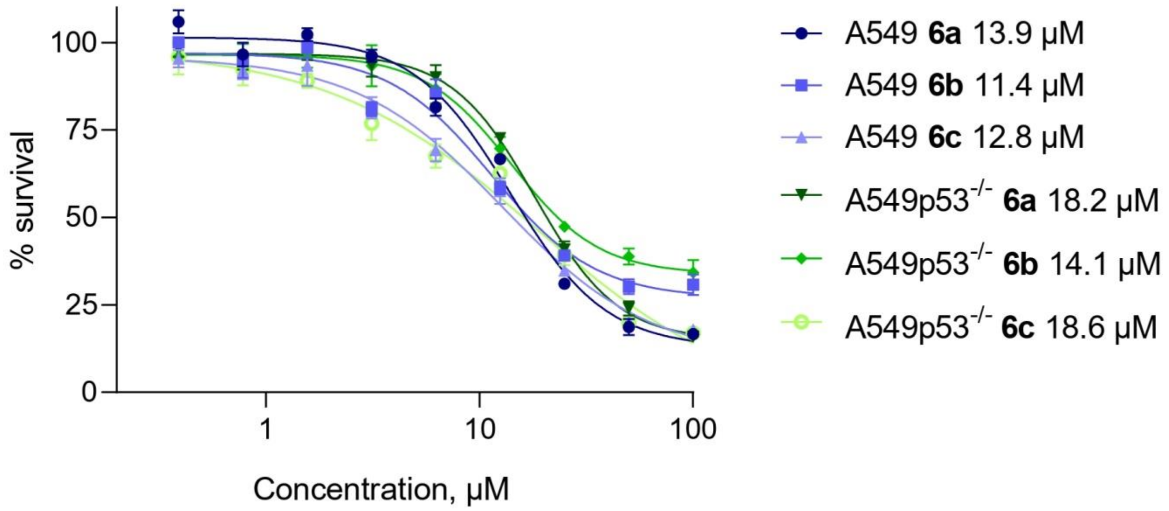

Figure S54. Survival curves for A549 cell line (wild type p53) and A549p53$^{-/-}$ subline.
